# Evolution of the Muscarinic Acetylcholine Receptors in Vertebrates

**DOI:** 10.1523/ENEURO.0340-18.2018

**Published:** 2018-11-08

**Authors:** Julia E. Pedersen, Christina A. Bergqvist, Dan Larhammar

**Affiliations:** Department of Neuroscience, Unit of Pharmacology, Science for Life Laboratory, Uppsala University, Uppsala SE-751 24, Sweden

**Keywords:** acetylcholine, G-protein-coupled receptor, gene duplication, muscarinic, tetraploidization, zebrafish

## Abstract

The family of muscarinic acetylcholine receptors (mAChRs) consists of five members in mammals, encoded by the *CHRM1*-5 genes. The mAChRs are G-protein-coupled receptors, which can be divided into the following two subfamilies: M2 and M4 receptors coupling to G_i/o_; and M1, M3, and M5 receptors coupling to G_q/11_. However, despite the fundamental roles played by these receptors, their evolution in vertebrates has not yet been fully described. We have combined sequence-based phylogenetic analyses with comparisons of exon–intron organization and conserved synteny in order to deduce the evolution of the mAChR receptors. Our analyses verify the existence of two ancestral genes prior to the two vertebrate tetraploidizations (1R and 2R). After these events, one gene had duplicated, resulting in *CHRM2* and *CHRM4*; and the other had triplicated, forming the *CHRM1*, *CHRM3*, and *CHRM5* subfamily. All five genes are still present in all vertebrate groups investigated except the *CHRM1* gene, which has not been identified in some of the teleosts or in chicken or any other birds. Interestingly, the third tetraploidization (3R) that took place in the teleost predecessor resulted in duplicates of all five mAChR genes of which all 10 are present in zebrafish. One of the copies of the *CHRM2* and *CHRM3* genes and both *CHRM4* copies have gained introns in teleosts. Not a single separate (nontetraploidization) duplicate has been identified in any vertebrate species. These results clarify the evolution of the vertebrate mAChR family and reveal a doubled repertoire in zebrafish, inviting studies of gene neofunctionalization and subfunctionalization.

## Significance Statement

Despite their pivotal physiologic role, the evolution of the muscarinic acetylcholine receptors (mAChRs) has not yet been resolved. By investigating the genomes of a broad selection of vertebrate species and combining three different types of data, namely sequence-based phylogeny, conserved synteny, and intron organization, we have deduced the evolution of the mAChR genes in relation to the major vertebrate tetraploidizations (1R, 2R, and 3R). Our analyses show that all vertebrate mAChR gene duplications resulted from the tetraploidizations. Interestingly, following 3R, zebrafish doubled its gene number, resulting in the 10 mAChR genes present. By knowing how and when the mAChR genes arose, studies of receptor subtype specialization and possible neofunctionalization or subfunctionalization can follow.

## Introduction

The muscarinic acetylcholine receptors (mAChRs) are G-protein-coupled receptors (GPCRs) involved in a variety of CNS processes such as cognition, learning, and memory. They are also present in the peripheral nervous system and smooth muscle tissue. The mAChR family consists of five different receptor subtypes named M1–M5, which are encoded by the *CHRM1-5* genes. The structures of the muscarinic receptors follow the typical GPCR structure with the extracellular N terminus followed by seven transmembrane (TM) domains (TM domains 1–7), which are separated by three intracellular loops (ILs; 1–3), three extracellular loops (ELs; 1–3), and finally the intracellular C terminus. The orthosteric binding site for acetylcholine consists of a hydrophobic pocket formed by the side chains of TM domains 3–7. The crystal structures of the M2 and M3 receptors have been reported (Haga et al., 2012; Kruse et al., 2012), showing that the binding pocket contains identical amino acid residues in the M2 and M3 receptors (Haga et al., 2012; Kruse et al., 2012; Tautermann et al., 2013). In the study by Haga et al. (2012), 14 amino acid residues were found to form the antagonist binding sites and, following modeling of acetylcholine into the antagonist-binding pocket, 6 of these residues were suggested to bind acetylcholine. These six proposed acetylcholine-binding residues have also been reported to be conserved in *Drosophila melanogaster* (Collin et al., 2013). In the EL regions, the amino acid residues are less conserved, hence these have been targets for the design of drugs working as allosteric modulators (Christopoulos, 2002; Kruse et al., 2013, 2014). The M1, M3, and M5 receptors form one subfamily, coupling to G_q/11_, and the M2 and M4 receptors form one subfamily, coupling to G_i/o_. Hence, acetylcholine may give rise to different responses depending on which receptor subtype is present to initiate the signal transduction.

The mAChRs are widely expressed in the nervous system, the cardiovascular system, and the gastrointestinal tract, as well as elsewhere. In the peripheral nervous system, the mAChRs play a major role in the parasympathetic system stimulating smooth muscle contraction and glandular secretion as well as slowing the heart rate (Eglen, 2005). In the CNS of primates and rodents, the M1, M2, and M4 receptors are the most highly expressed mAChRs in the brain, but M3 and M5 are also present (Thiele, 2013; Lebois et al., 2018). Regarding mechanisms behind gene expression and similarities or dissimilarities among vertebrate species, little is known (Lebois et al., 2018). Each gene may have multiple promoters as has been demonstrated for CHRM2 (Krejci et al., 2004), and while some promoters are conserved across mammals, others differ and presumably contribute to anatomic or temporal differences in expression between species.

Although the muscarinic receptors have prominent roles in various nervous system functions, the evolution of the mAChR gene family has not yet been fully resolved. It is important to deduce evolutionary relationships to distinguish orthologs (species homologs), paralogs (gene duplicates), and ohnologs (gene duplicates resulting specifically from tetraploidization events), especially when studying species that belong to evolutionarily distant groups, for instance the commonly used experimental animals mouse/rat, chicken, and zebrafish. Furthermore, the time points of the gene duplication events are important for studies of evolutionary change between orthologs and paralogs as well as ohnologs. It is now well established that the vertebrate predecessor underwent two rounds of whole-genome duplication (i.e., tetraploidizations) before the radiation of jawed vertebrates (Nakatani et al., 2007; Putnam et al., 2008). These two events are usually referred to as 1R and 2R. In addition, the ancestor of the teleosts went through a third tetraploidization (3R) after the divergence from the most basal lineages of ray-finned fishes (Jaillon et al., 2004).

As the availability of high-quality genome assemblies is continuously increasing, it is now possible to perform a more extensive analysis of the evolution of the mAChR family. We have used an approach that combines amino acid sequence-based phylogeny and analyses of chromosomal locations for comparison of synteny and duplicated chromosome regions. We report here that the 1R and 2R genome-doubling events duplicated the two ancestral mAChR genes to the five genes that are present today in all tetrapods investigated except birds, where *CHRM1* has not been found. Furthermore, the teleost 3R event doubled the repertoire once more, resulting in the 10 genes present today in zebrafish, albeit some teleosts seem to lack both copies of *CHRM1*. This long-lived multiplicity invites further studies of the roles of each of the subtypes.

## Materials and Methods

### Species included in analysis and amino acid sequence retrieval

Species sequences included in the analysis of the mAChR family were the human (*Homo sapiens*; Hsa), mouse (*Mus musculus*; Mmu), opossum (*Monodelphis domestica*; Mdo), chicken (*Gallus gallus*; Gga), anole lizard (*Anolis carolinensis*; Aca), frog (*Xenopus tropicalis*; Xtr), coelacanth (*Latimeria chalumnae*; Lch), spotted gar (*Lepisosteus oculatus*; Loc), Japanese eel (*Anguilla japonica*; Aja), European eel (*Anguilla anguilla*; Aan), zebrafish (*Danio rerio*; Dre), stickleback (*Gasterosteus aculeatus*; Gac), medaka (*Oryzias latipes*; Ola), tunicates (*Ciona intestinalis*; Cin and *Ciona savigny*; Csa), and nematode (*Caenorhabditis elegans*; Cel). The amino acid sequences from the species listed were retrieved from the Ensembl genome browser (release 87; Zerbino et al., 2018; Ensembl Genome Browser, RRID:SCR_013367) or NCBI (NCBI; RRID:SCR_006472) databases. If sequences were not found in either of the databases, the sequence of a closely related species was used as a query sequence in a TBLASTN search (TBLASTN; RRID:SCR_011822). The Japanese and European eel genome assemblies are not annotated. Therefore, spotted gar mAChR gene sequences were used as templates to run a TBLASTN search and retrieve mAChR orthologs present in the Japanese eel; thereafter, Japanese eel sequences were used as templates in TBLASTN search for mAChR orthologs present in the European eel. Due to a high degree of sequence conservation between all mAChR genes in the Japanese eel and European eel, only the European eel sequences were included in sequence-based phylogenetic analysis as this species contained a more complete mAChR gene repertoire than the Japanese eel.

### Sequence alignments and phylogenetic analyses

**Figure 1.**
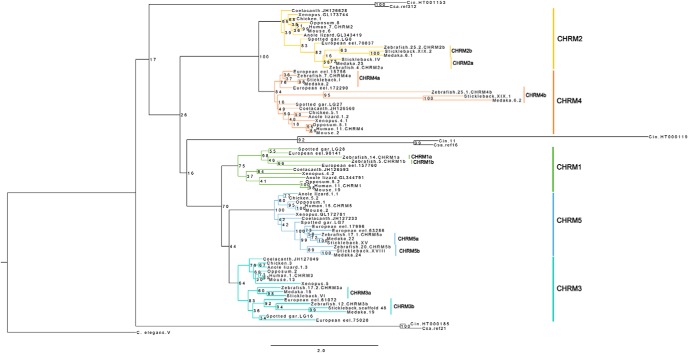
PhyML tree of the mAChR genes (*CHRM1*-*CHRM5*), rooted with *C. elegans*. The tree topology is supported by a nonparametric bootstrap analysis with 100 replicates. In the multiple sequence alignment that the PhyML tree is based on, the IL3 region was excluded as this region showed a low degree of sequence conservation. In the sequence names, the species is followed by the chromosome or genomic scaffold at which the gene is located (numbers or roman numerals). If several genes are located on the same chromosome or genomic scaffold, their order is indicated by an additional number. Cin, Ciona *intestinalis*; Csa, *Ciona savignyi*. All sequence details are listed in Fig. 1-4. The Jalview sequence alignment from which the PhyML tree was created is presented in Fig. 1-2. Fig. 1-1, and Figure 1-3, display the sequence alignment and PhyML tree of the complete sequences, including the IL3 region.

**Figure 2.**
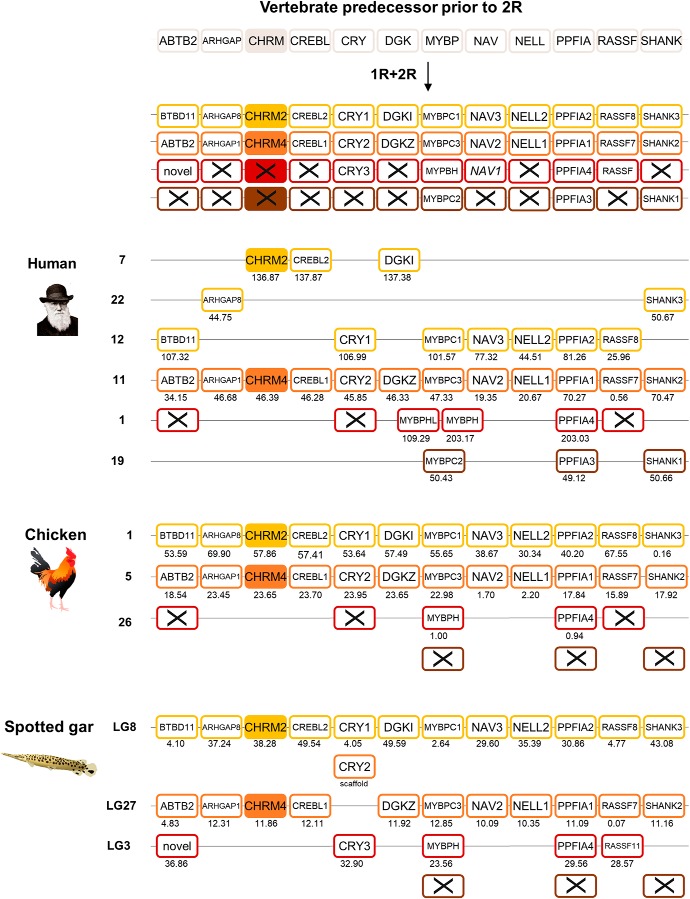
The evolutionary history and analysis of chromosomal regions and conserved synteny of the *CHRM2* and *CHRM4* genes and their neighboring gene families. The gene repertoire present in the vertebrate predecessor is displayed in the top panel, the duplication scheme further displays which orthologs were retained in the vertebrate ancestor following 1R and 2R, and finally the last three panels display the gene repertoire present in the human, chicken, and spotted gar. Crosses indicate gene loss or gene not (yet) identified. Dashed boxes represent incomplete sequences. Each paralogon member is presented in a separate color. Chicken and spotted gar illustrations are reused with permission from Daniel Ocampo Daza (source: www.egosumdaniel.se). The sequence details are listed in Fig. 2-3. The aLRT SH-like trees are displayed in Fig. 2-1, and the chromosomal regions and conserved synteny of the *CHRM2* and *CHRM4* genes and their neighboring gene families in zebrafish are displayed in Fig. 2-2.

**Figure 3.**
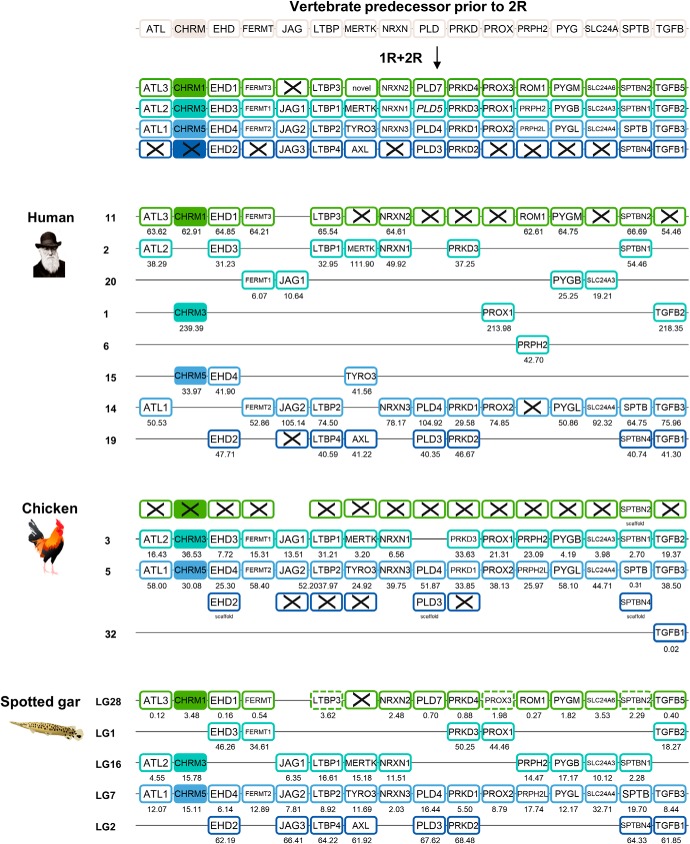
The evolutionary history and analysis of chromosomal regions and conserved synteny of the *CHRM1*, *CHRM3*, and *CHRM5* genes and their neighboring gene families. The gene repertoire present in the vertebrate predecessor is displayed in the top panel, the duplication scheme further displays which orthologs were retained in the vertebrate ancestor following 1R and 2R, and finally the last three panels display the gene repertoire present in the human, chicken, and spotted gar. Crosses indicate gene loss or gene not (yet) identified. Each paralogon member is presented in a separate color. Chicken and spotted gar illustrations are reused with permission from Daniel Ocampo Daza (source: www.egosumdaniel.se). The sequence details are listed in Fig. 3-3. The aLRT SH-like trees are displayed in Figure 3-1, and the chromosomal regions and conserved synteny of the *CHRM2* and *CHRM4* genes and their neighboring gene families in zebrafish are displayed in Figure 3-2.

**Figure 4.**
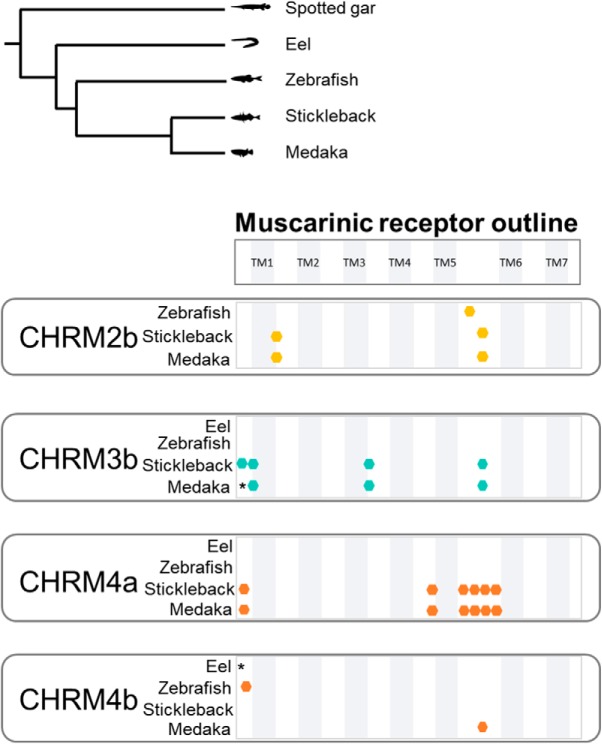
The localization of teleost-specific intron gains for the *CHRM2*b, *CHRM3*b, *CHRM4*a, and *CHRM4*b genes in the European eel, zebrafish, stickleback, and medaka. The top panel displays the relationship between the teleost species included in intron analysis, with the spotted gar as reference species followed by the mAChR outline and specific intron gains (indicated by colored hexagon) for the *CHRM2*b, *CHRM3*b, and *CHRM4*a. No *CHRM2b* sequence was identified in the European eel. Asterisk is present where an intron gain could not be confirmed. The sequence details are listed in Fig. 4-5. The Jalview sequence alignments of the teleost sequences analyzed are displayed for the *CHRM2*b, *CHRM3*b, *CHRM4*a, and *CHRM4*b genes in Fig. 4-1, Fig. 4-2, Fig. 4-3, and Fig. 4-4, respectively.

The retrieved amino acid sequences were aligned using Jalview 2.10.3b1, with Muscle default settings (Waterhouse et al., 2009; Jalview, RRID:SCR_006459). If the amino acid sequences were aligning poorly and the predictions appeared questionable, the genomic sequences were investigated and the sequences were manually edited, by comparing sequence homology and consensus donor and acceptor splice sites. Manual corrections were made where the alignment appeared shifted due to a low degree of conservation, such as the first part of the sequence or the IL3 loop region between TM5 and TM6. However, these adjustments were kept to a minimum. All sequence details are included in Fig. 1-4, and the full alignment is available in Fig. 1-1, and Fig. 1-2. A maximum likelihood analysis was performed using the PhyML 3.0 web server (PhyML; RRID:SCR_014629; Guindon et al., 2010). The optimal substitution model was selected by the “Automatic Model Selection by SMS” option, with the Akaike information criterion. Additional settings selected were as follows: BIONJ as starting tree, Subtree-pruning-regrafting (SPR) for tree improvement, no number of random starting tree, no fast likelihood methods, and finally perform bootstrapping with 100 replicates. The resulting tree was displayed in FigTree version 1.4.2 (FigTree, RRID:SCR_008515), rooted with *C. elegans*.

10.1523/ENEURO.0340-18.2018.f1-1 Figure 1-1 Jalview multiple sequence alignment of the mAChR amino acid sequences in the selected species repertoire. The three-letter abbreviation in the sequence names refers to species, and the numbers or roman numerals refer to the chromosome or genomic scaffold of gene location. If several genes are located on the same chromosome or genomic scaffold, their order is indicated by an additional number. Assigned sequence names and sequence information details are provided in Figure 1-4. Download Fig. 1-1, TXT file.

10.1523/ENEURO.0340-18.2018.f1-2 Figure 1-2 Jalview multiple sequence alignment of the mAChR genes as in Figure 1-1, but with the IL3 region excluded from alignment due to the low degree of sequence conservation. The three-letter abbreviation in the sequence names refers to species, and the numbers or roman numerals refer to the chromosome or genomic scaffold of gene location. If several genes are located on the same chromosome or genomic scaffold, their order is indicated by an additional number. Assigned sequence names and sequence information details are provided in Fig. 1-4. Download Fig. 1-2, TXT file.

10.1523/ENEURO.0340-18.2018.f1-3 Figure 1-3 PhyML tree of the mAChR genes (*CHRM1*–*CHRM5*), rooted with *C. elegans*. The tree topology is supported by a nonparametric bootstrap analysis with 100 replicates. This tree is based on the complete multiple sequence alignment (i.e. the IL3 region is also included). In the sequence names, the species is followed by the chromosome or genomic scaffold at which the gene is located (numbers or roman numerals). If several genes are located on the same chromosome or genomic scaffold, their order is indicated by an additional number. Cin, *Ciona intestinalis*; Csa, *Ciona savigny*. Download Fig. 1-3, TIF file.

10.1523/ENEURO.0340-18.2018.f1-4 Figure 1-4 Information about the mAChR amino acid sequences retrieved and included in the analysis. First, the genome assembly versions used are stated, followed by information about the mAChR gene sequences included in the analysis in the following order: species, HGNC/ZFIN/Flybase symbol name, chromosome or genomic scaffold position, orientation, Ensembl ID or NCBI accession number, assigned sequence name in alignment, assigned sequence name in PhyML tree, and additional comments regarding sequence update date on NCBI or whether there have been manual edits of the original Ensembl or NCBI sequence. The order of sequences is organized according to orthology. Download Fig. 1-4, DOCX file.

### Conserved synteny and paralogon analysis

For synteny and paralogon analyses, the neighboring regions of the mAChR genes were investigated in human, chicken, and spotted gar. Lists of gene families in the genomic regions 10 Mb upstream and 10 Mb downstream of the *CHRM2/CHRM4* and *CHRM1/CHRM3/CHRM5* genes, respectively, in the spotted gar genome were retrieved using the Biomart function in Ensembl version 83 (Ensembl Genome Browser; RRID:SCR_013367). For the *CHRM2/CHRM4* genes, lists of gene families were also retrieved in a similar manner in the chicken genome, due to the small number of neighboring gene families in the genomic regions in the spotted gar. Thereafter, families containing two members or more were phylogenetically analyzed by retrieving the amino acid sequences for human, chicken, coelacanth, spotted gar, and zebrafish. The region surrounding the *CHRM1*, *CHRM3*, and *CHRM5* genes contained a higher number of families; therefore, families with at least three gene family members in the spotted gar were analyzed. As outgroups tunicates, amphioxus, drosophila, *C. elegans* and in some cases other human gene sequences were included. aLRT SH-like trees were constructed using the PhyML 3.0 web server (PhyML; RRID:SCR_014629; Guindon et al., 2010) to verify the sequence orthology. To apply the most optimal selection model the “Automatic model selection by SMS” model was selected, with Akaike information criterion. SPR was used as tree improvement method. If the members of a family showed unclear topology and/or weak node support and/or if the family showed a high degree of conservation and/or lack of outgroups and/or massive expansions due to local duplications, it was excluded from the analysis. The relatively low number of gene families in the regions surrounding the *CHRM2* and *CHRM4* genes resulted in lack of information of the fourth chromosome and its gene family members in the spotted gar. Therefore, synteny figures of the current neighboring gene repertoire in the zebrafish were prepared and included, for the paralogon structure in the ray-finned fishes. Based on the results from the phylogeny and synteny analyses, some genes appeared incorrectly named or were lacking names; therefore, genes were renamed or named according to the “ZFIN Zebrafish Nomenclature Conventions” (ZFIN; RRID:SCR_002560), and the proposed gene names were submitted to ZFIN. The details of the gene family sequences included in the analysis are provided in Fig. 2-3, for the *CHRM2* and *CHRM4* paralogon and in Fig. 3-3, for the *CHRM1*, *CHRM3*, and *CHRM5* paralogon and the aLRT SH-like trees are included in Fig. 2-1, and 3-1, respectively.

### Intron position analysis in teleosts

To analyze specific intron gains in the *CHRM2b*, *CHRM3b*, *CHRM4a*, and *CHRM4b* teleost genes, additional teleost species included in the analysis were the Amazon molly (*Poecilia formosa*; Pfo) and fugu (*Takifugu rubripes*; Tru). Sequences were analyzed and aligned as described above. Intron positions were determined by manual investigation of amino acid and nucleotide sequences. Transmembrane domain regions were predicted by consulting the TMHMM Server version 2.0 (TMHMM Server; RRID:SCR_014935). For comparative analyses and confirmation of intron positions, the Japanese eel was also analyzed.

## Results

### Two ancestral mAChR genes expanded to five following 1R and 2R

The multiple sequence alignment analysis of the mAChR genes confirmed a generally high degree of overall sequence identity both across receptor subtypes and across species. The degree of identity for the seven TM regions between one of the most slowly evolving vertebrate model species, spotted gar, and human is ∼83% for *CHRM1*, 87% for *CHRM3*, and 90% for *CHRM5*. In the other subfamily, the identity is even higher with 96% for *CHRM2* and 95% for *CHRM4*, displayed by Jalview 2.10.3b pairwise alignment. Overall, *CHRM2* displays the highest degree of conservation, whereas the *CHRM1* displays the lowest. The *CHRM2/CHRM4* subfamily displays a higher degree of conservation than the *CHRM1/CHRM3/CHRM5* subfamily. This is also confirmed in human–chicken ortholog comparisons and human paralog comparisons. However, when including the complete amino acid sequences in the pairwise alignment, the percentage of identity drops considerably. For instance, the well conserved *CHRM2* subtype decreases from 96% to 75% identity when including the complete sequence. One reason for this is that IL3 located between TM5 and TM6 is highly variable between receptor subtypes as well as between species for each subtype (Fig. 1-1). This region is involved in interactions with G-proteins and other cytoplasmic components and is a potential target for regulatory phosphorylations. If the most variable part of the IL3 region is excluded from the pairwise alignment, the following identities are found: 70% for *CHRM1*; 76% for *CHRM3*; 80% for *CHRM5*; 87% for *CHRM2*; and 86*%* for *CHRM4*. Hence, *CHRM2* increases from 75% to 87%. Due to the high variability in the IL3 region, an alignment excluding this region was prepared (Fig. 1-2) that served as the basis for the phylogenetic analyses. Further, the six amino acid residues proposed to be involved in acetylcholine binding by (Collin et al., 2013) were conserved across all vertebrate sequences included in this study.

The phylogenetic maximum likelihood (PhyML) tree of the predicted mAChR proteins is displayed in Figure 1. The tree is rooted with a sequence from the nematode *C. elegans*. After the split between protostomes and deuterostomes, the primordial gene was duplicated in the deuterostome lineage to form the two ancestral mAChR genes present in the vertebrate predecessor, later to form the two mAChR subfamilies. The closest relatives of these two vertebrate subfamilies are two groups of tunicate sequences. The ancestor of the *CHRM2/CHRM4* subfamily duplicated in the 1R-2R tetraploidizations, as shown by paralogon comparisons described below and also supported by the species distribution, resulting in the *CHRM2* and *CHRM4* genes (Fig. 1). The *CHRM2* and *CHRM4* genes are present in all vertebrates investigated. Furthermore, duplicates of the *CHRM2* and *CHRM4* genes are present in zebrafish, medaka, and stickleback. A duplicate of the *CHRM4* gene is also present in the European eel, but only one *CHRM2* gene has been found in this species. The ancestor of the *CHRM1*/*CHRM3*/*CHRM5* subfamily triplicated in 1R-2R giving rise to the *CHRM1*, *CHRM3*, and *CHRM5* genes (Fig. 1). The *CHRM3* and *CHRM5* genes are present in all vertebrates investigated, with duplicates in the teleosts. However, the *CHRM1* gene shows a slightly different species distribution; the gene has not been identified in the chicken, and we were unable to find it in any of the bird genomes. The gene is also missing in the medaka and stickleback genome assemblies, but it is present in European eel and zebrafish, and it has also retained duplicates in both species. Notably, the PhyML analysis shows that, despite exclusion of the IL3 loop with its low sequence conservation, some mAChR family genes have evolved at much higher rates, particularly in some of the teleosts. The *CHRM1* orthologs also appear to have evolved at a higher rate than the other four subtypes, as shown by the long branches in the phylogenetic tree (Fig. 1).

The *CHRM1*/*CHRM3*/*CHRM5* subfamily has a few tunicate sequences as its closest relatives (Fig. 1), whereas the *CHRM2/CHRM4* subfamily does not. Instead, there are two groups of tunicate sequences present basally to both of the vertebrate subfamilies. However, the bootstrap values show that there is weak node support for the positioning of the tunicate sequences in the PhyML tree, and some of them have also evolved very fast, as shown by their long branches; hence, their positioning in the PhyML may not mirror the actual phylogeny.

The positioning of the tunicates basally to the *CHRM1*/*CHRM3*/*CHRM5* subfamily shows that the expansion of this subfamily occurred after the divergence of the vertebrates and the invertebrate chordates (here represented by tunicates), a period that coincides with the timing of the 1R and 2R events. This is further supported by the species distribution of these three subtypes. Although a tunicate group is missing for the *CHRM2/CHRM4* subfamily, the timing of the duplication events, as well as the species distribution of the two subtypes, coincides with the duplication events in the *CHRM1*/*CHRM3*/*CHRM5* subfamily, supporting the 1R and 2R expansion hypothesis also for this subfamily. Hence, from this phylogenetic analysis we conclude that the mAChR family most likely expanded from two ancestral members present before 1R and 2R, to five members present following the vertebrate tetraploidizations. Additionally, duplicates found in the group of teleosts coincide with the timing of the teleost-specific tetraploidization, hence suggesting that those gene duplicates are paralogs resulting from 3R and can thus be called ohnologs (see below). The PhyML tree resulting from the analysis of the complete multiple sequence alignment, also including the IL3 region, is shown in Fig. 1-3. Details about all sequences included in the analysis are listed in Fig. 1-4.

### Analysis of synteny blocks confirms expansion of the mAChR family by 1R and 2R

To explore the hypothesis that two ancestral mAChR genes expanded to five as a result of the basal vertebrate tetraploidizations, analyses of the mAChR neighboring genomic regions were conducted. If the members of each of the two mAChR subfamilies are located in chromosomal regions that also contain representatives from several other gene families, this would strongly indicate that a large block of genes, or even a chromosomal region or an entire chromosome, had been duplicated. The related genes resulting from these events are named ohnologs, as described in the Introduction. On the other hand, if the members of an mAChR subfamily are in completely different chromosomal neighborhoods, this would indicate independent duplications of an mAChR gene and insertion into unrelated chromosomal regions. Related chromosomal regions that arose as a result of the 1R and 2R tetraploidizations (or any other tetraploidization event) are referred to as comprising a paralogon (Coulier et al., 2000). Thus, the vertebrate ancestral genome consisted of paralogons with quartets of related regions, whereas the teleost ancestor had paralogons with eight related members as a result of 3R. In extant species, the paralogons have often secondarily lost some of the ohnologs. Our phylogenetic analysis of the mAChR family showed that the expansion of the mAChR gene family coincides with the time period of the tetraploidizations. We therefore analyzed the neighboring gene families to see whether these too expanded during this time period and also whether they have representatives in the other chromosomal regions of the same paralogon.

The genomic regions surrounding the *CHRM2*/*CHRM4* genes in the spotted gar were retrieved, and the gene families with at least two members present were analyzed. Following the exclusion criteria at the preliminary analysis stage stated in the Materials and Methods, five gene families were included in the final analysis, namely *ARHGAP*, *NAV*, *NELL*, *PPFIA*, and *SHANK*. However, due to the low number of gene families found in spotted gar, the genomic regions surrounding the *CHRM2*/*CHRM4* genes were investigated in the chicken and six additional gene families were identified. The reason why they were not found in the neighboring regions in spotted gar could be chromosomal rearrangements. The additional neighboring families are *ABTB2*, *CREBL*, *CRY* (for sequence details and phylogenetic analysis, see Haug et al., 2015), *DGK*, *MYBP*, and *RASSF*. Information about the neighboring gene families is included in Fig. 2-3, and their phylogenetic trees [aLRT (approximate likelihood-ratio test) SH (Shimodaira–Hasegawa)-like trees] are included in Fig. 2-1. From the phylogenetic analyses, orthologous and paralogous genes were determined, and the chromosomal locations of the neighboring gene families in human, chicken, and spotted gar are presented in Figure 2. In humans, the first member of this paralogon (Fig. 2, yellow) consists of regions located on three separate chromosomes (chromosomes 7, 22, and 12). In chicken and spotted gar, the orthologs are located on a single chromosome (chromosome 1 in the chicken and LG8 in spotted gar). This strongly suggests that this paralogon member in the human genome most likely was split by chromosomal translocations. The second paralogon member (Fig. 2, orange) is located on a single chromosome in human and chicken, and with one exception also in spotted gar. Also the third paralogon member is restricted to a single chromosome for these gene families in all three species. Note that the *MYBPHL* and *MYBPH* genes in human are a result of a more recent local duplication (Fig. 2, red). This paralogon member has undergone more ohnolog losses than the other two members. The fourth paralogon member (Fig. 2, brown) seems to have undergone even more ohnolog losses and is only present in the human genome, with members from the *MYBP*, *PPFIA*, and *SHANK* families (on chromosome 19).

10.1523/ENEURO.0340-18.2018.f2-1 Figure 2-1 Phylogenetic aLRT SH-like tree of the neighboring gene families included in analysis of the *CHRM2* and *CHRM4* genomic regions. There is one neighboring gene family per page in alphabetical order. Orthologs are color coded according to paralogon member. Gray color means that those orthologs are not included in paralogon analysis due to exclusion based on results from the phylogenetic analysis, or that there is no human, chicken, or spotted gar sequence present. Assigned sequence names and sequence information details are provided in Fig. 2-3. Download Fig. 2-1, TIF file.

10.1523/ENEURO.0340-18.2018.f2-2 Figure 2-2 Analysis of chromosomal positions of the *CHRM2* and *CHRM4* genes and their neighboring gene families included in the paralogon analysis in Figure 2 in the zebrafish. Each paralogon member is presented in a separate color. The zebrafish illustration is reused with permission from Daniel Ocampo Daza (source: www.egosumdaniel.se). Download Fig. 2-2, TIF file.

10.1523/ENEURO.0340-18.2018.f2-3 Figure 2-3 Information about the neighboring gene family sequences included in the analysis of the genomic regions surrounding the *CHRM2* and *CHRM4* genes. First, the genome assembly versions used are stated, followed by information about the neighbor gene families included in the analysis in the following order: species, HGNC/ZFIN/Flybase symbol name, chromosome or genomic scaffold position, Ensembl ID or NCBI accession number, assigned sequence name, and additional comments regarding the sequence update date on NCBI or whether there have been manual edits of the original Ensembl or NCBI sequence. Download Fig. 2-3, DOCX file.

To investigate whether this paralogon member is present in other species, the neighboring gene family repertoire and chromosomal locations were investigated in zebrafish, presented in Fig. 2-2. The fourth paralogon member was indeed found to be present in zebrafish, represented by *MYBP*, *PPFIA*, and *SHANK* ohnologs (on chromosomes 3 and 24). However, it appears that additional translocations have occurred in zebrafish, most likely following the 3R event. These genes are located in a paralogon that has been studied previously, where the focus was on a region containing numerous other gene families including the visual opsins, transducin alpha subunits and oxytocin/vasopressin receptors (Lagman et al., 2013). Therefore, although the third and fourth members of this region have undergone several ohnolog losses, it is nevertheless clear that the *CHRM2* and *CHRM4* genes are located in a paralogon that arose in the basal vertebrate tetraploidizations.

The *CHRM1*/*CHRM3*/*CHRM5* subfamily arose from a separate ancestor gene. Our investigation of the paralogon hypothesis was initiated by retrieving the genomic regions surrounding the *CHRM1*/*CHRM3*/*CHRM5* genes in spotted gar. These contained a higher number of gene families than the regions surrounding the *CHRM2*/*CHRM4* genes and therefore the analysis was restricted to gene families with three or four members. Following the exclusion criteria stated in the Materials and Methods, 15 neighboring gene families were included in the final analysis, namely *ATL*, *EHD*, *FERMT*, *JAG*, *LTBP*, *MERTK*, *NRXN*, *PLD*, *PRKD*, *PROX*, *PRPH2*, *PYG*, *SLC24A*, *SPTB*, and *TGFB*. Information about the neighboring gene families is included in Fig. 3-3, and the phylogenetic trees (aLRT SH like) are included in Figure 3-1. Based on the phylogenetic analyses, the chromosomal locations of the gene family members are shown for human, chicken, and spotted gar in Figure 3.

10.1523/ENEURO.0340-18.2018.f3-1 Figure 3-1 aLRT SH-like tree of the neighboring gene families included in analysis of the *CHRM1*, *CHRM3*, and *CHRM5* genomic regions. There is one neighboring gene family per page in alphabetical order. Orthologs are color coded according to paralogon member. Gray color means that those orthologs are not included in paralogon analysis due to exclusion based on results from the phylogenetic analysis, or that there is no human, chicken, or spotted gar sequence present. Two trees are included for the following gene families: LTBP, PROX, and SPTB, where the first tree includes all sequences and the second one contains all sequences except the Loc.LG28, due to its shortness in length. There are two trees included also for the PLD gene family, the first tree is based on complete alignment, and the second tree is based on an alignment where the first part of alignment is excluded due to a very low degree of sequence conservation and highly variable sequence lengths. Assigned sequence names and sequence information details are provided in Fig. 3-3. Download Fig. 3-1, TIF file.

10.1523/ENEURO.0340-18.2018.f3-2 Figure 3-2 Analysis of chromosomal positions of the *CHRM1*, *CHRM3*, and *CHRM5* genes and their neighboring gene families included in the paralogon analysis in Figure 3 in the zebrafish. Each paralogon member is presented in a separate color. The zebrafish illustration is reused with permission from Daniel Ocampo Daza (source: www.egosumdaniel.se). Download Fig. 3-2, TIF file.

10.1523/ENEURO.0340-18.2018.f3-3 Figure 3-3 Information about the neighboring gene family sequences included in the analysis of the genomic regions surrounding the *CHRM1*, *CHRM3*, and *CHRM5* genes. First, the genome assembly versions used are stated, followed by information about the neighbor gene families included in the analysis in the following order: species, HGNC/ZFIN/Flybase symbol name, chromosome or genomic scaffold position, Ensembl ID or NCBI accession number, assigned sequence name, and additional comments regarding sequence update date on NCBI or whether there have been manual edits of the original Ensembl or NCBI sequence. Download Fig. 3-3, DOCX file.

The first paralogon member (Fig. 3, green) is located on a single chromosome in human and spotted gar (chromosome 11 and LG28, respectively). Three of the genes located on LG28 (*LTBP3*, *PROX*, and *SPTBN*2; Fig. 3, dashed boxes) were incomplete in the genome database and contain <50% of the sequence. As this might impact the topology in the aLRT SH-like trees, trees were also generated where these sequences were excluded; nevertheless, the results remained the same (Fig. 3-1). None of the genes of the first paralogon member are present in chicken, except the *SPTB* family member *SPTBN2* (located on a scaffold). As mentioned previously, the *CHRM1* gene is absent in the chicken, as well as in other birds. The second and third paralogon members in human are located on three and two different chromosomes, respectively (Fig. 3, turquoise and light blue). The second member is located on two different chromosomes in spotted gar (LG1 and LG16). In chicken, both the second and the third paralogon members are located on a single chromosome. Finally, the fourth paralogon member is located on chromosome 19 in human and on LG2 in spotted gar (Fig. 3, dark blue). In chicken, the fourth member could not be identified for four of the gene families, and three ohnologs are located on scaffolds (*EHD2, PLD3*, and *SPTBN4*). However, one member of the TGFB family, the *TGFB1* gene, is located on chromosome 32, which is a very short chromosome in the chicken, consisting of only ∼78 kb.

The synteny analysis in human, chicken, and spotted gar shows that these genomic regions have undergone a number of rearrangements such as translocations, and several ohnologs could not be identified. This is further seen when analyzing the *CHRM1*/*CHRM3*/*CHRM5* neighboring gene repertoire and chromosomal locations in zebrafish, presented in Fig. 3-2. It appears that a number of translocations have occurred in zebrafish, as for instance the first paralogon member (Fig. 3-2, green) is located on five different chromosomes, and the second member is located on no less than seven different chromosomes (Fig. 3-2, turquoise). This paralogon too has been studied in detail in a previous study focusing on somatostatin receptor genes including multiple neighboring gene families. These chromosomal regions were found to be related through the 1R, 2R, and 3R events and thereby comprise a paralogon (Ocampo Daza et al., 2012).

Thus, the analysis of conserved synteny and paralogons of the two mAChR subfamilies and their neighboring gene families confirms our hypothesis based on the phylogenetic analyses that the mAChR gene family expanded by the 1R and 2R tetraploidizations from two ancestral genes into the five members present today in most tetrapods and some basally diverging vertebrates, and 8-10 members in teleosts, forming two distinct subfamilies belonging to two separate paralogons.

### Teleost-specific intron gains in the *CHRM2*b, *CHRM3*b, *CHRM4*a, and *CHRM4*b genes

The amino acid sequence analyses and alignments revealed that some of the teleost sequences contained annotated introns in the genome assemblies, although the mAChR genes in general have been said to lack introns in the coding region (Bonner et al., 1987, 1988; Peralta et al., 1987; Seo et al., 2009). To investigate whether these introns were indeed teleost-specific gains, or whether they could be the results of gene annotation or sequencing difficulties, an extended sequence repertoire from teleosts was analyzed. In the sequence analyses, it was found that the *CHRM2*b, *CHRM3*b, *CHRM4*a, and *CHRM4*b genes have independently gained at least one intron in the proximity of the region encoding TM1 and at least one intron in the region encoding the IL between TM5 and TM6, in at least one of the teleost species investigated (Fig. 4). In *CHRM2*b, stickleback and medaka have gained one intron in the end of the TM1 domain (Fig. 4). This intron gain is supported by analysis of fugu and Amazon molly *CHRM2b* sequences, as they too contain this intron (Fig. 4-1). One intron is also present in the IL3 domain, located between TM5 and TM6, in zebrafish, stickleback, and medaka. However, it seems that this intron is not the same in the three teleost species. Rather, one intron seems to have been gained in zebrafish and a separate intron was gained in the ancestor of stickleback and medaka (Fig. 4). No *CHRM2b* gene could be identified in European eel (or in the Japanese eel), and therefore it was not possible to determine the exact time point when this intron was gained in zebrafish. The intron present in stickleback and medaka was also found in fugu and Amazon molly (Extended Data Fig. 4-1).

10.1523/ENEURO.0340-18.2018.f4-1 Figure 4-1 Jalview sequence alignment of the teleost species sequences included in the analysis of teleost-specific intron gains in the *CHRM2b*, *CHRM3b*, *CHRM4a*, and *CHRM4b* genes. The top line displays predicted protein domains. Arrow indicates intron gain. Square indicates phase 1 or phase 2 splicing, and line indicates phase 0 splice. Introns with certain positions are marked in red or blue. Intron locations with uncertain positions, due to sequence quality or species inconsistencies, are marked in gray. The spotted gar and stickleback illustrations are reused with permission from Milton Tan (https://creativecommons.org/licenses/by-nc-sa/3.0/). Download Fig. 4-1, TIF file.

10.1523/ENEURO.0340-18.2018.f4-2 Figure 4-2 Supplementary Figure 4-2. Download Fig. 4-2, TIF file.

10.1523/ENEURO.0340-18.2018.f4-3 Figure 4-3 Supplementary Figure 4-3. Download Fig. 4-3, TIF file.

10.1523/ENEURO.0340-18.2018.f4-4 Figure 4-4 Supplementary Figure 4-4. Download Fig. 4-4, TIF file.

**Figure 5.**
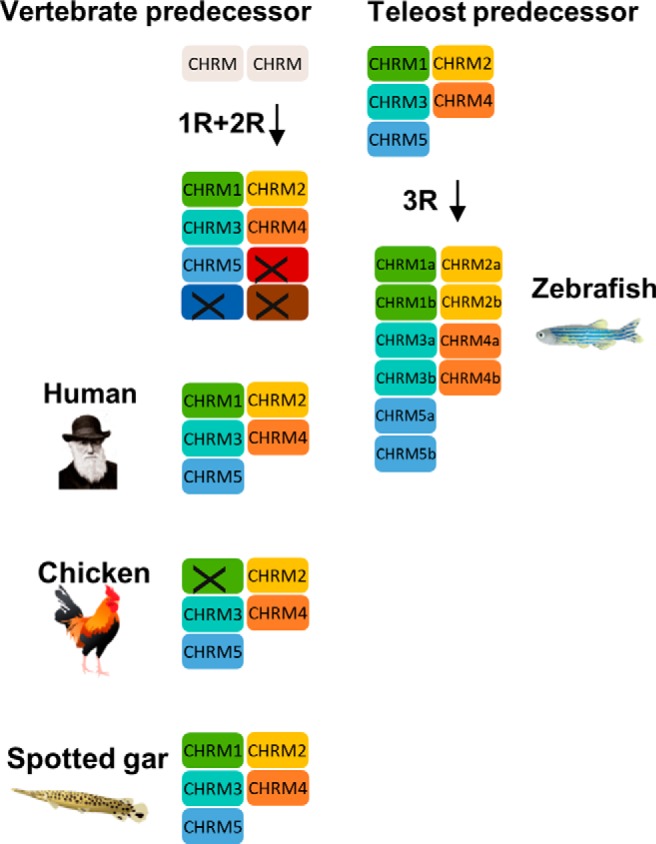
Duplication scheme of the mAChR genes following 1R, 2R, and 3R. Two mAChR genes present in the vertebrate predecessor expanded to five mAChR genes following 1R and 2R. All genes were retained in the human, chicken, and spotted gar except for the *CHRM1* gene in the chicken. In the teleost predecessor, 5 mAChR genes expanded to 10 genes following 3R, of which all duplicates are retained in the zebrafish. Chicken, spotted gar, and zebrafish illustrations are reused with permission from Daniel Ocampo Daza (source: www.egosumdaniel.se).

10.1523/ENEURO.0340-18.2018.f4-5 Figure 4-5 Information about the teleost mAChR amino acid sequences included in the analysis of teleost-specific intron gains. First, the genome assembly versions used are stated, followed by information about the teleost mAChR sequences included in the analysis in the following order: species, HGNC/ZFIN/Flybase symbol name, chromosome or genomic scaffold position, Ensembl ID or NCBI accession number, assigned sequence name, and additional comments regarding sequence update date on NCBI or whether there have been manual edits of the original Ensembl or NCBI sequence. Download Fig. 4-5, DOCX file.

The *CHRM3b* gene has gained one intron located in the beginning of the region encoding TM1 in medaka and stickleback and one additional intron in the N-terminal region in stickleback (Fig. 4). However, the first exon could not be identified in stickleback, although the presence of an intron at this position is supported by an identical intron found in fugu, which is most likely present also in Amazon molly (Extended Data Fig. 4-2). No exons upstream of the intron in the beginning of TM1 in medaka *CHRM3b* could be identified, and therefore it was not possible to confirm the presence of additional introns in medaka (Extended Data Fig. 4-2).

The *CHRM4*a gene has gained the largest number of introns. It has gained one intron in the N-terminal region in the ancestor of medaka and stickleback (Fig. 4). The first exon could not be found in stickleback, but there is a suitable consensus splice acceptor site present at the position corresponding to the intron present in medaka. This possible splice site is also present in fugu and Amazon molly (Extended Data Fig.4-3). Another intron is present in the region encoding EL2, just before TM5, in stickleback and medaka (Fig. 4), as well as in fugu and Amazon molly (Extended Data Fig. 4-3). Finally, four introns have been gained in the large region encoding IL3 of *CHRM4a* in the ancestor of stickleback and medaka (Fig. 4) and is also present in fugu and Amazon molly (Extended Data Fig. 4-3). Among these four genes, *CHRM4*b is the one that has gained the lowest number of introns. There is one intron present in the N-terminal region in zebrafish (Fig. 4). This intron is not found in any of the other teleosts analyzed. However, there is one possible intron present in European eel, but before this position there is also a methionine present that could act as translation initiator, meaning that this intron may not be present in European eel (Extended Data Fig. 4-4). However, the methionine is not present in Japanese eel, which implies that there should be an intron present at this position. Due to these inconsistencies between European eel and Japanese eel, it is not possible to conclude whether or not there is an intron present in the N-terminal region in these species. There is also one intron gained in IL3 in *CHRM4*b in medaka (Fig. 4), an intron that is also present in Amazon molly (Fig. 4). Information about the teleost sequences included in this analysis is provided in Extended Data Fig. 4-5.

## Discussion

Our analyses of the mAChR gene family are based on the following three types of information: sequence-based phylogenetic analysis; synteny and paralogon analysis; as well as analysis of teleost-specific intron gains. The combined results of these analyses show that the mAChR family expanded from two ancestral genes present in the vertebrate predecessor to five mAChR genes in an early vertebrate ancestor, as a result of the 1R and 2R tetraploidization events (Fig. 5). All five members could be identified in the vertebrate classes of mammals, birds, reptiles, amphibians, and bony fishes, with the exception of the *CHRM1* gene, which, surprisingly, has not been identified in chicken or any other bird. It remains possible that the gene exists and is located on a microchromosome, because it is well known that these are under-represented in the genome sequencing projects, probably partly due to their extremely high GC content (Burt, 2002; Zhang et al., 2014).

We also identified all five mAChR genes in a ray-finned fish, the spotted gar, which represents an early branch in the ray-finned fish tree. The teleosts, which constitute 99.9% of all ray-finned fishes, are descendants of a lineage that underwent a third tetraploidization, and for the mAChR family all 10 genes deriving from this event have been retained in zebrafish. The phylogenetic analysis shows that the teleost-specific tetraploidization 3R resulted in duplicates of all mAChR genes in zebrafish, resulting in a total of 10 mAChR genes (Fig. 5). A doubled mAChR repertoire in zebrafish has been reported before (Seo et al., 2009; Nuckels et al., 2011); however, neither of the previous studies tied it to the 3R tetraploidization. Here we can explain all of these duplications by a single genomic event (and previous gene duplications by the 1R/2R events). The European eel has retained nine of the genes, lacking one of the *CHRM2* duplicates, whereas medaka and stickleback are lacking the *CHRM1* gene. Notably, no nontetraploidization duplicates of any of the mAChR genes was found in any of the vertebrate species analyzed.

The *CHRM2/CHRM4* subfamily contains two ohnologs resulting from the 1R-2R tetraploidizations. It is unclear whether these two genes arose in 1R, and both of their duplicates after 2R were lost, or whether one copy was lost after 1R and the other was duplicated in 2R. The *CHRM1/CHRM3/CHRM5* subfamily contains three of the ohnologs resulting from the two tetraploidizations. This means that the ancestral gene duplicated once in 1R, and then both copies duplicated once more in 2R, after which one ohnolog was lost. As the two tetraploidizations were probably very close in time to one another, it is difficult to say which two may be the results of the 2R tetraploidization.

Thus, the repertoire of mAChR genes is quite consistent across vertebrates. This is presumably a reflection of unique functional roles for each of the gene products. The only gene that deviates from this pattern is *CHRM1.* Not only does it seem to be missing in birds, it has also not been identified in a few teleosts, namely stickleback and medaka. The pairwise alignments of the mAChR sequences showed that the *CHRM1* gene displayed the lowest degree of sequence identity; hence, it is more likely that this gene could be lost, or that the low degree of conservation has impeded its identification in the species where it has so far not been found. In fact, the whole paralogon member is missing in chicken, but there are still two possible reasons for this: either this whole chromosomal region was lost; or the whole region ended up on a microchromosome that is as yet unsequenced. In fact, one gene in this paralogon member (*SPTBN2*) has been identified, it is on a scaffold, perhaps indicating that additional members may be possible to identify.

Interestingly, a more thorough analysis of the amino acid sequences and especially the IL3 region, which has a low degree of sequence conservation, revealed that there has been a number of teleost specific introns gained in the coding regions of the *CHRM2*b, *CHRM3*b, *CHRM4*a, and *CHRM4*b genes. A previous study by Seo et al. (2009), which focused on a subset of mAChRs and smooth muscle contraction responses in Nile tilapia reported that all five mAChR genes present had retained paralogs in zebrafish, resulting in 10 mAChR genes present in total. The study by Seo et al. (2009) also reported that no introns were present in the mAChR genes studied. However, with the increased availability of data, and especially genome assemblies, our analyses have identified and verified a number of intron gains in several teleosts (Fig. 4, Fig. 4-1, Fig. 4-2, Fig. 4-3, and Fig. 4-4). Our findings are supported by those of a previous study reporting that the intron turnover in Actinopterygii is high, especially in the stickleback and zebrafish (Venkatesh et al., 2014; Ravi and Venkatesh, 2018). However, to our knowledge intron gains have not been previously reported for the mAChR genes in teleosts. The intron gains have taken place in teleost genes that evolve faster than their orthologs in other lineages, especially *CHRM2b*, *CHRM3b*, and *CHRM4b* (Fig. 1 and Fig. 1-3).

Duplication by chromosome doubling means by default that the two ohnologs deriving from the same mother gene must initially have had identical gene regulatory elements and hence identical expression patterns, anatomically, temporally, and quantitatively. This may initially result in an additive effect on the level of gene expression, unless compensatory mechanisms are at play. Subsequently, it is possible that either or both of the ohnologs may begin to accumulate mutations, either regulatory or structural, that will alter the functions of the gene. One possibility is that one of the ohnologs maintains the functions of the mother gene, leaving the other free to take on other roles (i.e., neofunctionalization). This was a possibility favored by Ohno (1970). Alternatively, the two ohnologs may lose regulatory elements such that they subdivide the functions of the mother gene between them in a process called subfunctionalization. As the mAChR genes were doubled in zebrafish and all duplicates are retained, it would be interesting to study possible neofunctionalizations or subfunctionalizations of these genes, initially by investigating gene expression patterns in anatomic mapping studies.

## Synthesis

Reviewing Editor: Menno Witter, Norwegian University of Science and Technology Decisions are customarily a result of the Reviewing Editor and the peer reviewers coming together and discussing their recommendations until a consensus is reached. When revisions are invited, a fact-based synthesis statement explaining their decision and outlining what is needed to prepare a revision will be listed below. The following reviewer(s) agreed to reveal their identity: NONE.

Reviewer 1:

The manuscript describes a study in which amino acid sequence based phylogenetic analysis, comparisons of exon-intron organization and gene location, and teleost specific intron gains are used to determine the evolution of the mAChR. The analyses leads the authors to conclude that there were two genes in the ancestral state prior to two duplicating events. The first duplication of one gene resulted in CHRM2 and CHRM4, while the second gene triplicated, giving rise to CHRM1, CHRM3 and CHRM5. Another duplication event in teleosts gave rise to a total of 10 genes. The authors provide reasonably convincing genetic analyses to support their conclusions. The manuscript is well-written and the analyses appears to be done appropriately. I have a few minor concerns that I ask the authors to address:

1. I encourage the authors to discuss why the resolution of the AChR gene family is important to resolve. The authors state that “the evolution of the mAChR gene family has not been fully resolved”, but do not elaborate on why this is important. Please clarify on the discussion why this work was important to do.

2. In my opinion the duplication scheme (Figure 5) is not absolutely necessary and the manuscript will fare just as well without it. But I leave this up to the authors to decide.

Reviewer 2:

The present manuscript presents a study on the evolution of the muscarinic cholinergic receptor genes of vertebrates by way of an in silico analysis. This is done by comparing sequences, exon-intron organization and conserved sinteny in a broad selection of species, including invertebrate chordates (tunicates) as well as different vertebrates, with representative species of fishes, amphibians, reptiles, birds, and mammals. The study reveals that the five muscarinic receptor genes present in most tetrapods (m1-m5; except birds, which appear to lack the m1 receptor gene) derive from two ancestral genes. These two ancestral genes expanded during the first and second tetraploidizations that occurred early in vertebrate evolution, one gene producing m1/m3/m5 and the other giving rise to m2/m4. The study also shows that there was an additional expansion of the two subfamilies of muscarinic receptor genes in teleosts, possibly as a consequence of the third tetraploidization event in the teleost ancestor. As a consequence, teleost fishes contain 8 to 10 muscarinic receptor genes (all 10 genes have been retained in zebrafish), opening excellent opportunities for studies on the evolutionary origin of new receptor functions. The m2/m4 subfamily shows better conservation than the m1/m3/m5, with m1 showing the highest variation. In addition, the study shows a previously unnoticed intron gain in the coding sequence of several muscarinic receptor genes in teleost fishes.

Overall, the study provides new and relevant data on the evolution of the highly important family of cholinergic muscarinic receptors, and relates the expansion of these receptors to the different tetraploidization events that occurred during vertebrate evolution. The new results also open new venues for studies on receptor neo- or sub-functionalization.

I only have a few comments:

1. From the Introduction, it seems that different receptors overlap in some brain areas, such as the striatum and the hippocampus. In these cases, perhaps it would be more useful to give information (when available) on:

- cell types of an area where the different receptors are found (for example, projection neurons versus interneurons, etc.)

2. In this phylogenetic analysis of the muscarinic receptor genes, it would be relevant to mention available data (when possible) on the their expression and/or function in different species, and discuss how this relates to the tetraploidization events and/or the receptor gene duplication or triplication.

3. The authors compare an impressively high spectrum of species, including invertebrate chordates and different vertebrates. I congratulate them for the high number of species selected. However, they did not include jawless fishes (for example, lampreys, for which ensemble data are available). It appears that the two tetraploidization events that occurred early in vertebrate evolution happened just before or just after the lamprey-gnatostome split (Mol. Biol. Evol. 19(9):1440-1450. 2002). Therefore, including lampreys in the analysis would provide better resolution on when exactly the different duplications occurred (in the first or in the second tetraploidization), an aspect that the authors were unable to discern.

## Author Response

Changes on the authors' initiative since last submission:

- Line 23: removed “muscarinic acetylcholine receptor”

- “Teleost fishes” has been changed to “teleosts” throughout the manuscript

- Line 148: added: “However these adjustments were kept to a minimum.”

- Line 428: added Nuckels et al (2011) reference and updated sentence accordingly

- Added RRIDs to methods section

- In synteny and tree figures (Figure 2, 2-1, 2-2, 3, 3-1, 3-2), duplication scheme figure (Figure 5) and tables (Table 2-1, 3-1), genes lacking names or genes that were wrongly named have been assigned names according to “ZFIN Zebrafish Nomenclature Conventions”, for easier identification of the genes and to create analogy to another manuscript that we are preparing on the nicotinic acetylcholine receptors.

- Removed list of abbreviations

- Upon request from the eNeuro Central Office, the following Extended Data files have been renamed: Figure 1-4 (former Table 1-1), Figure 2-3 (former Table 2-1), Figure 3-3 (former Table 3-1) and Figure 4-5 (former Table 4-1)

Response to the general requirement for information on the sex of the species studied: Our study is based on data from genome databases where both sexes are usually represented. For some species, sex determination is non-chromosomal why the sex does not matter for the genome assembly.
