# Supplementary figures and images for: Evolution of the Muscarinic Acetylcholine Receptors in Vertebrates
Source: eNeuro. 2018 Nov 8;5(5):ENEURO.0340-18.2018. doi: 10.1523/ENEURO.0340-18.2018 (PMC6298421; doi:10.1523/ENEURO.0340-18.2018)

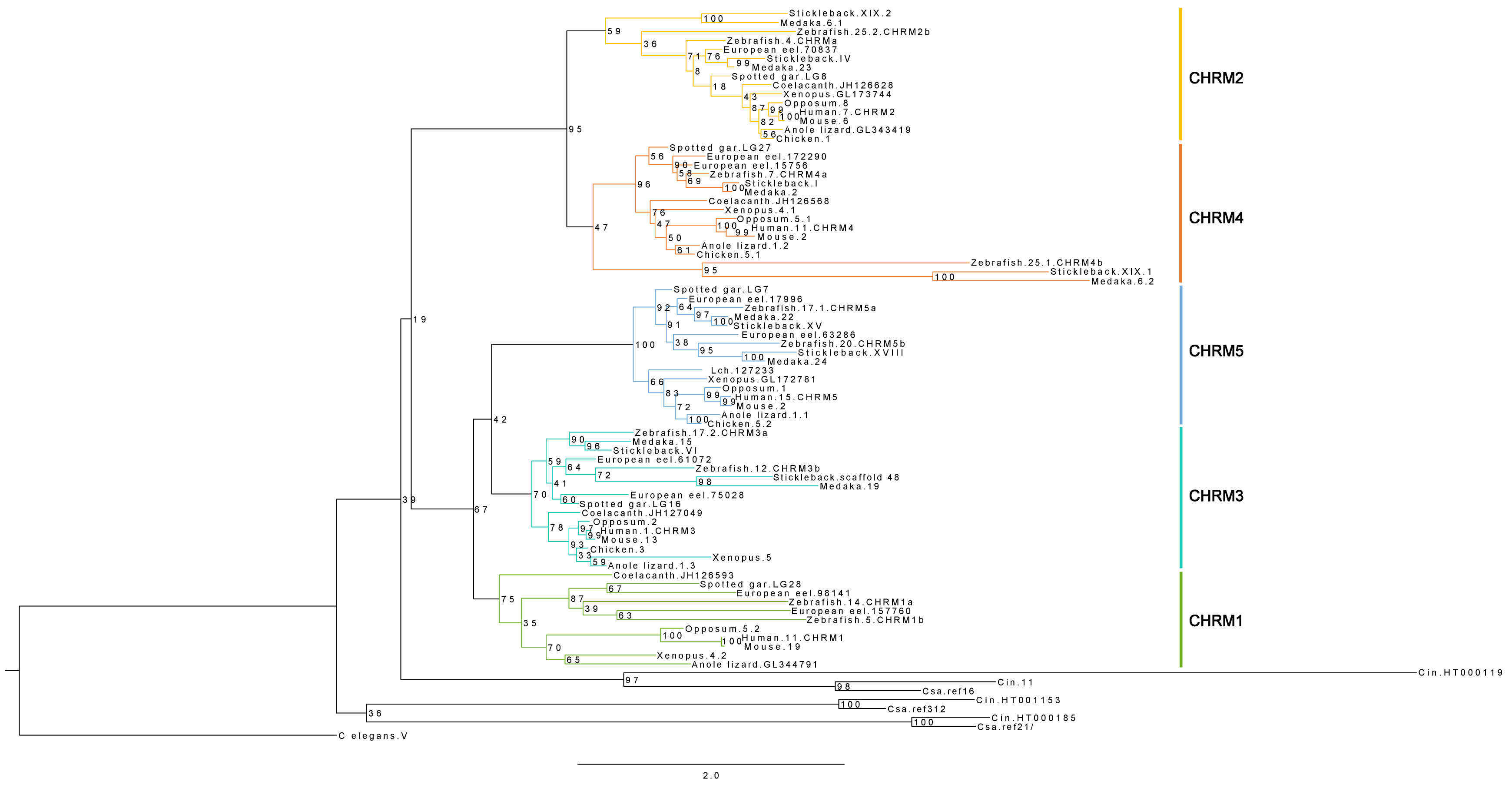

Supplement: Figure 1-3 — PhyML tree of the mAChR genes (CHRM1–CHRM5), rooted with C. elegans. The tree topology is supported by a nonparametric bootstrap analysis with 100 replicates. This tree is based on the complete multiple sequence alignment (i.e. the IL3 region is also included). In the sequence names, the species is followed by the chromosome or genomic scaffold at which the gene is located (numbers or roman numerals). If several genes are located on the same chromosome or genomic scaffold, their order is indicated by an additional number. Cin, Ciona intestinalis; Csa, Ciona savigny. Download Fig. 1-3, TIF file. [file sup_enu-eN-NWR-0340-18-s03.tif]

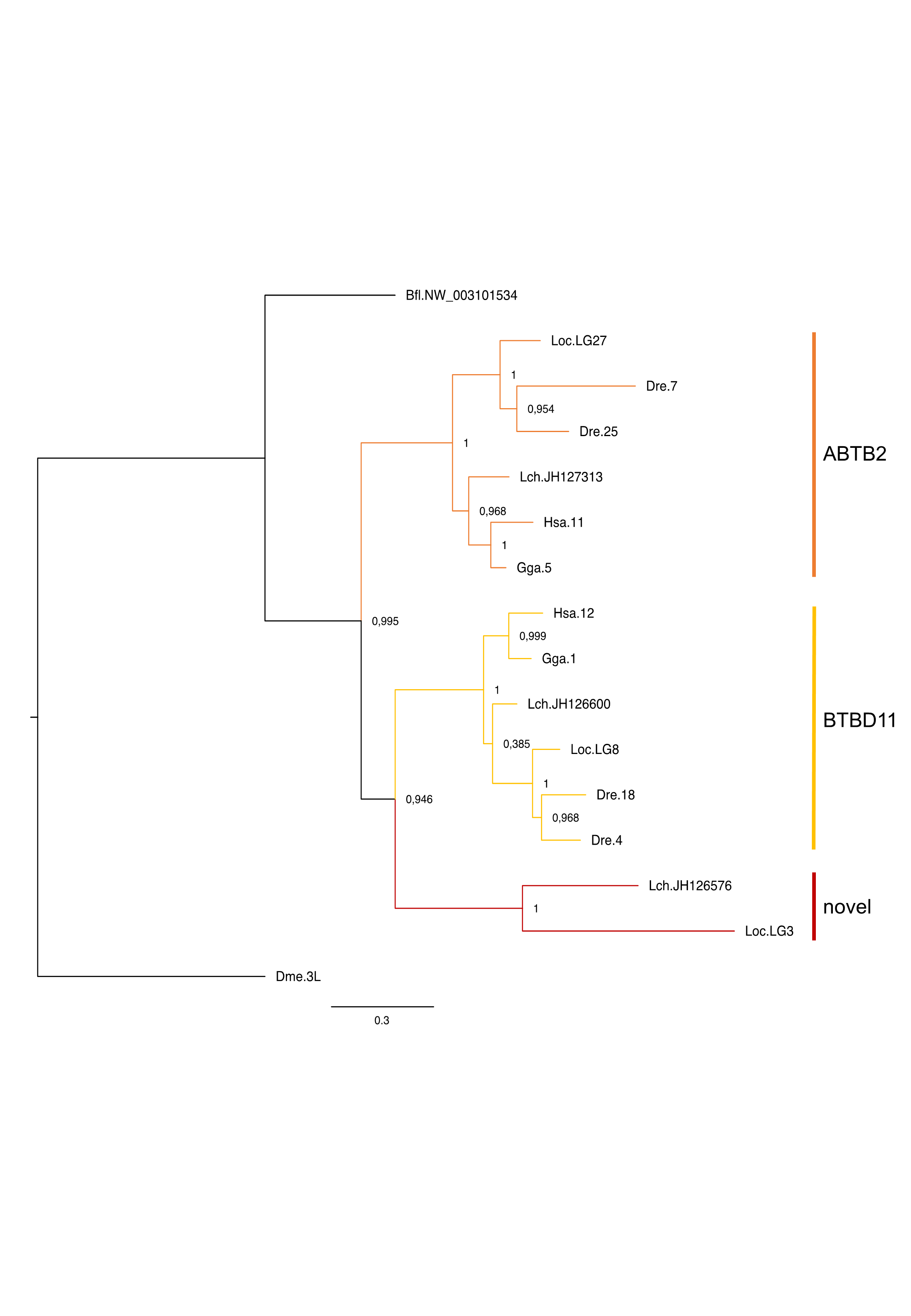

Supplement: Figure 2-1 — Phylogenetic aLRT SH-like tree of the neighboring gene families included in analysis of the CHRM2 and CHRM4 genomic regions. There is one neighboring gene family per page in alphabetical order. Orthologs are color coded according to paralogon member. Gray color means that those orthologs are not included in paralogon analysis due to exclusion based on results from the phylogenetic analysis, or that there is no human, chicken, or spotted gar sequence present. Assigned sequence names and sequence information details are provided in Fig. 2-3. Download Fig. 2-1, TIF file. [file sup_enu-eN-NWR-0340-18-s05.tif]

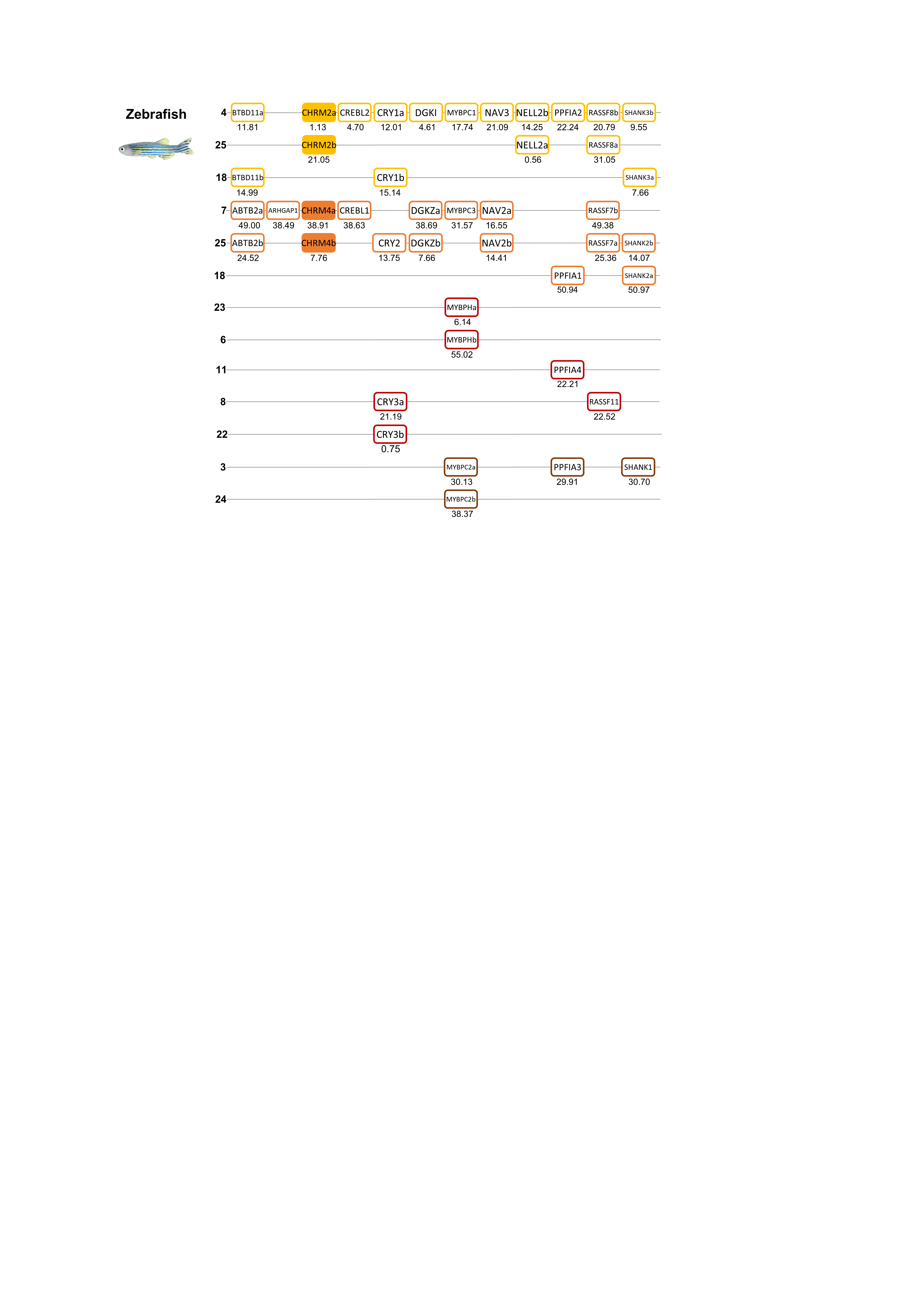

Supplement: Figure 2-2 — Analysis of chromosomal positions of the CHRM2 and CHRM4 genes and their neighboring gene families included in the paralogon analysis in Figure 2 in the zebrafish. Each paralogon member is presented in a separate color. The zebrafish illustration is reused with permission from Daniel Ocampo Daza (source: www.egosumdaniel.se). Download Fig. 2-2, TIF file. [file sup_enu-eN-NWR-0340-18-s06.tif]

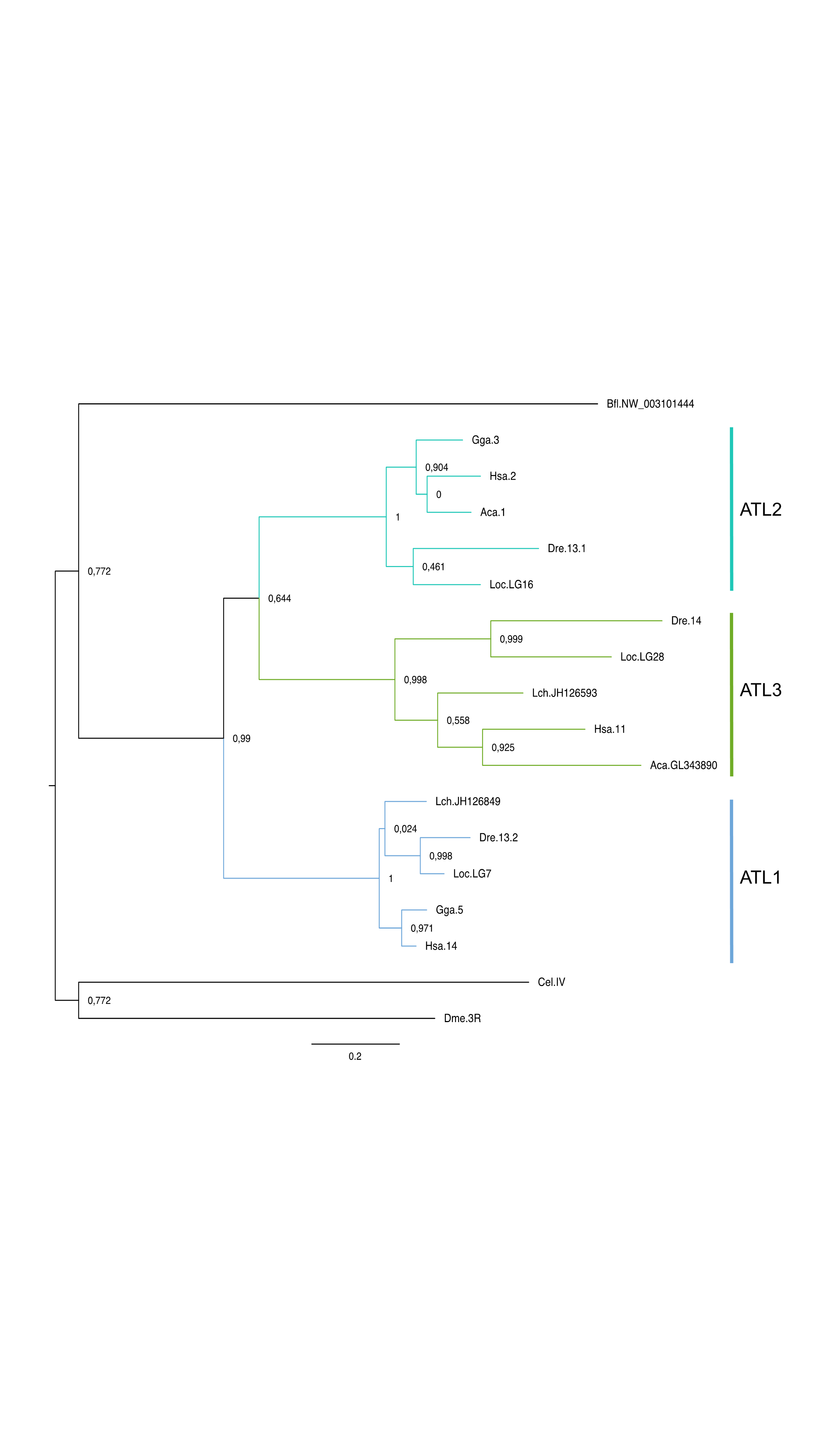

Supplement: Figure 3-1 — aLRT SH-like tree of the neighboring gene families included in analysis of the CHRM1, CHRM3, and CHRM5 genomic regions. There is one neighboring gene family per page in alphabetical order. Orthologs are color coded according to paralogon member. Gray color means that those orthologs are not included in paralogon analysis due to exclusion based on results from the phylogenetic analysis, or that there is no human, chicken, or spotted gar sequence present. Two trees are included for the following gene families: LTBP, PROX, and SPTB, where the first tree includes all sequences and the second one contains all sequences except the Loc.LG28, due to its shortness in length. There are two trees included also for the PLD gene family, the first tree is based on complete alignment, and the second tree is based on an alignment where the first part of alignment is excluded due to a very low degree of sequence conservation and highly variable sequence lengths. Assigned sequence names and sequence information details are provided in Fig. 3-3. Download Fig. 3-1, TIF file. [file sup_enu-eN-NWR-0340-18-s08.tif]

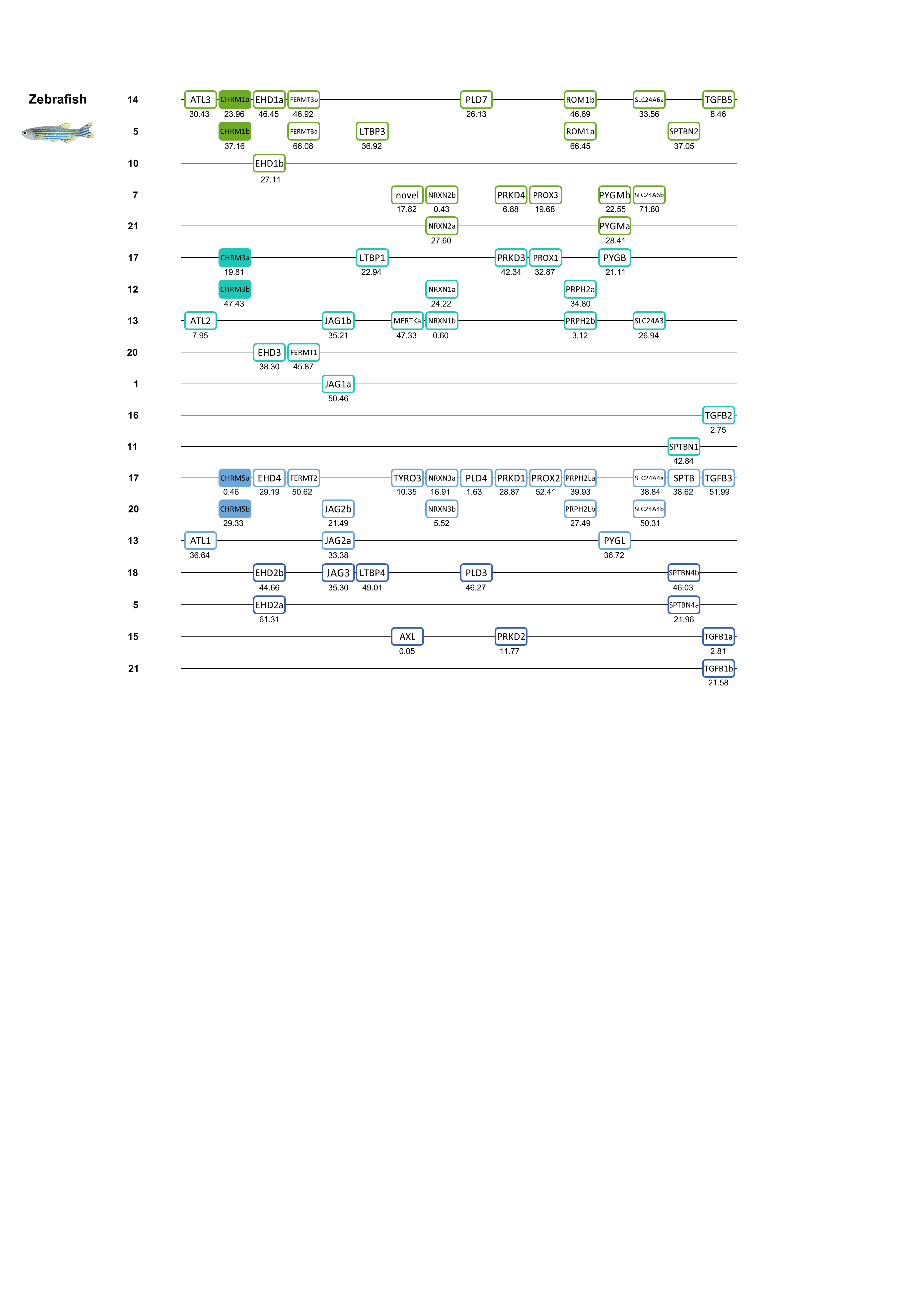

Supplement: Figure 3-2 — Analysis of chromosomal positions of the CHRM1, CHRM3, and CHRM5 genes and their neighboring gene families included in the paralogon analysis in Figure 3 in the zebrafish. Each paralogon member is presented in a separate color. The zebrafish illustration is reused with permission from Daniel Ocampo Daza (source: www.egosumdaniel.se). Download Fig. 3-2, TIF file. [file sup_enu-eN-NWR-0340-18-s09.tif]

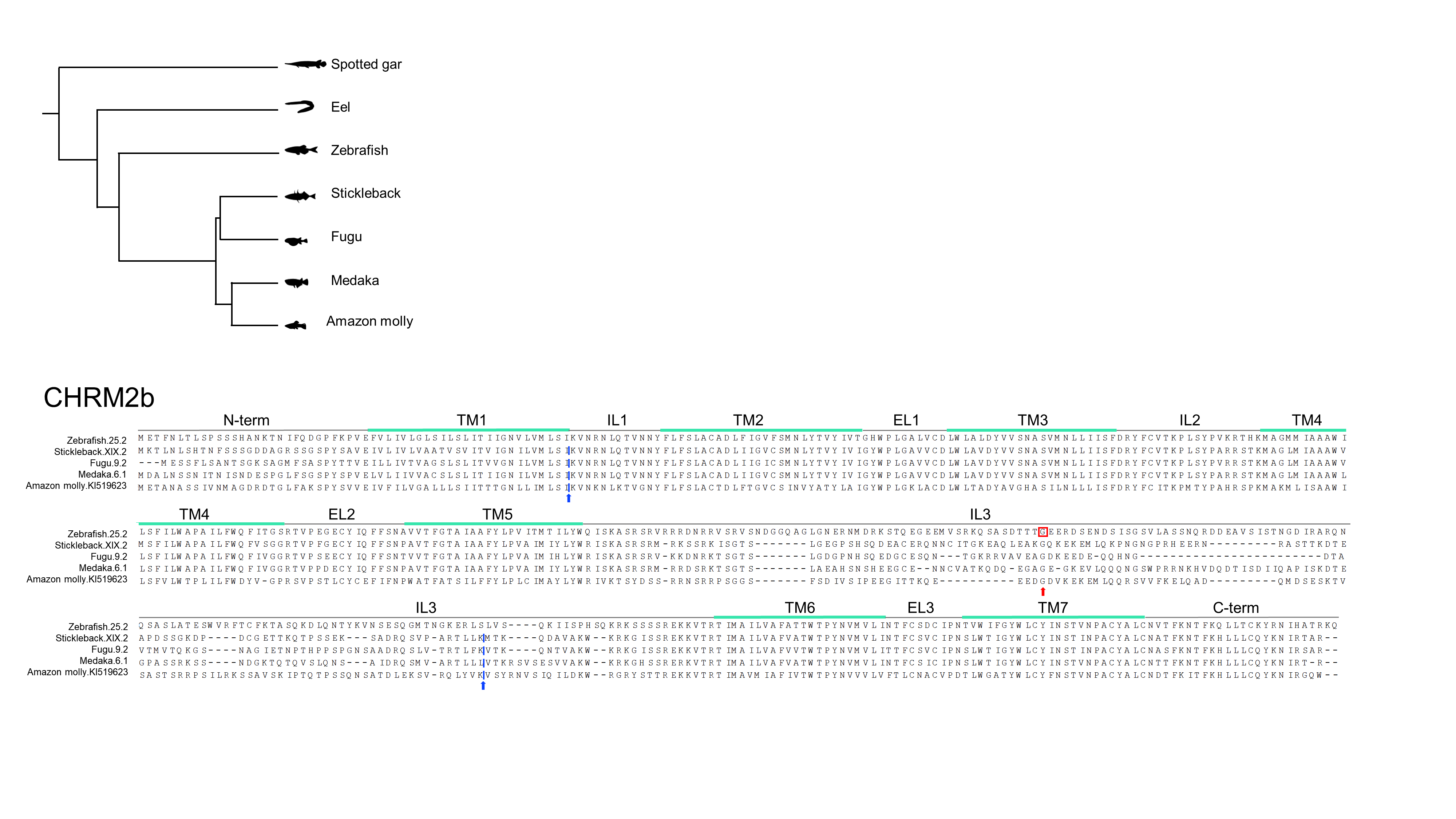

Supplement: Figure 4-1 — Jalview sequence alignment of the teleost species sequences included in the analysis of teleost-specific intron gains in the CHRM2b, CHRM3b, CHRM4a, and CHRM4b genes. The top line displays predicted protein domains. Arrow indicates intron gain. Square indicates phase 1 or phase 2 splicing, and line indicates phase 0 splice. Introns with certain positions are marked in red or blue. Intron locations with uncertain positions, due to sequence quality or species inconsistencies, are marked in gray. The spotted gar and stickleback illustrations are reused with permission from Milton Tan (https://creativecommons.org/licenses/by-nc-sa/3.0/). Download Fig. 4-1, TIF file. [file sup_enu-eN-NWR-0340-18-s11.tif]

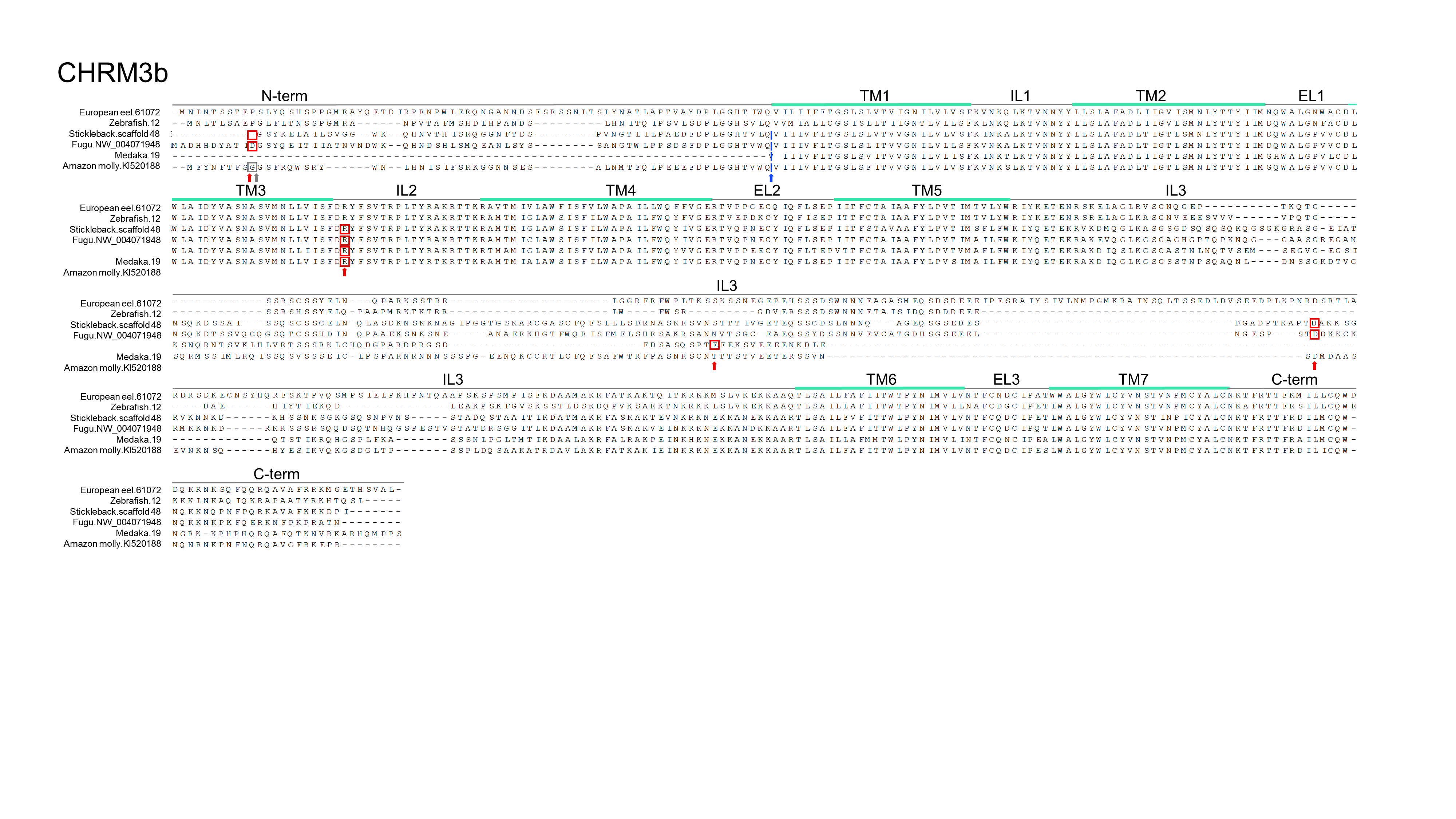

Supplement: Figure 4-2 — Supplementary Figure 4-2. Download Fig. 4-2, TIF file. [file sup_enu-eN-NWR-0340-18-s12.tif]

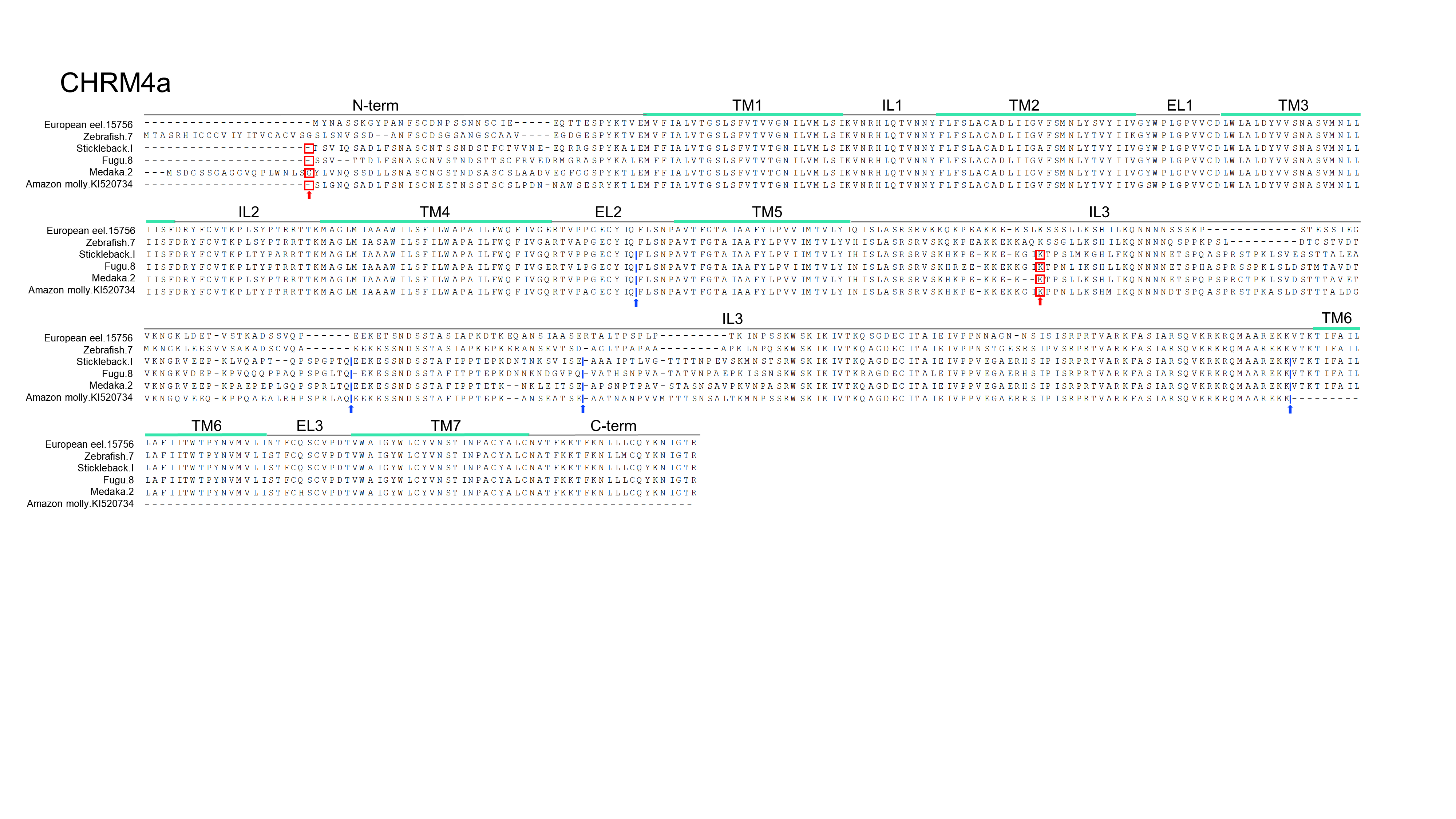

Supplement: Figure 4-3 — Supplementary Figure 4-3. Download Fig. 4-3, TIF file. [file sup_enu-eN-NWR-0340-18-s13.tif]

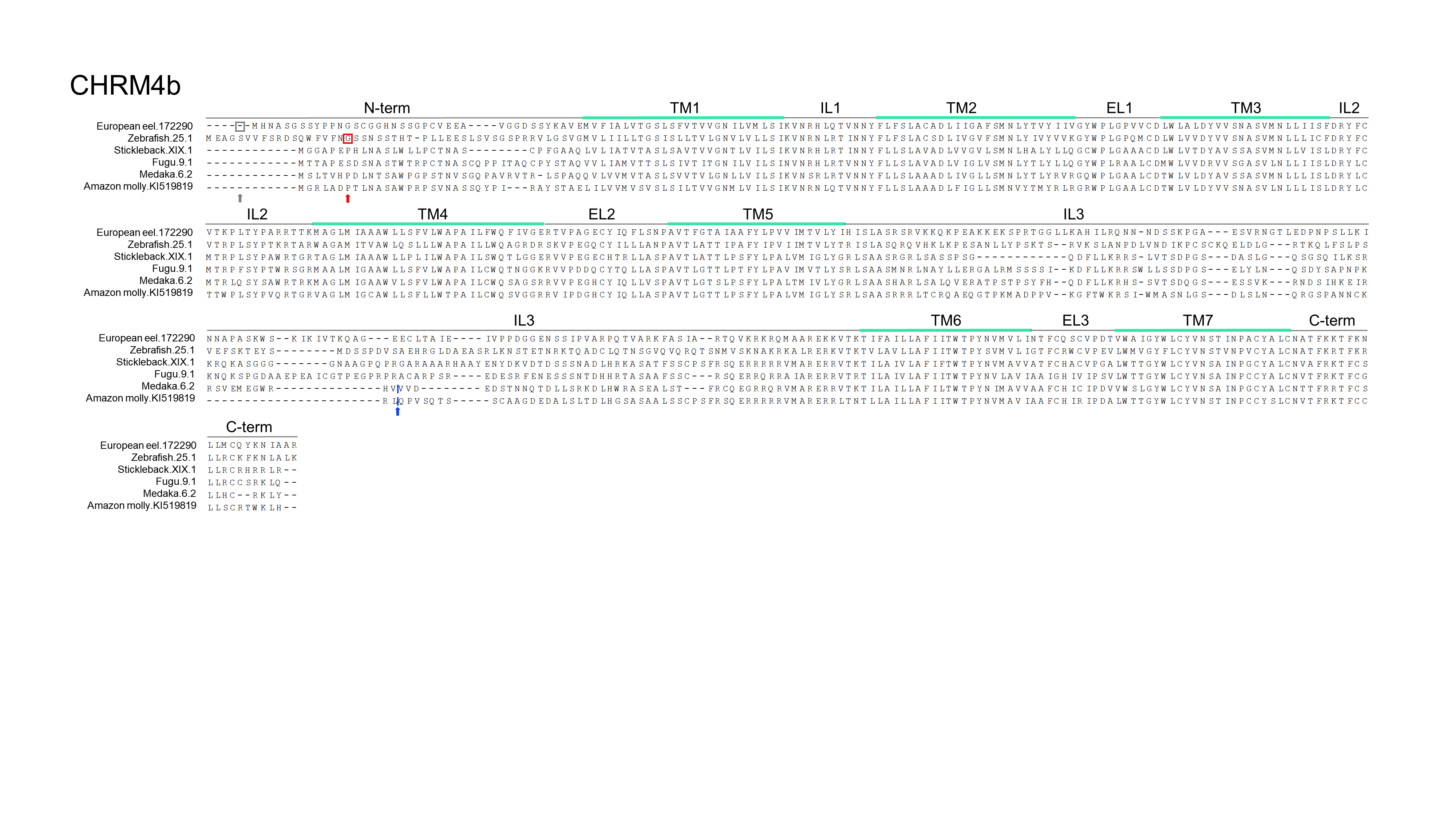

Supplement: Figure 4-4 — Supplementary Figure 4-4. Download Fig. 4-4, TIF file. [file sup_enu-eN-NWR-0340-18-s14.tif]
