# Supplementary material for: Evolution of the Muscarinic Acetylcholine Receptors in Vertebrates
Source: eNeuro. 2018 Nov 8;5(5):ENEURO.0340-18.2018. doi: 10.1523/ENEURO.0340-18.2018 (PMC6298421; doi:10.1523/ENEURO.0340-18.2018)
Supplement: Figure 1-4 — Information about the mAChR amino acid sequences retrieved and included in the analysis. First, the genome assembly versions used are stated, followed by information about the mAChR gene sequences included in the analysis in the following order: species, HGNC/ZFIN/Flybase symbol name, chromosome or genomic scaffold position, orientation, Ensembl ID or NCBI accession number, assigned sequence name in alignment, assigned sequence name in PhyML tree, and additional comments regarding sequence update date on NCBI or whether there have been manual edits of the original Ensembl or NCBI sequence. The order of sequences is organized according to orthology. Download Fig. 1-4, DOCX file. [file sup_enu-eN-NWR-0340-18-s04.docx]

| **Ens87** | | **Common name** | **Scientific name** | **Abbrevation** | **Assembly Ensembl** | **Assembly NCBI** |  |  |  |
| --- | --- | --- | --- | --- | --- | --- | --- | --- | --- |
|  | | Anole lizard | *Anolis carolinensis* | Aca | AnoCar2.0 | AnoCar2.0 |  |  |  |
|  | | Amphioxus | *Branchiostoma floridae* | Bfl |  | GCA_000003815.1 Version 2 |  |  |  |
|  | | *Ciona intestinalis* | *Ciona intestinalis* | Cin | KH |  |  |  |  |
|  | | *Ciona savignyi* | *Ciona savignyi* | Csa | CSAV 2.0 |  |  |  |  |
|  | | *Caenorhabditis elegans* | *Caenorhabditis elegans* | Cel | WBcel235 |  |  |  |  |
|  | | Chicken | *Gallus gallus* | Gga | Gallus_gallus-5.0 |  |  |  |  |
|  | | Coelacanth | *Latimeria chalumnae* | Lch | LatCha1 | LatCha1 |  |  |  |
|  | | European Eel | *Anguilla Anguilla* | Aan |  | Anguilla_anguilla_v1_09_nov_10 |  |  |  |
|  | | Frog | *Xenopus tropicalis* | Xtr | JGI 4.2 | Xenopus_tropicalis_v9.1 |  |  |  |
|  | | Human | *Homo sapiens* | Hsa | GRCh38.p7 |  |  |  |  |
|  | | Medaka | *Oryzias latipes* | Ola | HdrR | ASM223467v1 |  |  |  |
|  | | Mouse | *Mus musculus* | Mmu | GRCm38.p4 |  |  |  |  |
|  | | Opposum | *Monodelphis domestica* | Mdo | monDom5 |  |  |  |  |
|  | | Spotted gar | *Lepisosteus oculatus* | Loc | LepOcu1 | LepOcu1 |  |  |  |
|  | | Stickleback | *Gasterosteus oculeatus* | Gac | BROAD S1 |  |  |  |  |
|  | | Zebrafish | *Danio rerio* | Dre | GRCz10 | GRCz11 |  |  |  |
| **Species** | | **HGNC/ZFIN/Flybase symbol** | **Chromosome/ genomic scaffold** | **Orientation** | **Ensembl Gene ID/Other accession nr** | **Ensembl Transcript ID** | **Assigned sequence name Jalview alignment** | **Assigned sequence name PhyML tree** | **Comme-nts** |
| Human | | **CHRM1** | 11: 62.91m | rev | ENSG00000168539 | ENST00000306960.3 | Hsa.11.CHRM1 | Human.11.CHRM1 |  |
| Mouse | |  | 19: 8.66m | fwd | ENSMUSG00000032773 | ENSMUSG00000032773 | Mmu.19 | Mouse.19 |  |
| Opossum | |  | 5: 300.81m | rev | ENSMODG00000008164 | ENSMODT00000010339.2 | Mdo.5.2 | Opposum.5.2 |  |
| Anole lizard | |  | GL344791.1: 0.15m | rev | ENSACAG00000010234 | ENSACAT00000010234.2 | Aca.344791 | Anole lizard.GL344791 | *manually edited* |
| Frog | |  | 4: 36.05m | fwd | XP_004913718.1 |  | Xtr.4.2 | Xenopus.4.2 |  |
| Coelacanth | |  | JH126593.1: 0.46m | fwd | ENSLACG00000009972 | ENSLACT00000011420.1 | Lch.126593 | Coelacanth.JH126593 | *manually edited* |
| Spotted gar | |  | LG28: 3.48m | rev | ENSLOCG00000017613 | ENSLOCT00000021755.1 | Loc.LG28 | Spotted gar.LG28 |  |
| Zebrafish | | CHRM1a | 14: 23.96m | fwd | ENSDARG00000037292 | ENSDART00000173227.1 | Dre.14 | Zebrafish.14.CHRM1a |  |
|  | | CHRM1b | 5: 37.16m | rev | ENSDARG00000009121 | ENSDART00000020646.7 | Dre.5 | Zebrafish.5.CHRM1b |  |
| European eel | |  | contig_98141 |  | AZBK01767327.1 |  | Aan.98141 | European eel.98141 | may-14, *manually edited* |
|  | |  | contig_157760 |  | AZBK01707708.1 |  | Aan.157760 | European eel.157760 | may-14, *manually edited* |
| Human | | **CHRM2** | 7: 136.87m | fwd | ENSG00000181072 | ENST00000445907.6 | Hsa.7.CHRM2 | Human.7.CHRM2 |  |
| Mouse | |  | 6: 36.39m | fwd | ENSMUSG00000045613 | ENSMUST00000172278.7 | Mmu.6 | Mouse.6 |  |
| Opossum | |  | 8: 198.46m | fwd | ENSMODG00000024440 | ENSMODT00000035723.1 | Mdo.8 | Opposum.8 |  |
| Chicken | |  | 1: 57.86m | rev | ENSGALG00000012894 | ENSGALT00000021029.3 | Gga.1 | Chicken.1 |  |
| Anole lizard | |  | GL343419.1: 0.35m | rev | ENSACAG00000012100 | ENSACAT00000012077.1 | Aca.343419 | Anole lizard.GL343419 |  |
| Frog | |  | GL173744.1: 0.06m | rev | ENSXETG00000020175 | ENSXETT00000043526.3 | Xtr.173744 | Xenopus.GL173744 |  |
| Coelacanth | |  | JH126628.1: 1.45m | rev | ENSLACG00000016094 | ENSLACT00000018403.1 | Lch.126628 | Coelacanth.JH126628 |  |
| Spotted gar | |  | LG8: 38.28m | fwd | ENSLOCG00000017918 | ENSLOCT00000022060.1 | Loc.LG8 | Spotted gar.LG8 |  |
| Zebrafish | | CHRM2b | 25: 21.05m | fwd | XP_021326218.1 |  | Dre.25.2 | Zebrafish.25.2.CHRM2b |  |
|  | | CHRM2a | 4: 1.13m | fwd | ENSDARG00000098612 | ENSDART00000167233.1 | Dre.4 | Zebrafish.4.CHRM2a |  |
| Medaka | |  | 6: 21.31m | fwd | XP_004069752.1 |  | Ola.6.1 | Medaka.6.1 | feb-18 |
|  | |  | 23: 18.63m | fwd | XP_020569752.1 |  | Ola.23 | Medaka.23 | feb-18 |
| Stickleback | |  | groupXIX: 15.19m | rev | ENSGACG00000011914 | ENSGACT00000015788.1 | Gac.XIX.2 | Stickleback.XIX.2 | *manually edited* |
|  | |  | groupIV: 31.17m | rev | ENSGACG00000019948 | ENSGACT00000026406.1 | Gac.IV | Stickleback.IV | *manually edited* |
| European eel | |  | contig_70837 |  | AZBK01794631 |  | Aan.70837 | European eel.70837 | may-14 |
| Human | | **CHRM3** | 1: 239.39m | fwd | ENSG00000133019 | ENST00000255380.8 | Hsa.1.CHRM3 | Human.1.CHRM3 |  |
| Mouse | |  | 13: 9.88m | rev | ENSMUSG00000046159 | ENSMUST00000187510.6 | Mmu.13 | Mouse.13 |  |
| Opossum | |  | 2: 148.69m | rev | ENSMODG00000024652 | ENSMODT00000036568.1 | Mdo.2 | Opposum.2 |  |
| Chicken | |  | 3: 36.53m | rev | ENSGALG00000010778 | ENSGALT00000017530.4 | Gga.3 | Chicken.3 | *manually edited* |
| Anole lizard | |  | 1: 217.19m | rev | ENSACAG00000003639 | ENSACAT00000003615.2 | Aca.1.3 | Anole lizard.1.3 | *manually edited* |
| Frog | |  | 5: 59.93m | fwd | XP_002935876.1 |  | Xtr.5 | Xenopus.5 | sep-16 |
| Coelacanth | |  | JH127049.1: 0.03m | fwd | ENSLACG00000022555 | ENSLACT00000025889.1 | Lch.127049 | Coelacanth.JH127049 | *manually edited* |
| Spotted gar | |  | LG16: 15.78m | rev | ENSLOCG00000017368 | ENSLOCT00000021510.1 | Loc.LG16 | Spotted gar.LG16 |  |
| Zebrafish | | CHRM3a | 17: 19.81m | rev | ENSDARG00000071091 | ENSDART00000154251.1 | Dre.17.2 | Zebrafish.17.2.CHRM3a |  |
|  | | CHRM3b | 12: 47.43m | fwd | ENSDARG00000071298 | ENSDART00000105331.4 | Dre.12 | Zebrafish.12.CHRM3b |  |
| Medaka |  | | 15: 18.08m | fwd | XP_011482665.1 |  | Ola.15 | Medaka.15 | feb-18, *manually edited* |
|  | |  | 19: 17.01m | rev | XP_023805573.1 |  | Ola.19 | Medaka.19 | feb-18, *manually edited* |
| Stickleback | |  | groupVI: 12.30m | rev | ENSGACG00000010124 | ENSGACT00000013390.1 | Gac.VI | Stickleback.VI | *manually edited* |
|  | |  | scaffold_48: 1.06m | rev | ENSGACG00000015318 | ENSGACT00000020240.1 | Gac.sc48 | Stickleback.scaffold 48 | *manually edited* |
| European eel | |  | contig_61072 |  | AZBK01804396.1 |  | Aan.61072 | European eel.61072 | may-14, *manually edited* |
|  | |  | contig_75028 |  | AZBK01790440.1 |  | Aan.75028 | European eel.75028 | May-14, *manually edited* |
| Human | | **CHRM4** | 11: 46.39m | rev | ENSG00000180720 | ENST00000433765.3 | Hsa.11.CHRM4 | Human.11.CHRM4 |  |
| Mouse | |  | 2: 91.93m | fwd | ENSMUSG00000040495 | ENSMUST00000045537.3 | Mmu.2.1 | Mouse.2 |  |
| Opossum | |  | 5: 283.47m | rev | XP_007497444.1 |  | Mdo.5.1 | Opposum.5.1 |  |
| Chicken | |  | 5: 23.65m | fwd | ENSGALG00000008365 | ENSGALT00000013620.4 | Gga.5.1 | Chicken.5.1 | *manually edited* |
| Anole lizard | |  | 1: 42.63m | rev | ENSACAG00000004120 | ENSACAT00000004095.3 | Aca.1.2 | Anole lizard.1.2 | *manually edited* |
| Frog | |  | 4: 5.22m | fwd | NP_001106514.1 |  | Xtr.4.1 | Xenopus.4.1 | okt-16 |
| Coelacanth | |  | JH126568.1: 4.19m | rev | ENSLACG00000018713 | ENSLACT00000021435.1 | Lch.126568 | Coelacanth.JH126568 | *manually edited* |
| Spotted gar | |  | LG27: 11.86m | fwd | ENSLOCG00000005536 | ENSLOCT00000006691.1 | Loc.LG27 | Spotted gar.LG27 |  |
| Zebrafish | | CHRM4a | 7: 38.91m | rev | ENSDARG00000069254 | ENSDART00000100639.4 | Dre.7 | Zebrafish.7.CHRM4a | *manually edited* |
|  | | CHRM4b | 25: 7.76m | rev | ENSDARG00000017722 | ENSDART00000157276.1 | Dre.25.1 | Zebrafish.25.1.CHRM4b |  |
| Medaka | |  | 2: 1.07m | rev | XP_023817375.1 |  | Ola.2 | Medaka.2 | feb-18 |
|  | |  | 6: 26.80m | fwd | XP_011474704.2 |  | Ola.6.2 | Medaka.6.2 | feb-18 |
| Stickleback | |  | groupI: 27.13m | rev | ENSGACG00000015349 | ENSGACT00000020282.1 | Gac.I | Stickleback.I | *manually edited* |
|  | |  | groupXIX: 8.95m | fwd | ENSGACG00000007854 | ENSGACT00000010430.1 | Gac.XIX.1 | Stickleback.XIX.1 | *manually edited* |
| European eel | |  | contig_172290 |  | AZBK01693178.1 |  | Aan.172290 | European eel.172290 | may-14, *manually edited* |
|  | |  | contig_15756 |  | AZBK01849712.1 |  | Aan.15756 | European eel.15756 | may-14, *manually edited* |
| Human | | **CHRM5** | 15: 33.97m | fwd | ENSG00000184984 | ENST00000383263.6 | Hsa.15.CHRM5 | Human.15.CHRM5 |  |
| Mouse | |  | 2: 112.48m | rev | ENSMUSG00000074939 | ENSMUST00000099589.3 | Mmu.2.2 | Mouse.2 |  |
| Opossum | |  | 1: 187.21m | rev | ENSMODG00000024252 | ENSMODT00000035304.1 | Mdo.1 | Opposum.1 |  |
| Chicken | |  | 5: 30.08m | rev | ENSGALG00000009640 | ENSGALT00000015701.3 | Gga.5.2 | Chicken.5.2 |  |
| Anole lizard | |  | 1: 33.66m | fwd | ENSACAG00000015381 | ENSACAT00000015410.2 | Aca.1.1 | Anole lizard.1.1 |  |
| Frog | |  | GL172781.1: 0.28m | fwd | ENSXETG00000017868 | ENSXETT00000054915.3 | Xtr.172781 | Xenopus.GL172781 |  |
| Coelacanth | |  | JH127233.1: 0.39m | rev | ENSLACG00000009104 | ENSLACT00000010416.1 | Lch.127233 | Coelacanth.JH127233 |  |
| Spotted gar | |  | LG7: 15.11m | rev | ENSLOCG00000017709 | ENSLOCT00000021851.1 | Loc.LG7 | Spotted gar.LG7 |  |
| Zebrafish | | CHRM5a | 17: 0.46m | rev | ENSDARG00000099928 | ENSDART00000168718.1 | Dre.17.1 | Zebrafish.17.1.CHRM5a |  |
|  | | CHRM5b | 20: 29.33m | rev | ENSDARG00000069598 | ENSDART00000101418.4 | Dre.20 | Zebrafish.20.CHRM5b | *manually edited* |
| Medaka | |  | 22: 14.14m | rev | XP_020569528.1 |  | Ola.22 | Medaka.22 | feb-18 |
|  | |  | 24: 20.00m | rev | XP_023808668.1 |  | Ola.24 | Medaka.24 | feb-18 |
| Stickleback | |  | groupXV: 2.35m | rev | ENSGACG00000006035 | ENSGACT00000008002.1 | Gac.XV | Stickleback.XV |  |
|  | |  | groupXVIII: 6.86m | rev | ENSGACG00000007923 | ENSGACT00000010528.1 | Gac.XVIII | Stickleback.XVIII | *manually edited* |
| European eel | |  | contig_17996 |  | AZBK01847472.1 |  | Aan.17996 | European eel.17996 | may-14, *manually edited* |
|  | |  | contig_63286 |  | AZBK01802182.1 |  | Aan.63286 | European eel.63286 | may-14, *manually edited* |
| Ciona intestinalis | |  | 11: 4.28m | rev | ENSCING00000019975 | ENSCINT00000030829.1 | Cin.11 | Cin.11 |  |
|  | |  | Scaffold HT000119.1: 0.34m | rev | ENSCING00000003264 | ENSCINT00000006703.3 | Cin.HT19 | Cin.HT000119 |  |
|  | |  | Scaffold HT000185.1: 0.02m | rev | ENSCING00000008896 | ENSCINT00000018103.3 | Cin.HT85 | Cin.HT000185 |  |
|  | |  | Scaffold HT001153.1: 0.02m | fwd | ENSCING00000007685 | ENSCINT00000015775.3 | Cin.HT53 | Cin.HT001153 |  |
| Ciona savigny | |  | reftig_16: 3.64m | rev | ENSCSAVG00000011335 | ENSCSAVT00000019512.1 | Csa.ref16 | Csa.ref16 |  |
|  | |  | reftig_21: 0.04m | rev | ENSCSAVG00000002827 | ENSCSAVT00000004815.1 | Csa.ref21 | Csa.ref21 |  |
|  | |  | reftig_312: 0.44m | rev | ENSCSAVG00000000994 | ENSCSAVT00000001744.1 | Csa.ref312 | Csa.ref312 |  |
| C. elegans | |  | V: 14.56m | rev | WBGene00001519 | Y40H4A.1b | Cel.V | C. elegans.V |  |
