# Supplementary material for: Evolution of the Muscarinic Acetylcholine Receptors in Vertebrates
Source: eNeuro. 2018 Nov 8;5(5):ENEURO.0340-18.2018. doi: 10.1523/ENEURO.0340-18.2018 (PMC6298421; doi:10.1523/ENEURO.0340-18.2018)
Supplement: Figure 2-3 — Information about the neighboring gene family sequences included in the analysis of the genomic regions surrounding the CHRM2 and CHRM4 genes. First, the genome assembly versions used are stated, followed by information about the neighbor gene families included in the analysis in the following order: species, HGNC/ZFIN/Flybase symbol name, chromosome or genomic scaffold position, Ensembl ID or NCBI accession number, assigned sequence name, and additional comments regarding the sequence update date on NCBI or whether there have been manual edits of the original Ensembl or NCBI sequence. Download Fig. 2-3, DOCX file. [file sup_enu-eN-NWR-0340-18-s07.docx]

| **Ens87** | **Common name** | **Scientific name** | **Abbrevation** | **Assembly Ensembl** | **Assembly NCBI** |  |
| --- | --- | --- | --- | --- | --- | --- |
|  | Anole lizard | *Anolis carolinensis* | Aca | AnoCar2.0 | AnoCar2.0 |  |
|  | Amphioxus | *Branchiostoma floridae* | Bfl | GCA_000003815.1 Version 2 |  |  |
|  | *Ciona intestinalis* | *Ciona intestinalis* | Cin | KH |  |  |
|  | *Ciona savignyi* | *Ciona savignyi* | Csa | CSAV 2.0 |  |  |
|  | *Caenorhabditis elegans* | *Caenorhabditis elegans* | Cel | WBcel235 |  |  |
|  | Chicken | *Gallus gallus* | Gga | Gallus_gallus-5.0 |  |  |
|  | Chinese softshell turtle | *Pelodiscus sinensis* | Psi | PelSin_1.0 |  |  |
|  | Coelacanth | *Latimeria chalumnae* | Lch | LatCha1 | LatCha1 |  |
|  | Fruitfly | *Drosophila melanogaster* | Dme | BDGP6 |  |  |
|  | Human | *Homo sapiens* | Hsa | GRCh38.p7 |  |  |
|  | Medaka | *Oryzias latipes* | Ola | HdrR |  |  |
|  | Purple sea urchin | *Strongylocentrotus purpuratus* | Spu | Spur_4.2 |  |  |
|  | Spotted gar | *Lepisosteus oculatus* | Loc | LepOcu1 | LepOcu1 |  |
|  | Zebrafish | *Danio rerio* | Dre | GRCz10 | GRCz11 |  |
|  |  |  |  |  |  |  |
| **Species** | **HGNC,ZFIN or FlyBase name** | **Chromosome/scaffold locations** | **Ensembl ID or NCBI accession number** | **Transcript ID** | **Assigned sequence name** | **Comments/Annotation notes** |
| Human | ABTB2 | 11: 34.15m | ENSG00000166016 | ENST00000435224.2 | Hsa.11 |  |
|  | BTBD11 | 12: 107.32m | ENSG00000151136 | ENST00000280758.9 | Hsa.12 |  |
| Chicken |  | 5: 18.54m | XP_423445.3 |  | Gga.5 |  |
|  |  | 1: 53.59m | XP_015143383.1 |  | Gga.1 |  |
| Coelacanth | | JH127313.1: 161.11k | ENSLACG00000005390 | ENSLACT00000006125.2 | Lch.JH127313 |  |
|  |  | JH126576.1: 456.45k | ENSLACG00000009938 | ENSLACT00000011383.2 | Lch.JH126576 | assigned family member name in tree: novel |
|  |  | JH126600.1: 662.20k | ENSLACG00000012005 | ENSLACT00000013736.1 | Lch.JH126600 | manually edited |
|  |  | JH126600.1: 459.41k | ENSLACG00000009924 | ENSLACT00000011366.1 |  | manually edited |
| Spotted gar | | LG3: 36.86m | ENSLOCG00000012541 | ENSLOCT00000015480.1 | Loc.LG3 | assigned family member name in synteny figure and tree: novel |
|  |  | LG8: 4.10m | ENSLOCG00000015275 | ENSLOCT00000018831.1 | Loc.LG8 |  |
|  |  | LG27: 4.83m | ENSLOCG00000001988 | ENSLOCT00000002321.1 | Loc.LG27 |  |
| Zebrafish | abtb2a | 7: 49.00m | ENSDARG00000059751 | ENSDART00000083389.6 | Dre.7 |  |
|  | abtb2b | 25: 24.52m | ENSDARG00000062000 | ENSDART00000089113.5 | Dre.25 |  |
|  | btbd11a | 4: 11.81m | ENSDARG00000063255 | ENSDART00000092250.6 | Dre.4 |  |
|  | btbd11b | 18: 14.99m | ENSDARG00000063040 | ENSDART00000172273.1 | Dre.18 |  |
| Amphioxus |  | NW_003101534.1:5.73m | XP_002608561.1 |  | Bfl.NW_003101534 |  |
| Fruitfly | CG43980 | 3L: 21.64m | FBgn0264711 | FBtr0333974 | Dme.3L |  |
|  |  |  |  |  |  |  |
| **Species** | **HGNC,ZFIN or FlyBase name** | **Chromosome/scaffold locations** | **Ensembl ID or NCBI accession number** | **Transcript ID** | **Assigned sequence name** | **Comments/Annotation notes** |
| Human | ARHGAP1 | 11: 46.68m | ENSG00000175220 | ENST00000311956.8 | Hsa.11 |  |
|  | ARHGAP8 | 22: 44.75m | ENSG00000241484 | ENST00000356099.10 | Hsa.22 |  |
| Chicken |  | 5: 23.45m | ENSGALG00000042357 | ENSGALT00000079282.1 | Gga.5 |  |
|  |  | 1: 69.90m | ENSGALG00000019261 | ENSGALT00000022983.4 | Gga.1 |  |
| Coelacanth | | JH126568.1: 4.57m | ENSLACG00000018798 | ENSLACT00000021536.1 | Lch.JH126568 | manually edited |
|  |  | NW_005819187.1:0.88m | XP_005993733.1 |  | Lch.NW_005819187 | oct-15 |
| Spotted gar | | LG27: 12.31m | ENSLOCG00000005713 | ENSLOCT00000006907.1 | Loc.LG27 |  |
|  |  | LG8: 37.24m | ENSLOCG00000016538 | ENSLOCT00000020461.1 | Loc.LG8 |  |
| Zebrafish | arhgap1 | 7: 38.49m | ENSDARG00000024324 | ENSDART00000036461.5 | Dre.7 |  |
| Cave fish |  | Scaffold KB882095.1: 168.85k | ENSAMXG00000008329 | ENSAMXT00000008559.1 | Ame.KB882095 |  |
|  |  | Scaffold KB882086.1: 3.08m | ENSAMXG00000021366 | ENSAMXT00000021997.1 | Ame.KB882086 |  |
| Ciona intestinalis | | 2: 1207.59k | ENSCING00000011599 | ENSCINT00000022340.2 | Cin.2 |  |
| Ciona savignyi | | reftig_19: 2.37m | ENSCSAVG00000009237 | ENSCSAVT00000015908.1 | Csa.ref19 |  |
| Fruitfly | RhoGAP68F | 3L: 12.14m | FBgn0036257 | FBtr0075997 | Dme.3L |  |
| Caenorhabditis elegans | | II: 11.46m | WBGene00012203 | W02B12.8a | Cel.II |  |
|  |  |  |  |  |  |  |
| **Species** | **HGNC,ZFIN or FlyBase name** | **Chromosome/scaffold locations** | **Ensembl ID or NCBI accession number** | **Transcript ID** | **Assigned sequence name** | **Comments/Annotation notes** |
| Human | CREB3L1 | 11: 46.28m | ENSG00000157613 | ENST00000621158.4 | Hsa.11 |  |
|  | CREB3L2 | 7: 137.87m | ENSG00000182158 | ENST00000330387.10 | Hsa.7 |  |
| Chicken |  | 5: 23.70m | ENSGALG00000008393 | ENSGALT00000013673.3 | Gga.5 |  |
|  |  | 1: 57.41m | ENSGALG00000012877 | ENSGALT00000021004.5 | Gga.1 |  |
| Coelacanth | | JH126568.1: 3.62m | ENSLACG00000018569 | ENSLACT00000021277.1 | Lch.JH126568 |  |
|  |  | JH126628.1: 350.69k | ENSLACG00000008535 | ENSLACT00000009754.1 | Lch.JH126628 |  |
| Spotted gar | | LG27: 12.11m | ENSLOCG00000005600 | ENSLOCT00000006770.1 | Loc.LG27 |  |
|  |  | LG8: 49.54m | ENSLOCG00000017109 | ENSLOCT00000021177.1 | Loc.LG8 | manually edited |
| Zebrafish | creb3l1 | 7: 38.63m | ENSDARG00000015793 | ENSDART00000172251.1 | Dre.7 |  |
|  | creb3l2 | 4: 4.70m | ENSDARG00000063563 | ENSDART00000092984.4 | Dre.4 |  |
| Ciona intestinalis | | Scaffold HT000025.1: 3.65k | ENSCING00000005364 | ENSCINT00000011026.3 | Cin.HT000025 |  |
| Ciona savignyi | | reftig_19: 1732.56k | ENSCSAVG00000008562 | ENSCSAVT00000014819.1 | Csa.ref19 |  |
| Fruitfly | CrebA | 3L: 15.54m | FBgn0004396 | FBtr0075557 | Dme.3L |  |
| Caenorhabditis elegans | | II: 5.18m | WBGene00016162 | C27D6.4c | Cel.II |  |
|  |  |  |  |  |  |  |
| **Species** | **HGNC,ZFIN or FlyBase name** | **Chromosome/scaffold locations** | **Ensembl ID or NCBI accession number** | **Comments/Annotation notes** |  |  |
| Human | CRY1 | 12: 106.99m | ENSG00000008405 |  |  |  |
|  | CRY2 | 11: 45.85m | ENSG00000121671 |  |  |  |
| Chicken |  | 1: 53.64m | ENSGALG00000012638 |  |  |  |
|  |  | 5: 23.95m | ENSGALG00000008436 |  |  |  |
| Spotted gar | | JH591436.1: 96.77k | ENSLOCG00000014655 |  |  |  |
|  |  | LG3: 32.90m | ENSLOCG00000011417 | Assigned gene name in synteny figure: CRY3 |  |  |
|  |  | LG8: 4.05m | ENSLOCG00000015272 |  |  |  |
| Zebrafish | cry2 | 25: 13.75m | ENSDARG00000102403 |  |  |  |
|  | cry1ba | 8: 21.19m | ENSDARG00000069074 | Assigned gene name in this study: cry3a |  |  |
|  | cry1bb | 22: 748.82k | ENSDARG00000091131 | Assigned gene name in this study: cry3b |  |  |
|  | cry1aa | 4: 12.01m | ENSDARG00000045768 | Assigned gene name in this study: cry1a |  |  |
|  | cry1ab | 18: 15.14m | ENSDARG00000011583 | Assigned gene name in this study: cry1b |  |  |
|  |  |  |  |  |  |  |
| Reference for phylogenetic analysis and assigned gene names of the CRY family: | | |  |  |  |  |
| Haug, M.F., Gesemann, M., Lazović, V., and Neuhauss, S.C.F. (2015). Eumetazoan Cryptochrome Phylogeny and Evolution. Genome Biol Evol *7*, 601–619 | | | | | |  |
|  |  |  |  |  |  |  |
| **Species** | **HGNC,ZFIN or FlyBase name** | **Chromosome/scaffold locations** | **Ensembl ID or NCBI accession number** | **Transcript ID** | **Assigned sequence name** | **Comments/Annotation notes** |
| Human | DGKI | 7: 137.38m | ENSG00000157680 | ENST00000288490.9 | Hsa.7 |  |
|  | DGKZ | 11: 46.33m | ENSG00000149091 | ENST00000454345.5 | Hsa.11 |  |
| Chicken |  | 1: 57.49m | ENSGALG00000012890 | ENSGALT00000021020.5 | Gga.1 |  |
|  |  | 5: 23.65m | ENSGALG00000008380 | ENSGALT00000038374.3 | Gga.5 |  |
| Coelacanth | | JH126568.1: 4.02m | ENSLACG00000018674 | ENSLACT00000021394.1 | Lch.JH126568 |  |
|  |  | JH126628.1: 638.23k | ENSLACG00000011803 | ENSLACT00000013500.1 | Lch.JH126628 | manually edited |
| Spotted gar | | LG8: 49.59m | ENSLOCG00000017111 | ENSLOCT00000021183.1 | Loc.LG8 | manually edited |
|  |  | LG27: 11.92m | ENSLOCG00000005570 | ENSLOCT00000006752.1 | Loc.LG27 |  |
| Zebrafish | dgki | 4: 4.61m | ENSDARG00000063578 | ENSDART00000093005.5 | Dre.4 |  |
|  | dgkza | 7: 38.69m | ENSDARG00000014439 | ENSDART00000093009.5 | Dre.7 |  |
|  | dgkzb | 25: 7.66m | ENSDARG00000076025 | ENSDART00000155016.1 | Dre.25 |  |
| Ciona intestinalis | | 14: 2.60m | ENSCING00000003441 | ENSCINT00000007047.3 | Cin.14 |  |
|  |  | 4: 3.98m | ENSCING00000008888 | ENSCINT00000018087.3 | Cin.4 |  |
| Ciona savignyi | | reftig_76: 1870.70k | ENSCSAVG00000008009 | ENSCSAVT00000013813.1 | Csa.ref76 |  |
|  |  | reftig_90: 179.73k | ENSCSAVG00000009376 | ENSCSAVT00000016110.1 | Csa.ref90 |  |
| Fruitfly | rdgA | X: 8.91m | FBgn0261549 | FBtr0302660 | Dme.X |  |
| Caenorhabditis elegans | | II: 6.46m | WBGene00019428 | K06A1.6 | Cel.II |  |
|  |  |  |  |  |  |  |
| **Species** | **HGNC,ZFIN or FlyBase name** | **Chromosome/scaffold locations** | **Ensembl ID or NCBI accession number** | **Transcript ID** | **Assigned sequence name** | **Comments/Annotation notes** |
| Human | MYBPC1 | 12: 101.57m | ENSG00000196091 | ENST00000361466.6 | Hsa.12 |  |
|  | MYBPHL | 1: 109.29m | ENSG00000221986 | ENST00000357155.1 | Hsa.1.1 |  |
|  | MYBPC3 | 11: 47.33m | ENSG00000134571 | ENST00000545968.5 | Hsa.11 |  |
|  | MYBPC2 | 19: 50.43m | ENSG00000086967 | ENST00000357701.5 | Hsa.19 |  |
|  | MYBPH | 1: 203.17m | ENSG00000133055 | ENST00000255416.8 | Hsa.1.2 |  |
| Chicken |  | 1:55.65m | XP_015143170.1 |  | Gga.1 | jan-16 |
|  |  | 26: 1001.41k | ENSGALG00000000164 | ENSGALT00000040700.4 | Gga.26 |  |
|  |  | 5: 22.98m | ENSGALG00000008148 | ENSGALT00000032650.4 | Gga.5 |  |
| Anole lizard | | 6: 79.59m | ENSACAG00000012342 | ENSACAT00000012500.3 | Aca.6 |  |
|  |  | 1: 43.90m | XP_008103421.2 |  | Aca.1 | may-16 |
|  |  | 4: 133.15m | ENSACAG00000004704 | ENSACAT00000004779.2 | Aca.4 |  |
|  |  | 5:17.24m | XP_008108767.1 |  | Aca.5 | may-16 |
| Coelacanth | | JH127380.1: 83.40k | ENSLACG00000003430 | ENSLACT00000003888.1 | Lch.JH127380 |  |
|  |  | JH126576.1: 821.40k | ENSLACG00000013194 | ENSLACT00000015097.1 | Lch.JH126576 |  |
|  |  | JH126568.1: 5.51m | ENSLACG00000018941 | ENSLACT00000021699.1 | Lch.JH126568 |  |
|  |  | JH126644.1: 1323.14k | ENSLACG00000015666 | ENSLACT00000017917.1 | Lch.JH126644 |  |
| Spotted gar |  | *JH591442.1: 155.01k* | *ENSLOCG00000000775* | *ENSLOCT00000000861.1* |  | *too short not included in alignment* |
|  |  | LG27: 12.85m | ENSLOCG00000006281 | ENSLOCT00000007615.1 | Loc.LG27 |  |
|  |  | LG3: 23.56m | ENSLOCG00000009756 | ENSLOCT00000011949.1 | Loc.LG3 |  |
|  |  | LG8: 2.64m | ENSLOCG00000015188 | ENSLOCT00000018724.1 | Loc.LG8 |  |
| Zebrafish | mybpc1 | 4: 17.74m | ENSDARG00000045560 | ENSDART00000066996.4 | Dre.4 |  |
|  | mybpha | 23: 6.14m | ENSDARG00000058799 | ENSDART00000139834.1 | Dre.23 |  |
|  | mybphb | 6: 55.02m | ENSDARG00000003081 | ENSDART00000122794.2 | Dre.6 |  |
|  | mybpc3 | 7: 31.57m | ENSDARG00000011615 | ENSDART00000174217.1 | Dre.7 |  |
|  | mybpc2a | 3: 30.13m | ENSDARG00000030157 | ENSDART00000121883.3 | Dre.3 |  |
|  | mybpc2b | 24: 38.37m | ENSDARG00000021265 | ENSDART00000105672.4 | Dre.24 |  |
| Ciona intestinalis | | 1: 2.00m | ENSCING00000004377 | ENSCINT00000009061.3 | Cin.1 |  |
| Ciona savignyi | | reftig_14: 840.05k | ENSCSAVG00000010522 | ENSCSAVT00000018078.1 | Csa.ref14 |  |
| Fruitfly | CG14964 | 3L: 3.17m | FBgn0035410 | FBtr0307018 | Dme.3L |  |
| Human | IGSF22 | 11: 18.70m | ENSG00000179057 | ENST00000513874.5 | Hsa.IGSF22 |  |
|  | IGFN1 | 1: 201.19m | ENSG00000163395 | ENST00000295591.12 | Hsa.IGFN1 |  |
|  | MYOM1 | 18: 3.07m | ENSG00000101605 | ENST00000356443.8 | Hsa.MYOM1 |  |
|  | MYOM2 | 8: 2.05m | ENSG00000036448 | ENST00000262113.8 | Hsa.MYOM2 |  |
|  | MYOM3 | 1: 24.06m | ENSG00000142661 | ENST00000374434.3 | Hsa.MYOM3 |  |
| Amphioxus |  | NW_003101518.1: 2.24m | XP_002605775.1 |  | Bfl.NW_003101518 | oct-09 |
|  |  |  |  |  |  |  |
| **Species** | **HGNC,ZFIN or FlyBase name** | **Chromosome/scaffold locations** | **Ensembl ID or NCBI accession number** | **Transcript ID** | **Assigned sequence name** | **Comments/Annotation notes** |
| Human | NAV1 | 1: 201.62m | ENSG00000134369 | ENST00000367296.8 | Hsa.1 |  |
|  | NAV2 | 11: 19.35m | ENSG00000166833 | ENST00000349880.8 | Hsa.11 |  |
|  | NAV3 | 12: 77.32m | ENSG00000067798 | ENST00000397909.6 | Hsa.12 |  |
| Chicken |  | 5:1.70m | XP_004941374.1 |  | Gga.5 | jan-16 |
|  |  | 1: 38.67m | ENSGALG00000010311 | ENSGALT00000016778.5 | Gga.1 |  |
|  |  | 26:0.61m | XP_015154353.1 |  | Gga.26 | jan-16 |
| Coelacanth | | NW_005819268.1:0.78m | XP_014343595.1 |  | Lch.NW_005819268 | oct-15 |
|  |  | NW_005819385.1:0.32m | XP_014344647.1 |  | Lch.NW_005819385 | oct-15 |
|  |  | JH127285.1: 11.60k | ENSLACG00000001187 | ENSLACT00000001335.1 | Lch.JH127285 |  |
| Spotted gar | | LG3: 23.60m | ENSLOCG00000009769 | ENSLOCT00000011965.1 | Loc.LG3 |  |
|  |  | LG27: 10.09m | ENSLOCG00000004849 | ENSLOCT00000005843.1 | Loc.LG27 |  |
|  |  | LG8: 29.60m | ENSLOCG00000016281 | ENSLOCT00000020119.1 | Loc.LG8 |  |
| Zebrafish | nav1b | 6: 54.87m | ENSDARG00000058771 | ENSDART00000113331.3 | Dre.6 |  |
|  |  | 23: 5.94m | ENSDARG00000078011 | ENSDART00000115403.3 | Dre.23 |  |
|  | nav2a | 7: 16.55m | ENSDARG00000073688 | ENSDART00000173541.1 | Dre.7 |  |
|  | nav2b | 25: 14.41m | ENSDARG00000001879 | ENSDART00000160462.1 | Dre.25 |  |
|  | nav3 | 4: 21.09m | ENSDARG00000005476 | ENSDART00000005847.8 | Dre.4 |  |
| Amphioxus |  | NW_003101540.1: 220.56m |  | XP_002609491.1 | Bfl.NW_003101540 | oct-09, manually edited |
|  |  | NW_003101540.1: 232.81m |  | XP_002609492.1 |  | oct-09, manually edited |
| Fruitfly | sick | 2L: 19.80m | FBgn0263873 | FBtr0329845 | Dme.2L |  |
| Caenorhabditis elegans | | II: 11.06m | WBGene00006788 | F45E10.1c | Cel.II |  |
|  |  |  |  |  |  |  |
| **Species** | **HGNC,ZFIN or FlyBase name** | **Chromosome/scaffold locations** | **Ensembl ID or NCBI accession number** | **Transcript ID** | **Assigned sequence name** | **Comments/Annotation notes** |
| Human | NELL1 | 11: 20.67m | ENSG00000165973 | ENST00000357134.9 | Hsa.11 |  |
|  | NELL2 | 12: 44.51m | ENSG00000184613 | ENST00000429094.6 | Hsa.12 |  |
| Chicken |  | 1: 30.34m | ENSGALG00000009601 | ENSGALT00000038787.4 | Gga.1 |  |
|  |  | 5: 2.20m | ENSGALG00000003777 | ENSGALT00000005993.5 | Gga.5 |  |
| Coelacanth | | JH126716.1: 1989.65k | ENSLACG00000017255 | ENSLACT00000019760.1 | Lch.JH126716 |  |
|  |  | JH126936.1: 904.27k | ENSLACG00000013745 | ENSLACT00000015720.1 | Lch.JH126936 | manually edited |
|  |  | JH126936.1: 1162.57k | ENSLACG00000015057 | ENSLACT00000017211.1 |  | manually edited |
| Spotted gar | | LG27: 10.35m | ENSLOCG00000004971 | ENSLOCT00000006006.1 | Loc.LG27 |  |
|  |  | LG8: 35.39m | ENSLOCG00000016436 | ENSLOCT00000020336.1 | Loc.LG8 |  |
| Zebrafish | nell2a | 25: 558.24k | ENSDARG00000090690 | ENSDART00000126863.2 | Dre.25 |  |
|  | nell2b | 4: 14.25m | ENSDARG00000062797 | ENSDART00000091151.5 | Dre.4 |  |
| Stickleback | | groupII: 20.13m | ENSGACG00000017098 | ENSGACT00000022628.1 | Gac.II |  |
|  |  | groupXIX: 5.40m | ENSGACG00000004004 | ENSGACT00000005280.1 | Gac.XIX |  |
| Amphioxus |  | NW_017803998.1:2.57m | XP_019633163.1 |  | Bfl.NW_017803998 |  |
| Caenorhabditis elegans | | X: 10.01m | WBGene00008952 | F19C6.3 | Cel.X |  |
|  |  |  |  |  |  |  |
| **Species** | **HGNC,ZFIN or FlyBase name** | **Chromosome/scaffold locations** | **Ensembl ID or NCBI accession number** | **Transcript ID** | **Assigned sequence name** | **Comments/Annotation notes** |
| Human | PPFIA1 | 11: 70.27m | ENSG00000131626 | ENST00000253925.11 | Hsa.11 |  |
|  | PPFIA2 | 12: 81.26m | ENSG00000139220 | ENST00000549396.5 | Hsa.12 |  |
|  | PPFIA3 | 19: 49.12m | ENSG00000177380 | ENST00000334186.8 | Hsa.19 |  |
|  | PPFIA4 | 1: 203.03m | ENSG00000143847 | ENST00000367240.6 | Hsa.1 |  |
| Chicken |  | 5: 17.84m | ENSGALG00000035774 | ENSGALT00000053972.1 | Gga.5 |  |
|  |  | 1: 40.20m | ENSGALG00000010958 | ENSGALT00000054646.1 | Gga.1 |  |
|  |  | 26: 944.39k | ENSGALG00000000217 | ENSGALT00000000287.5 | Gga.26 |  |
| Anole lizard | | GL343283.1: 0.11m | ENSACAG00000003711 | ENSACAT00000003896.3 | Aca.GL343284 |  |
|  |  | 4: 133.33m | ENSACAG00000004833 | ENSACAT00000004914.3 | Aca.4 |  |
|  |  | 5: 36.50m | ENSACAG00000014550 | ENSACAT00000014705.3 | Aca.5 |  |
|  |  | 6: 79.91m | ENSACAG00000013088 | ENSACAT00000013249.3 | Aca.6 |  |
| Coelacanth | | JH126862.1: 275.87k | ENSLACG00000007439 | ENSLACT00000008467.1 | Lch.JH126862 |  |
|  |  | JH126851.1: 904.80k | ENSLACG00000013750 | ENSLACT00000015725.1 | Lch.JH126851 |  |
|  |  | JH130138.1: 52.91k | ENSLACG00000002574 | ENSLACT00000002904.1 | Lch.JH130138 |  |
|  |  | JH126576.1: 1128.03k | ENSLACG00000014912 | ENSLACT00000017049.1 | Lch.JH126576 |  |
| Spotted gar | | LG27: 11.09m | ENSLOCG00000005197 | ENSLOCT00000006281.1 | Loc.LG27 |  |
|  |  | LG8: 30.86m | ENSLOCG00000016312 | ENSLOCT00000020174.1 | Loc.LG8 |  |
|  |  | LG3: 29.56m | ENSLOCG00000010689 | ENSLOCT00000013142.1 | Loc.LG3 |  |
| Zebrafish | ppfia3 | 3: 29.91m | ENSDARG00000077053 | ENSDART00000153562.2 | Dre.3 |  |
|  | ppfia1 | 18:50.94m | NP_001104714.1 |  | Dre.18 |  |
|  | ppfia4 | 11: 22.21m | ENSDARG00000053205 | ENSDART00000112567.3 | Dre.11 |  |
|  | ppfia2 | 4: 22.24m | ENSDARG00000013000 | ENSDART00000142140.1 | Dre.4 |  |
| Ciona intestinalis | | Scaffold HT000098.1: 459.49k | ENSCING00000006030 | ENSCINT00000012503.3 | Cin.HT000098 |  |
| Ciona savignyi | | reftig_489: 160.55k | ENSCSAVG00000001250 | ENSCSAVT00000002163.1 | Csa.ref489 |  |
| Fruitfly | Liprin-alpha | 2L: 6.72m | FBgn0046704 | FBtr0329943 | Dme.2L |  |
| Caenorhabditis elegans | | X: 10.55m | WBGene00006364 | F59F5.6 | Cel.X |  |
|  |  |  |  |  |  |  |
| **Species** | **HGNC,ZFIN or FlyBase name** | **Chromosome/scaffold locations** | **Ensembl ID or NCBI accession number** | **Transcript ID** | **Assigned sequence name** | **Comments/Annotation notes** |
| Human | RASSF7 | 11: 560.40k | ENSG00000099849 | ENST00000397583.7 | Hsa.11 |  |
|  | RASSF8 | 12: 25.96m | ENSG00000123094 | ENST00000405154.6 | Hsa.12 |  |
| Chicken |  | 1:67.55m | XP_416437.2 |  | Gga.1 | jan-16 |
|  |  | 5: 15.89m | ENSGALG00000044251 | ENSGALT00000011134.6 | Gga.5 |  |
| Anole lizard | | GL343349: 1.01m | ENSACAG00000000388 | ENSACAT00000000374.3 | Aca.GL343349 |  |
|  |  | 1: 74.53m | ENSACAG00000014995 | ENSACAT00000015019.3 | Aca.1 |  |
| Coelacanth | | JH126587.1: 3.31m | ENSLACG00000018442 | ENSLACT00000021129.1 | Lch.JH126587 | assigned family member name in tree: RASSF11 |
|  |  | JH126580.1: 2.92m | ENSLACG00000018230 | ENSLACT00000020892.2 | Lch.JH126580 |  |
|  |  | JH127283.1: 859.80k | ENSLACG00000013466 | ENSLACT00000025618.1 | Lch.JH127283 |  |
| Spotted gar | | LG8: 4.77m | ENSLOCG00000015329 | ENSLOCT00000018894.1 | Loc.LG8 |  |
|  |  | LG27: 68.92k | ENSLOCG00000000536 | ENSLOCT00000000636.1 | Loc.LG27 |  |
|  |  | LG3: 28.57m | ENSLOCG00000010433 | ENSLOCT00000012814.1 | Loc.LG3 | assigned family member name in tree: RASSF11 |
| Zebrafish | si:ch211-261n11.3 | 8:22.52m | XP_021334229.1 |  | Dre.8 | sep -17 , assigned gene name in this study:rassf11 |
|  | rassf8b | 4: 20.79m | ENSDARG00000045485 | ENSDART00000066895.3 | Dre.4 |  |
|  | rassf8a | 25: 31.05m | ENSDARG00000045596 | ENSDART00000067039.4 | Dre.25.2 |  |
|  | rassf7a | 25: 25.36m | ENSDARG00000079917 | ENSDART00000112330.3 | Dre.25.1 |  |
|  | rassf7b | 7: 49.38m | ENSDARG00000003193 | ENSDART00000025451.6 | Dre.7 |  |
| Ciona intestinalis | | Scaffold HT000075.1: 354.35k | ENSCING00000007050 | ENSCINT00000014493.3 | Cin.HT000075 |  |
| Fruitfly | RASSF8 | 3R: 25.66m | FBgn0261986 | FBtr0084898 | Dme.3R |  |
| Caenorhabditis elegans | | X: 4.91m | WBGene00019403 | K05B2.2a | Cel.X |  |
|  |  |  |  |  |  |  |
| **Species** | **HGNC,ZFIN or FlyBase name** | **Chromosome/scaffold locations** | **Ensembl ID or NCBI accession number** | **Transcript ID** | **Assigned sequence name** | **Comments/Annotation notes** |
| Human | SHANK1 | 19: 50.66m | ENSG00000161681 | ENST00000293441.5 | Hsa.19 |  |
|  | SHANK2 | 11: 70.47m | ENSG00000162105 | ENST00000601538.5 | Hsa.11 |  |
|  | SHANK3 | 22: 50.67m | ENSG00000251322 | ENST00000445220.5 | Hsa.22 |  |
| Chicken |  | 1: 393.74k | ENSGALG00000039165 | ENSGALT00000052302.1 | Gga.1 | manually edited |
|  |  | 1: 155.62k | ENSGALG00000033919 | ENSGALT00000080172.1 |  | manually edited |
|  |  | 5:17.92m | XP_015142368.1 |  | Gga.5 | jan-16 |
| Anole lizard | | 6: 79.30m | ENSACAG00000012124 | ENSACAT00000012209.3 | Aca.6 |  |
|  |  | NW_003338830.1:0.24m | XP_008115586.1 |  | Aca.NW_003338830 |  |
|  |  | GL343837.1: 74.61k | ENSACAG00000000095 | ENSACAT00000027586.2 | Aca.GL343837 |  |
| Coelacanth | | JH126644.1: 84.57k | ENSLACG00000003467 | ENSLACT00000003926.1 | Lch.JH126644 |  |
|  |  | JH126862.1: 138.17k | ENSLACG00000004766 | ENSLACT00000005408.1 | Lch.JH126862 |  |
|  |  | JH128886.1: 115.38k | ENSLACG00000004253 | ENSLACT00000004825.1 | Lch.JH128886 |  |
| Spotted gar | | LG27: 11.16m | ENSLOCG00000005253 | ENSLOCT00000006340.1 | Loc.LG27 |  |
|  |  | LG8:43.08m | XP_015208638.1 |  | Loc.LG8 | jan-16 |
| Zebrafish | shank3b | 4: 9.55m | ENSDARG00000063054 | ENSDART00000145613.2 | Dre.4 |  |
|  | shank3a | 18:7.66m | XP_017207498.1 |  | Dre.18.1 | jun-17 |
|  | shank1 | 3:30.70m | XP_021329733.1 |  | Dre.3 | jun-17 |
|  | shank2 | 25: 14.07m | ENSDARG00000062325 | ENSDART00000145387.1 | Dre.25 | Assigned gene name in this study: shank2b |
|  | si:ch1073-450f2.1 | 18: 50.97m | ENSDARG00000102443 | ENSDART00000174109.1 | Dre.18.2 | Assigned gene name in this study: shank2a |
| Amphioxus |  | NW_003101532.1:2.80m | XP_002607814.1 |  | Bfl.NW_003101532 | oct-09 |
| Fruitfly | Prosap | 2R: 14.06m | FBgn0040752 | FBtr0087601 | Dme.2R |  |
| Caenorhabditis elegans | | II: 11.38m | WBGene00006444 | C33B4.3c | Cel.II |  |
