# Supplementary material for: Evolution of the Muscarinic Acetylcholine Receptors in Vertebrates
Source: eNeuro. 2018 Nov 8;5(5):ENEURO.0340-18.2018. doi: 10.1523/ENEURO.0340-18.2018 (PMC6298421; doi:10.1523/ENEURO.0340-18.2018)
Supplement: Figure 3-3 — Information about the neighboring gene family sequences included in the analysis of the genomic regions surrounding the CHRM1, CHRM3, and CHRM5 genes. First, the genome assembly versions used are stated, followed by information about the neighbor gene families included in the analysis in the following order: species, HGNC/ZFIN/Flybase symbol name, chromosome or genomic scaffold position, Ensembl ID or NCBI accession number, assigned sequence name, and additional comments regarding sequence update date on NCBI or whether there have been manual edits of the original Ensembl or NCBI sequence. Download Fig. 3-3, DOCX file. [file sup_enu-eN-NWR-0340-18-s10.docx]

| **Ens87** | **Common name** | **Scientific name** | **Abbreviation** | **Assembly Ensembl** | **Assembly NCBI** |  |
| --- | --- | --- | --- | --- | --- | --- |
|  | Anole lizard | Anolis carolinensis | Aca | AnoCar2.0 | AnoCar2.0 |  |
|  | Amphioxus | Branchiostoma floridae | Bfl | GCA_000003815.1 Version 2 |  |  |
|  | Ciona intestinalis | Ciona intestinalis | Cin | KH |  |  |
|  | Ciona savignyi | Ciona savignyi | Csa | CSAV 2.0 |  |  |
|  | Caenorhabditis elegans | Caenorhabditis elegans | Cel | WBcel235 |  |  |
|  | Chicken | Gallus gallus | Gga | Gallus_gallus-5.0 |  |  |
|  | Chinese softshell turtle | Pelodiscus sinensis | Psi | PelSin_1.0 |  |  |
|  | Coelacanth | Latimeria chalumnae | Lch | LatCha1 | LatCha1 |  |
|  | Fruitfly | Drosophila melanogaster | Dme | BDGP6 |  |  |
|  | Human | Homo sapiens | Hsa | GRCh38.p7 |  |  |
|  | Medaka | Oryzias latipes | Ola | HdrR |  |  |
|  | Purple sea urchin | Strongylocentrotus purpuratus | Spu | Spur_4.2 |  |  |
|  | Spotted gar | Lepisosteus oculatus | Loc | LepOcu1 | LepOcu1 |  |
|  | Zebrafish | Danio rerio | Dre | GRCz10 | GRCz11 |  |
|  |  |  |  |  |  |  |
| **Species** | **HGNC,ZFIN or FlyBase name** | **Chromosome/scaffold locations** | **Ensembl ID or NCBI accession number** | **Transcript ID** | **Assigned sequence name** | **Comments/Annotation notes** |
| Human | ATL1 | 14: 50.53m | ENSG00000198513 | ENST00000441560.6 | Hsa.14 |  |
|  | ATL2 | 2: 38.29m | ENSG00000119787 | ENST00000378954.8 | Hsa.2 |  |
|  | ATL3 | 11: 63.62m | ENSG00000184743 | ENST00000398868.7 | Hsa.11 |  |
| Chicken |  | 5: 58.00m | ENSGALG00000012339 | ENSGALT00000020162.5 | Gga.5 |  |
|  |  | 3: 16.43m | ENSGALG00000037289 | ENSGALT00000065464.1 | Gga.3 |  |
| Anole lizard |  | Scaffold GL343890.1: 68.89k | ENSACAG00000002701 | ENSACAT00000002836.3 | Aca.GL343890 |  |
|  |  | 1: 254.16m | ENSACAG00000014506 | ENSACAT00000014651.3 | Aca.1 |  |
| Coelacanth | | Scaffold JH126849.1: 676.04k | ENSLACG00000012098 | ENSLACT00000013840.1 | Lch.JH126849 |  |
|  |  | Scaffold JH126593.1: 88.70k | ENSLACG00000003585 | ENSLACT00000004062.2 | Lch.JH126593 |  |
| Spotted gar | | LG7: 12.07m | ENSLOCG00000011885 | ENSLOCT00000014646.1 | Loc.LG7 |  |
|  |  | LG16: 4.55m | ENSLOCG00000015787 | ENSLOCT00000019475.1 | Loc.LG16 |  |
|  |  | LG28: 118.01k | ENSLOCG00000000849 | ENSLOCT00000000956.1 | Loc.LG28 |  |
| Zebrafish | atl1 | 13: 36.64m | ENSDARG00000060481 | ENSDART00000111832.4 | Dre.13.2 |  |
|  | atl2 | 13: 7.95m | ENSDARG00000057719 | ENSDART00000080460.6 | Dre.13.1 |  |
|  | atl3 | 14: 30.43m | ENSDARG00000004270 | ENSDART00000023054.9 | Dre.14 |  |
| Amphioxus | | NW_003101444.1:1.84m | XP_002596876.1 |  | Bfl.NW_003101444 | oct-09 |
| Fruitfly | atl | 3R: 24.63m | FBgn0039213 | FBtr0084657 | Dme.3R |  |
| Caenorhabditis elegans | | IV: 2.79m | WBGene00021868 | Y54G2A.2b | Cel.IV |  |
|  |  |  |  |  |  |  |
| **Species** | **HGNC,ZFIN or FlyBase name** | **Chromosome/scaffold locations** | **Ensembl ID or NCBI accession number** | **Transcript ID** | **Assigned sequence name** | **Comments/Annotation notes** |
| Human | EHD1 | 11: 64.85m | ENSG00000110047 | ENST00000320631.7 | Hsa.11 |  |
|  | EHD2 | 19: 47.71m | ENSG00000024422 | ENST00000263277.7 | Hsa.19 |  |
|  | EHD3 | 2: 31.23m | ENSG00000013016 | ENST00000322054.9 | Hsa.2 |  |
|  | EHD4 | 15: 41.90m | ENSG00000103966 | ENST00000220325.8 | Hsa.15 |  |
| Chicken |  | Scaffold NT_465842.1:0.05k | XP_015129283.1 |  | Gga.scaffold | jan-16, manually edited |
|  |  | 3: 7.72m | ENSGALG00000009086 | ENSGALT00000014784.3 | Gga.3 |  |
|  |  | 5: 25.30m | ENSGALG00000008950 | ENSGALT00000014555.4 | Gga.5 |  |
| Anole lizard |  | NW_003342544.1:0.02m | XP_003230702.2 |  | Aca.NW_003342544 | may-16 |
|  |  | 1: 40.55m | ENSACAG00000014864 | ENSACAT00000014952.3 | Aca.1.1 |  |
|  |  | 1: 250.59m | ENSACAG00000007103 | ENSACAT00000007133.2 | Aca.1.2 |  |
|  |  | NW_003339612.1 :0.09m | XP_003229733.2 |  | Aca.NW_003339612 | may-16 |
| Chinese softshell turtle |  | Scaffold JH212639.1: 172.05k | ENSPSIG00000005727 | ENSPSIT00000006245.1 | Psi.JH212639 |  |
|  |  | Scaffold JH207369.1: 2.77m | ENSPSIG00000017757 | ENSPSIT00000020163.1 | Psi.JH207369 |  |
|  |  | Scaffold JH210682.1: 460.57k | ENSPSIG00000016990 | ENSPSIT00000019278.1 | Psi.JH210682 |  |
| Coelacanth | | Scaffold JH126593.1: 2.81m | ENSLACG00000018148 | ENSLACT00000020794.1 | Lch.Jh126593 | manually edited |
|  |  | Scaffold JH126578.1: 3.07m | ENSLACG00000018332 | ENSLACT00000021006.1 | Lch.JH126578 | manually edited |
|  |  | Scaffold JH128897.1: 108.39k | ENSLACG00000004089 | ENSLACT00000004631.1 | Lch.JH128897 | manually edited |
|  |  | Scaffold JH126639.1: 2.39m | ENSLACG00000017804 | ENSLACT00000020398.1 | Lch.JH126639 | manually edited |
| Spotted Gar | | LG28: 160.66k | ENSLOCG00000000924 | ENSLOCT00000001034.1 | Loc.LG28 | manually edited |
|  |  | LG2: 62.19m | ENSLOCG00000014200 | ENSLOCT00000017525.1 | Loc.LG2 | manually edited |
|  |  | LG1: 46.26m | ENSLOCG00000016786 | ENSLOCT00000020785.1 | Loc.LG1 |  |
|  |  | LG7: 6.14m | ENSLOCG00000009774 | ENSLOCT00000011963.1 | Loc.LG7 |  |
| Zebrafish | ehd1a | 14: 46.45m | ENSDARG00000098853 | ENSDART00000173209.1 | Dre.14 |  |
|  | ehd1b | 10: 27.11m | ENSDARG00000014793 | ENSDART00000012717.7 | Dre.10 |  |
|  | ehd2a | 5: 61.31m | ENSDARG00000035137 | ENSDART00000050885.4 | Dre.5 |  |
|  | ehd2b | 18: 44.66m | ENSDARG00000040362 | ENSDART00000059063.3 | Dre.18 |  |
|  | ehd3 | 20: 38.30m | ENSDARG00000007869 | ENSDART00000022694.6 | Dre.20 |  |
|  | ehd4 | 17:29.19m | XP_698033.4 |  | Dre.17 | jun-17 |
| Ciona intestinalis |  | 2: 2.68m | ENSCING00000005231 | ENSCINT00000010754.3 | Cin.2 |  |
| Ciona savignyi |  | reftig_219: 187.05k | ENSCSAVG00000000028 | ENSCSAVT00000000059.1 | Csa.ref219 |  |
| Caenorhabditis elegans | | V: 6.21m | WBGene00004373 | W06H8.1a | Cel.V |  |
| Fruitfly | Past1 | 3R: 12.70m | FBgn0016693 | FBtr0082654 | Dme.3R |  |
|  |  |  |  |  |  |  |
| **Species** | **HGNC,ZFIN or FlyBase name** | **Chromosome/scaffold locations** | **Ensembl ID or NCBI accession number** | **Transcript ID** | **Assigned sequence name** | **Comments/Annotation notes** |
| Human | FERMT1 | 20: 6.07m | ENSG00000101311 | ENST00000217289.8 | Hsa.20 |  |
|  | FERMT2 | 14: 52.86m | ENSG00000073712 | ENST00000343279.8 | Hsa.14 |  |
|  | FERMT3 | 11: 64.21m | ENSG00000149781 | ENST00000279227.9 | Hsa.11 |  |
| Chicken |  | 3: 15.31m | ENSGALG00000008827 | ENSGALT00000014344.5 | Gga.3 |  |
|  |  | 5: 58.40m | ENSGALG00000012425 | ENSGALT00000020309.5 | Gga.5 |  |
| Chinese softshell  turtle | | Scaffold JH210532.1: 49.20k | ENSPSIG00000008127 | ENSPSIT00000009189.1 | Psi.JH210532 |  |
|  |  | Scaffold JH206845.1: 2.39m | ENSPSIG00000014143 | ENSPSIT00000016041.1 | Psi.JH206845 |  |
|  |  | Scaffold JH212622.1: 456.01k | ENSPSIG00000005395 | ENSPSIT00000005845.1 | Psi.2JH12622 |  |
| Coelacanth | | Scaffold JH126648.1: 1445.85k | ENSLACG00000016076 | ENSLACT00000018382.1 | Lch.JH126648 |  |
|  |  | Scaffold JH126564.1: 7.75m | ENSLACG00000019103 | ENSLACT00000021880.1 | Lch.JH126564 |  |
|  |  | Scaffold JH126593.1: 1785.83k | ENSLACG00000016877 | ENSLACT00000019317.1 | Lch.JH126593 |  |
| Spotted gar | | LG1: 34.61m | ENSLOCG00000016445 | ENSLOCT00000020347.1 | Loc.LG1 |  |
|  |  | LG7: 12.89m | ENSLOCG00000012153 | ENSLOCT00000014982.1 | Loc.LG7 |  |
|  |  | LG28: 537.85k | ENSLOCG00000001364 | ENSLOCT00000001550.1 | Loc.LG28 | manually edited |
| Zebrafish | fermt1 | 20: 45.87m | ENSDARG00000052652 | ENSDART00000074546.5 | Dre.20 |  |
|  | fermt2 | 17: 50.62m | ENSDARG00000020242 | ENSDART00000049464.7 | Dre.17 |  |
|  | fermt3a | 5: 66.08m | ENSDARG00000079267 | ENSDART00000113077.3 | Dre.5 |  |
|  | fermt3b | 14: 46.92m | ENSDARG00000030938 | ENSDART00000047716.6 | Dre.14 |  |
| Ciona intestinalis |  | 1:8.43m | XP_009862486.1 |  | Cin.1 | oct-16 |
| Fruitfly | Fit2 | 3L: 17.02m | FBgn0036688 | FBtr0075275 | Dme.3L.2 |  |
|  | Fit1 | 3L: 4.10m | FBgn0035498 | FBtr0073210 | Dme.3L.1 |  |
| Caenorhabditis elegans | | V: 14.69m | WBGene00006836 | C47E8.7.2 | Cel.V |  |
|  |  |  |  |  |  |  |
| **Species** | **HGNC,ZFIN or FlyBase name** | **Chromosome/scaffold locations** | **Ensembl ID or NCBI accession number** | **Transcript ID** | **Assigned sequence name** | **Comments/Annotation notes** |
| Human | JAG2 | 14: 105.14m | ENSG00000184916 | ENST00000331782.7 | Hsa.14 |  |
|  | JAG1 | 20: 10.64m | ENSG00000101384 | ENST00000254958.9 | Hsa.20 |  |
| Chicken |  | 3: 13.51m | ENSGALG00000009020 | ENSGALT00000014678.6 | Gga.3 |  |
|  |  | 5: 52.20m | ENSGALG00000011696 | ENSGALT00000021553.4 | Gga.5 |  |
| Coelacanth | | JH126795.1: 11.26k | ENSLACG00000001168 | ENSLACT00000001314.1 | Lch.JH126795 |  |
|  |  | JH126564.1: 775.13k | ENSLACG00000012867 | ENSLACT00000014718.1 | Lch.JH126564 |  |
| Spotted gar | | LG16: 6.35m | ENSLOCG00000015958 | ENSLOCT00000019689.1 | LocLG16 |  |
|  |  | LG2: 66.41m | ENSLOCG00000014652 | ENSLOCT00000018067.1 | Loc.LG2 | assigned family member name in tree: JAG3 |
|  |  | LG7: 7.81m | ENSLOCG00000010321 | ENSLOCT00000012701.1 | Loc.LG7 |  |
| Zebrafish | jag1a | 1: 50.46m | ENSDARG00000030289 | ENSDART00000137172.2 | Dre.1 |  |
|  | jag2a | 13: 33.38m | ENSDARG00000014246 | ENSDART00000025007.8 | Dre.13.1 |  |
|  | jag1b | 13: 35.21m | ENSDARG00000013168 | ENSDART00000019323.6 | Dre.13.2 |  |
|  | CABZ01040999.1 | 18: 35.30m | ENSDARG00000068104 | ENSDART00000098297.4 | Dre.18 | Assigned gene name in this study: jag3 |
|  | jag2b | 20: 21.49m | ENSDARG00000021389 | ENSDART00000024922.6 | Dre.20 |  |
| Ciona intestinalis |  | 11: 5.04m | ENSCING00000021217 | ENSCINT00000031505.1 | Cin.11 |  |
| Ciona savignyi |  | reftig_13: 1628.86k | ENSCSAVG00000006943 | ENSCSAVT00000011971.1 | Csa.ref13 |  |
| Fruitfly |  | 3R: 27.17m | FBgn0004197 | FBtr0085128 | Dme.3R |  |
|  |  |  |  |  |  |  |
| **Species** | **HGNC,ZFIN or FlyBase name** | **Chromosome/scaffold locations** | **Ensembl ID or NCBI accession number** | **Transcript ID** | **Assigned sequence name** | **Comments/Annotation notes** |
| Human | LTBP1 | 2: 32.95m | ENSG00000049323 | ENST00000418533.6 | Hsa.2 |  |
|  | LTBP2 | 14: 74.50m | ENSG00000119681 | ENST00000261978.8 | Hsa.14 |  |
|  | LTBP3 | 11: 65.54m | ENSG00000168056 | ENST00000322147.8 | Hsa.11 |  |
|  | LTBP4 | 19: 40.59m | ENSG00000090006 | ENST00000204005.13 | Hsa.19 |  |
| Chicken |  | 5: 37.97m | ENSGALG00000010448 | ENSGALT00000017012.5 | Gga.5 |  |
|  |  | 3: 31.21m | ENSGALG00000010258 | ENSGALT00000080003.1 | Gga.3 |  |
| Anole lizard |  | NW_003340183.1:0.04m | XP_008122757.1 |  | Aca.NW_003340183 | may-16, manually edited |
|  |  | LGf: 1900.62k | ENSACAG00000003759 | ENSACAT00000003884.3 | Aca.LGf |  |
|  |  | 1: 231.43m | ENSACAG00000009007 | ENSACAT00000009266.3 | Aca.1.2 |  |
|  |  | 1: 22.97m | ENSACAG00000016494 | ENSACAT00000016615.3 | Aca.1.1 |  |
| Coelacanth | | Scaffold JH126663.1: 1780.03k | ENSLACG00000016869 | ENSLACT00000019309.1 | Lch.JH126663 |  |
|  |  | Scaffold JH128017.1: 47.88k | ENSLACG00000002385 | ENSLACT00000002686.1 | Lch.JH128017 | manually edited |
|  |  | Scaffold JH126700.1: 531.11k | ENSLACG00000010764 | ENSLACT00000012320.1 | Lch.JH126700 |  |
|  |  | NW_005819014.1:4.28m | XP_014341338.1 |  | Lch.NW_005819014 | OCT-2015 |
| Spotted Gar | | LG2:64.22m | XP_015196417.1 |  | Loc.LG2 | jan-16 |
|  |  | LG7: 8.92m | ENSLOCG00000010749 | ENSLOCT00000013204.1 | Loc.LG7 |  |
|  |  | LG16: 16.61m | ENSLOCG00000016686 | ENSLOCT00000020650.1 | Loc.LG16 |  |
|  |  | LG28:3.62m | XP_015194379.1 |  | Loc.LG28 | jan-16, manually edited |
| Zebrafish | ltbp4 | 18: 49.01m | ENSDARG00000099137 | ENSDART00000160702.1 | Dre.18 | Assigned gene name in this study: ltbp4 |
|  | ltbp3 | 5: 36.92m | ENSDARG00000035682 | ENSDART00000048107.7 | Dre.5 |  |
|  | ltbp1 | 17: 22.94m | ENSDARG00000056922 | ENSDART00000079460.5 | Dre.17 |  |
| Medaka |  | 22: 13.25m | ENSORLG00000016691 | ENSORLT00000020883.1 | Ola.22 |  |
| Fruitfly |  | 4: 215.01k | FBgn0051999 | FBtr0089144 | Dme.4 |  |
| Caenorhabditis elegans | | IV: 9.54m | WBGene00001403 | F56H11.1g | Cel.IV |  |
|  |  | III: 7.63m | WBGene00022816 | ZK783.1k | Cel.III |  |
| Human | FBLN1 | 22: 45.50m | ENSG00000077942 | ENSG00000077942 | Hsa.FBLN1 |  |
|  | FBLN2 | 3: 13.55m | ENSG00000163520 | ENST00000295760.11 | Hsa.FBLN2 |  |
|  | FBLN5 | 14: 91.87m | ENSG00000140092 | ENST00000342058.8 | Hsa.FBLN5 |  |
|  | EFEMP1 | 2: 55.87m | ENSG00000115380 | ENST00000394555.6 | Hsa.EFEMP1 |  |
|  | EFEMP2 | 11: 65.87m | ENSG00000172638 | ENST00000307998.10 | Hsa.EFEMP2 |  |
|  |  |  |  |  |  |  |
| **Species** | **HGNC,ZFIN or FlyBase name** | **Chromosome/scaffold locations** | **Ensembl ID or NCBI accession number** | **Transcript ID** | **Assigned sequence name** | **Comments/Annotation notes** |
| Human | MERTK | 2: 111.90m | ENSG00000153208 | ENST00000421804.6 | Hsa.2 |  |
|  | TYRO3 | 15: 41.56m | ENSG00000092445 | ENST00000263798.7 | Hsa.15 |  |
|  | AXL | 19: 41.22m | ENSG00000167601 | ENST00000359092.7 | Hsa.19 |  |
| Chicken |  | 3: 3.20m | ENSGALG00000008257 | ENSGALT00000013438.4 | Gga.3 |  |
|  |  | 5: 24.92m | ENSGALG00000008631 | ENSGALT00000014067.5 | Gga.5 |  |
| Anole lizard |  | GL343437.1: 208.91k | ENSACAG00000005886 | ENSACAT00000006091.3 | Aca.GL343437 |  |
|  |  | LGf: 3.52m | ENSACAG00000003874 | ENSACAT00000003990.3 | Aca.LGf |  |
|  |  | 1: 41.10m | ENSACAG00000014118 | ENSACAT00000014314.3 | Aca.1 |  |
| Coelacanth | | JH128421.1: 230.21k | ENSLACG00000006674 | ENSLACT00000007591.1 | Lch.JH128421 | assigned family member name in tree: novel |
|  |  | JH126731.1: 429.73k | ENSLACG00000009552 | ENSLACT00000010931.1 | Lch.JH126731 |  |
|  |  | JH130650.1: 27.74k | ENSLACG00000001709 | ENSLACT00000001925.1 | Lch.JH130650 | *manually edited* |
|  |  | JH126578.1: 2.53m | ENSLACG00000017950 | ENSLACT00000020563.1 | Lch.JH126578 |  |
| Spotted gar | | LG16: 15.18m | ENSLOCG00000016537 | ENSLOCT00000020464.1 | Loc.LG16 |  |
|  |  | LG2: 61.92m | ENSLOCG00000014171 | ENSLOCT00000017488.1 | Loc.LG2 | *manually edited* |
|  |  | LG7: 11.69m | ENSLOCG00000011682 | ENSLOCT00000014385.1 | Loc.LG7 |  |
| Zebrafish | tyro3 | 17: 10.35m | ENSDARG00000005356 | ENSDART00000140391.2 | Dre.17 |  |
|  | mertka | 13: 47.33m | ENSDARG00000074695 | ENSDART00000109266.3 | Dre.13 |  |
|  | AXL | 15:0.05m | XP_017206598.2 |  | Dre.15 | may-17. Described as AXL at Ensembl and NCBI |
|  | si:ch73-40a2.1 | 7: 17.82m | ENSDARG00000105521 | ENSDART00000173689.1 | Dre.7 | assigned family member name in synteny figure and tree: novel |
| Ciona intestinalis |  | 14: 149.24k | ENSCING00000000360 | ENSCINT00000027120.2 | Cin.14 |  |
| Ciona savignyi |  | reftig_49: 261.92k | ENSCSAVG00000007251 | ENSCSAVT00000012473.1 | Csa.ref49 |  |
| Caenorhabditis elegans | | X: 6.55m | WBGene00020504 | T14E8.1a | Cel.X |  |
|  |  |  |  |  |  |  |
| **Species** | **HGNC,ZFIN or FlyBase name** | **Chromosome/scaffold locations** | **Ensembl ID or NCBI accession number** | **Transcript ID** | **Assigned sequence name** | **Comments/Annotation notes** |
| Human | NRXN1 | 2: 49.92m | ENSG00000179915 | ENST00000404971.5 | Hsa.2 |  |
|  | NRXN2 | 11: 64.61m | ENSG00000110076 | ENST00000377559.7 | Hsa.11 |  |
|  | NRXN3 | 14: 78.17m | ENSG00000021645 | ENST00000635466.1 | Hsa.14 |  |
| Chicken |  | 3: 6.56m | ENSGALG00000009107 | ENSGALT00000047522.1 | Gga.3 |  |
|  |  | 5:39.57m | NP_001258852.1 |  | Gga.5 | oct-17 |
| Chinese softshell turtle | | NW_005853176.1:0.91m | XP_006116042.1 |  | Psi.NW_005853176 | nov-15 |
|  |  | NW_005854898.1:1.36m | XP_014428577.1 |  | Psi.NW_005854898 | nov-15 |
|  |  | JH211041.1: 591.14k | ENSPSIG00000003726 | ENSPSIT00000003973.1 | Psi.JH211041 |  |
| Coelacanth | | JH126696.1: 1879.38k | ENSLACG00000017041 | ENSLACT00000019509.1 | Lch.JH126696 | *short* |
|  |  | JH126618.1: 75.29k | ENSLACG00000003239 | ENSLACT00000003669.1 | Lch.JH126618 | *manually edited,short* |
|  |  | JH126618.1: 539.47k | ENSLACG00000010836 | ENSLACT00000012400.1 |  |  |
|  |  | JH129271.1: 97.62k | ENSLACG00000003804 | ENSLACT00000004310.1 | Lch.JH129271 | *short* |
|  |  | JH127406.1: 291.56k | ENSLACG00000007669 | ENSLACT00000008743.1 | Lch.JH127406 | *short* |
|  |  | NW_005819260.1:0.44m | XP_005995332.1 |  | Lch.NW_005819260 | oct-15 |
| Spotted gar | | LG7: 2.03m | ENSLOCG00000008835 | ENSLOCT00000010797.1 | Loc.LG7 |  |
|  |  | LG16: 11.51m | ENSLOCG00000016276 | ENSLOCT00000020112.1 | Loc.LG16 |  |
|  |  | LG28: 2.48m | ENSLOCG00000002397 | ENSLOCT00000002886.1 | Loc.LG28 | manually edited |
|  |  | LG28: 3.02m | ENSLOCG00000002471 | ENSLOCT00000002909.1 |  | manually edited |
| Zebrafish | nrwn3a | 17:16.91m | NP_001073478.1 |  | Dre.17 | dec-17 |
|  | nrxn3b | 20: 5.52m | ENSDARG00000062693 | ENSDART00000090934.5 | Dre.20 |  |
|  | nrxn1a | 12: 24.22m | ENSDARG00000061647 | ENSDART00000093094.6 | Dre.12 |  |
|  | nrxn1b | 13: 602.74k | ENSDARG00000063635 | ENSDART00000149547.2 | Dre.13 |  |
|  | nrxn2a | 21: 27.60m | ENSDARG00000061454 | ENSDART00000141629.2 | Dre.21 |  |
|  | nrxn2b | 7:0.43m | NP_001073453.1 |  | Dre.7 | aug-17 |
| Ciona intestinalis |  | 3: 962.05k | ENSCING00000005088 | ENSCINT00000010501.3 | Cin.3 |  |
| Fruitfly | Nrx-1 | 3R: 22.47m | FBgn0038975 | FBtr0334610 | Dme.3R |  |
|  |  |  |  |  |  |  |
| **Species** | **HGNC,ZFIN or FlyBase name** | **Chromosome/scaffold locations** | **Ensembl ID or NCBI accession number** | **Transcript ID** | **Assigned sequence name** | **Comments/Annotation notes** |
| Human | PLD3 | 19: 40.35m | ENSG00000105223 | ENST00000356508.9 | Hsa.19 |  |
|  | PLD4 | 14: 104.92m | ENSG00000166428 | ENST00000392593.8 | Hsa.14 |  |
|  | PLD5 | 1: 242.08m | ENSG00000180287 | ENST00000536534.6 | Hsa.1 |  |
| Chicken |  | Scaffold NT_465812.1:0.01m | XP_015129279.1 |  | Gga.scaffold | jan-16 |
|  |  | 3: 35.58m | ENSGALG00000010737 | ENSGALT00000047039.1 | Gga.3 |  |
|  |  | 5: 51.87m | ENSGALG00000011646 | ENSGALT00000019035.6 | Gga.5 |  |
| Chinese softshell turtle | | Scaffold JH208232.1: 42.70k | ENSPSIG00000008356 | ENSPSIT00000009316.1 | Psi.JH208232 |  |
|  |  | Scaffold JH211376.1: 990.76k | ENSPSIG00000009358 | ENSPSIT00000010449.1 | Psi.JH211376 |  |
|  |  | Scaffold JH212505.1: 55.98k | ENSPSIG00000004919 | ENSPSIT00000005308.1 | Psi.JH212505 |  |
|  |  | Scaffold JH206113.1: 49.05k | ENSPSIG00000003872 | ENSPSIT00000004138.1 | Psi.JH206113 | assigned family member name in tree: pld7 |
| Coelacanth | | Scaffold JH126593.1: 228.36k | ENSLACG00000006642 | ENSLACT00000007554.1 | Lch.JH126593 | assigned family member name in tree: pld7 |
|  |  | Scaffold JH126986.1: 327.52k | ENSLACG00000008209 | ENSLACT00000009378.1 | Lch.JH126986 |  |
|  |  | Scaffold JH126564.1: 350.31k | ENSLACG00000008533 | ENSLACT00000009752.1 | Lch.JH126564 | manually edited |
|  |  | Scaffold JH126663.1: 2.50m | ENSLACG00000017919 | ENSLACT00000020531.1 | Lch.JH126663 |  |
| Spotted gar | | LG28: 699.07k | ENSLOCG00000001445 | ENSLOCT00000001659.1 | Loc.LG28 | Assigned gene name in this study: pld7 |
|  |  | LG16: 76.54k | ENSLOCG00000015383 | ENSLOCT00000018963.1 | Loc.LG16 |  |
|  |  | LG7: 16.44m | ENSLOCG00000012785 | ENSLOCT00000015762.1 | Loc.LG7 |  |
|  |  | LG2: 67.62m | ENSLOCG00000014810 | ENSLOCT00000018266.1 | Loc.LG2 |  |
| Zebrafish | si:dkeyp-110e4.6 | 14: 26.13m | ENSDARG00000061845 | ENSDART00000088677.5 | Dre.14 | Assigned gene name in this study: pld7 |
|  | si:ch211-194e18.2 | 6: 6.24m | ENSDARG00000063257 | ENSDART00000092257.5 | Dre.6 |  |
|  | pld3 | 18: 46.27m | ENSDARG00000068199 | ENSDART00000131724.1 | Dre.18 |  |
|  | pld4 | 17:1.63m | XP_021322934.1 |  | Dre.17 | jun-17, manually edited |
| Amphioxus | | NW_003101358.1:1.01m | XP_002589021.1 |  | Bfl.NW_003101358.1 | oct-09 |
|  |  | NW_003101358.1:1.07m | XP_002589028.1 |  | Bfl.NW_003101358.2 | oct-09 |
| Fruitfly | CG43345 | 2L: 21.17m | FBgn0263050 | FBtr0346589 | Dme.2L.1 |  |
|  | CG9248 | 2L: 21.16m | FBgn0032923 | FBtr0343870 | Dme.2L.2 |  |
| Caenorhabditis elegans | | V: 5.49m | WBGene00020256 | T05C3.6a | Cel.V.1 |  |
|  |  | II: 7.19m | WBGene00017124 | E04F6.4 | Cel.II |  |
|  |  | V: 18.57m | WBGene00013080 | Y51A2D.13b | Cel.V.3 |  |
|  |  | V: 7.19m | WBGene00017316 | F09G2.8b | Cel.V.2 |  |
|  |  |  |  |  |  |  |
| **Species** | **HGNC,ZFIN or FlyBase name** | **Chromosome/scaffold locations** | **Ensembl ID or NCBI accession number** | **Transcript ID** | **Assigned sequence name** | **Comments/Annotation notes** |
| Human | PRKD1 | 14: 29.58m | ENSG00000184304 | ENST00000415220.6 | Hsa.14 |  |
|  | PRKD2 | 19: 46.67m | ENSG00000105287 | ENST00000433867.5 | Hsa.19 |  |
|  | PRKD3 | 2: 37.25m | ENSG00000115825 | ENST00000379066.5 | Hsa.2 |  |
| Chicken |  | 5: 33.85m | ENSGALG00000009900 | ENSGALT00000057736.2 | Gga.5 | edited manually |
|  |  | 3: 33.63m | ENSGALG00000010612 | ENSGALT00000017268.4 | Gga.3 |  |
| Chinese softshell turtle | | Scaffold JH207692.1: 8.54k | ENSPSIG00000016551 | ENSPSIT00000018815.1 | Psi.JH207692 | edited manually |
|  |  | Scaffold JH208684.1: 208.22k | ENSPSIG00000010601 | ENSPSIT00000012146.1 | Psi.JH208684 | edited manually |
|  |  | Scaffold JH212505.1: 3.64m | ENSPSIG00000006040 | ENSPSIT00000006690.1 | Psi.JH212505 | edited manually |
|  |  | Scaffold JH207378.1: 335.67k | ENSPSIG00000007942 | ENSPSIT00000008981.1 | Psi.JH207378 | Assigned family member name in tree : PRKD4 |
| Coelacanth | | Scaffold JH128496.1: 178.03k | ENSLACG00000005649 | ENSLACT00000006419.1 | Lch.JH128496 | edited manually |
|  |  | Scaffold JH127173.1: 150.88k | ENSLACG00000005076 | ENSLACT00000005762.1 | Lch.JH127173 | edited manually |
|  |  | Scaffold JH126749.1: 590.35k | ENSLACG00000011315 | ENSLACT00000012945.1 | Lch.JH126749 | edited manually |
| Spotted gar | | LG7: 5.50m | ENSLOCG00000009700 | ENSLOCT00000011878.1 | Loc.LG7 |  |
|  |  | LG2: 68.48m | ENSLOCG00000014916 | ENSLOCT00000018401.1 | Loc.LG2 | edited manually |
|  |  | LG1: 50.25m | ENSLOCG00000016864 | ENSLOCT00000020882.1 | Loc.LG1 |  |
|  |  | LG28: 881.06k | ENSLOCG00000001570 | ENSLOCT00000001812.1 | Loc.LG28 | Assigned gene name in this study: PRKD4 |
| Zebrafish | prkd1 | 17: 28.87m | ENSDARG00000075949 | ENSDART00000153937.2 | Dre.17.1 |  |
|  | prkd2 | 15:11.77m | XP_021322000.1 |  | Dre.15 | jun-17 |
|  | prkd3 | 17: 42.34m | ENSDARG00000079967 | ENSDART00000112765.3 | Dre.17.2 |  |
|  | zgc:175248 | 7: 6.88m | NP_001170925.1 |  | Dre.7 | Assigned gene name in this study: prkd4 |
| Ciona intestinalis |  | 2: 1376.49k | ENSCING00000011582 | ENSCINT00000022313.2 | Cin.2 |  |
|  |  | 4: 5.33m | ENSCING00000005266 | ENSCINT00000010841.3 | Cin.4 |  |
| Ciona savignyi |  | reftig_19: 2.68m | ENSCSAVG00000009881 | ENSCSAVT00000016979.1 | Csa.ref19 |  |
|  |  | reftig_15: 2.05m | ENSCSAVG00000009491 | ENSCSAVT00000016306.1 | Csa.ref15 |  |
| Fruitfly | PKD | 3R: 18.30m | FBgn0038603 | FBtr0330313 | Dme.3R |  |
| Caenorhabditis elegans | | I: 13.64m | WBGene00012352 | W09C5.5 | Cel.I |  |
|  |  | V: 16,75m | WBGene00012019 | T25E12.4f | Cel.V |  |
|  |  |  |  |  |  |  |
| **Species** | **HGNC,ZFIN or FlyBase name** | **Chromosome/scaffold locations** | **Ensembl ID or NCBI accession number** | **Transcript ID** | **Assigned sequence name** | **Comments/Annotation notes** |
| Human | PROX1 | 1: 213.98m | ENSG00000117707 | ENST00000366958.8 | Hsa.1 |  |
|  | PROX2 | 14: 74.85m | ENSG00000119608 | ENST00000556489.3 | Hsa.14 |  |
| Chicken |  | 5: 38.13m | XP_015143173.1 |  | Gga.5 | nov-15 |
|  |  | 3: 21.31m | ENSGALG00000009791 | ENSGALT00000015927.5 | Gga.3 |  |
| Chinese softshell turtle | | scaffold NW_005857314.1: 0.10m | XP_014432861.1 |  | Psi.NW_005857314 | nov-15 |
|  |  | scaffold NW_005859004.1: 0.50m | XP_006136626.1 |  | Psi.NW_005859004 | nov-15., manually edited. Assigned family member name in tree : PROX3 |
|  |  | scaffold NW_005853657.1: 6.51m | XP_014426287.1 |  | Psi.NW_005853657 | Nov-15. manually edited |
| Coelacanth | | Scaffold JH127021.1: 896.11k | ENSLACG00000013691 | ENSLACT00000015662.1 | Lch.JH127021 | manually edited |
|  |  | Scaffold JH126565.1: 5.25m | ENSLACG00000018909 | ENSLACT00000021660.1 | Lch.JH126565 |  |
| Spotted gar | | LG1: 44.46m | ENSLOCG00000016698 | ENSLOCT00000020666.1 | Loc.LG1 |  |
|  |  | LG7: 8.79m | ENSLOCG00000010701 | ENSLOCT00000013140.1 | Loc.LG7 |  |
|  |  | LG28: 1980.83k | ENSLOCG00000002197 | ENSLOCT00000002563.1 | Loc.LG28 | Assigned gene name in this study: prox3 |
| Zebrafish | prox1b | 7: 19.68m | ENSDARG00000088810 | ENSDART00000127669.3 | Dre.7 | Assigned gene name in this study: prox3 |
|  | prox1a | 17: 32.87m | ENSDARG00000055158 | ENSDART00000172016.1 | Dre.17.1 | Assigned gene name in this study: prox1 |
|  | prox2 | 17: 52.41m | ENSDARG00000041952 | ENSDART00000114931.3 | Dre.17.2 |  |
| Ciona intestinalis |  | 4: 528.34k | ENSCING00000008999 | ENSCINT00000018262.3 | Cin.4.1 |  |
|  |  | 4: 543.58k | ENSCING00000013650 | ENSCINT00000025222.2 | Cin.4.2 |  |
| Ciona savignyi |  | reftig_15: 1373.97k | ENSCSAVG00000007613 | ENSCSAVT00000013114.1 | Csa.ref15 |  |
| Caenorhabditis elegans | | III: 8.07m | WBGene00000448 | K12H4.1 | Cel.III |  |
|  |  |  |  |  |  |  |
| **Species** | **HGNC,ZFIN or FlyBase name** | **Chromosome/scaffold locations** | **Ensembl ID or NCBI accession number** | **Transcript ID** | **Assigned sequence name** | **Comments/Annotation notes** |
| Human | PRPH2 | 6: 42.70m | ENSG00000112619 | ENST00000230381.6 | Hsa.6. |  |
|  | ROM1 | 11: 62.61m | ENSG00000149489 | ENST00000278833.3 | Hsa.11 |  |
| Chicken |  | 3: 23.09m | ENSGALG00000009909 | ENSGALT00000016117.2 | Gga.3 |  |
|  |  | 5: 25.97m | ENSGALG00000009185 | ENSGALT00000014960.1 | Gga.5 | Assigned gene name in this study: PRPH2L |
| Coelacanth | | Scaffold JH126942.1: 768.29k | ENSLACG00000012811 | ENSLACT00000014656.1 | Lch.JH126942 |  |
|  |  | Scaffold JH126593.1: 329.42k | ENSLACG00000008239 | ENSLACT00000009410.1 | Lch.JH126593 |  |
|  |  | Scaffold JH129987.1: 138.23k | ENSLACG00000004767 | ENSLACT00000005409.1 | Lch.JH129987 | Assigned family member name in tree: PRPH2L |
| Spotted gar | | LG16: 14.47m | ENSLOCG00000016479 | ENSLOCT00000020394.1 | Loc.LG16 |  |
|  |  | LG28: 273.81k | ENSLOCG00000001099 | ENSLOCT00000001243.1 | Loc.LG28 |  |
|  |  | LG7: 17.74m | ENSLOCG00000012939 | ENSLOCT00000015964.1 | Loc.LG7 | Assigned gene name in this study: PRPH2L |
| Zebrafish | prph2a | 12: 34.80m | ENSDARG00000038018 | ENSDART00000055415.5 | Dre.12 |  |
|  | prph2b | 13: 3.12m | ENSDARG00000014840 | ENSDART00000020671.8 | Dre.13 |  |
|  | rom1a | 5: 66.45m | ENSDARG00000019752 | ENSDART00000011295.8 | Dre.5 |  |
|  | rom1b | 14: 46.69m | ENSDARG00000026926 | ENSDART00000105461.3 | Dre.14 |  |
|  | prph2l | 20: 27.49m | ENSDARG00000021345 | ENSDART00000010584.4 | Dre.20 | Assigned gene name in this study: prph2lb |
|  | zmp:0000000545 | 17: 39.93m | ENSDARG00000013134 | ENSDART00000002217.4 | Dre.17 | Assigned gene name in this study: prph2la |
| Amphioxus | | NW_003101360.1:0.48m | XP_002589165.1 |  | Bfl.NW_003101360 | oct-09 |
| Purple sea urchin |  | NW_011992578.1:0.22m | XP_011677540.1 |  | Spu.NW_011992578 | mar-15 |
|  |  | NW_011992578.1:0.23m | XP_001197913.1 |  | Spu.NW_011992578 | mar-15 |
|  |  |  |  |  |  |  |
| **Species** | **HGNC,ZFIN or FlyBase name** | **Chromosome/scaffold locations** | **Ensembl ID or NCBI accession number** | **Transcript ID** | **Assigned sequence name** | **Comments/Annotation notes** |
| Human | PYGB | 20: 25.25m | ENSG00000100994 | ENST00000216962.8 | Hsa.20 |  |
|  | PYGL | 14: 50.86m | ENSG00000100504 | ENST00000216392.7 | Hsa.14 |  |
|  | PYGM | 11: 64.75m | ENSG00000068976 | ENST00000164139.3 | Hsa.11 |  |
| Chicken |  | 3: 4.19m | ENSGALG00000008508 | ENSGALT00000039933.2 | Gga.3 |  |
|  |  | 5: 58.10m | ENSGALG00000012365 | ENSGALT00000020196.6 | Gga.5 |  |
| Anole lizard |  | Scaffold GL343850.1: 12.75k | ENSACAG00000013595 | ENSACAT00000014006.3 | Aca.GL343850 | manually edited |
|  |  | 1: 254.33m | ENSACAG00000013998 | ENSACAT00000014189.3 | Aca.1 | manually edited |
|  |  | Scaffold GL344430.1: 9.77k | ENSACAG00000005131 | ENSACAT00000005240.3 | Aca.GL344430 | manually edited |
| Coelacanth | | NW_005819966.1: 0.39m rev | XP_014348577.1 |  | Lch.NW_005819966 | oct-15. manually edited |
|  |  | NW_005819042.1: 3.46m fwd | XP_014354239.1 |  | Lch.NW_005819042 | oct-15. manually edited |
|  |  | Scaffold JH126849.1: 954.96k | ENSLACG00000014055 | ENSLACT00000016073.1 | Lch.JH126849 | manually edited |
| Spotted gar | | LG16: 17.17m | ENSLOCG00000016769 | ENSLOCT00000020772.1 | Loc.LG16 |  |
|  |  | LG28: 1823.12k | ENSLOCG00000002115 | ENSLOCT00000002469.1 | Loc.LG28 | manually edited |
|  |  | LG7: 12.17m | ENSLOCG00000011949 | ENSLOCT00000014731.1 | Loc.LG7 | manually edited |
| Zebrafish | pygma | 21: 28.41m | ENSDARG00000055518 | ENSDART00000077871.4 | Dre.21 |  |
|  | pygmb | 7: 22.55m | ENSDARG00000013317 | ENSDART00000127467.2 | Dre.7 |  |
|  | pygl | 13: 36.72m | ENSDARG00000002197 | ENSDART00000149011.1 | Dre.13 |  |
|  | pygb | 17: 21.11m | ENSDARG00000002021 | ENSDART00000022830.8 | Dre.17 |  |
| Ciona intestinalis |  | Scaffold HT000068.1: 3.10k rev | ENSCING00000006480 | ENSCINT00000013376.3 | Cin.HT000068 |  |
| Ciona savignyi |  | reftig 56: 1.06m fwd | ENSCSAVG00000000173 | ENSCSAVT00000000313.1 | Csa.ref56 |  |
| Amphioxus | | NW_003101565.1: 3.46m rev | XP_002613320.1 |  | Bfl.NW_003101565 | oct-09 |
| Caenorhabditis elegans | | V: 3.58m | WBGene00020696 | T22F3.3b.3 | Cel.V |  |
| Fruitfly | GlyP | Dme.2L | FBgn0004507 | FBtr0077828 | Dme.2L |  |
|  |  |  |  |  |  |  |
| **Species** | **HGNC,ZFIN or FlyBase name** | **Chromosome/scaffold locations** | **Ensembl ID or NCBI accession number** | **Transcript ID** | **Assigned sequence name** | **Comments/Annotation notes** |
| Human | SLC24A3 | 20: 19.21m | ENSG00000185052 | ENST00000328041.10 | Hsa.20 |  |
|  | SLC24A4 | 14: 92.32m | ENSG00000140090 | ENST00000532405.5 | Hsa.14 |  |
| Chicken |  | 3: 3.98m | ENSGALG00000008445 | ENSGALT00000013756.5 | Gga.3 |  |
|  |  | 5: 44.71m | ENSGALG00000010793 | ENSGALT00000017553.5 | Gga.5 |  |
| Coelacanth | | JH126565.1: 6.65m | ENSLACG00000019037 | ENSLACT00000021807.1 | Lch.JH126565 |  |
|  |  | JH127769.1: 171.94k | ENSLACG00000005528 | ENSLACT00000006284.1 | Lch.JH127769 |  |
| Spotted gar | | LG28: 3.53m | ENSLOCG00000002679 | ENSLOCT00000003170.1 | Loc.LG28 | Assigned gene name in this study: SLC2416 |
|  |  | LG16: 10.12m | ENSLOCG00000016155 | ENSLOCT00000019947.1 | Loc.LG16 |  |
|  |  | LG7: 32.71m | ENSLOCG00000014107 | ENSLOCT00000017416.1 | Loc.LG7 |  |
| Zebrafish | slc24a4a | 17: 38.84m | ENSDARG00000015425 | ENSDART00000141177.1 | Dre.17 |  |
|  | slc24a4b | 20: 50.31m | ENSDARG00000067509 | ENSDART00000031139.8 | Dre.20 |  |
|  | slc24a3 | 13: 26.94m | ENSDARG00000006760 | ENSDART00000146227.1 | Dre.13 |  |
|  |  | 7: 71.80m | ENSDARG00000041086 | ENSDART00000017763.8 | Dre.7 | Assigned gene name in this study: slc24a6b |
|  | si:ch73-335m24.5 | 14: 33.56m | ENSDARG00000036383 | ENSDART00000112438.5 | Dre.14 | Assigned gene name in this study: slc24a6a |
| Ciona intestinalis |  | Scaffold HT000052.1: 81.93k | ENSCING00000001025 | ENSCINT00000001908.3 | Cin.HT000052 |  |
| Ciona savignyi |  | reftig_1596: 4.26k | ENSCSAVG00000003510 | ENSCSAVT00000005949.1 | Csa.ref1596 |  |
| Amphioxus | | NW_003101527.1:0.53m | XP_002606620.1 |  | Bfl.NW_003101527 | oct-2009 |
|  |  | NW_003101373.1:0.46m | XP_002590091.1 |  | Bfl.NW_003101373 | oct-2009 |
|  |  | NW_003101410.1:0.79m | XP_002593527.1 |  | Bfl.NW_003101410 | oct-2009 |
| Fruitfly | zyd | X: 23.02m | FBgn0265767 | FBtr0301133 | Dme.X.1 |  |
|  | CG12061 | X: 23.04m | FBgn0040031 | FBtr0332625 | Dme.X.2 |  |
|  | CG17167 | X: 23.07m | FBgn0039941 | FBtr0300807 | Dme.X.3 |  |
|  | CG1090 | 3R: 4.36m | FBgn0037238 | FBtr0335201 | Dme.3R |  |
| Human | SLC24A5 | 15: 48.12m | ENSG00000188467 | ENST00000341459.7 | Hsa.SLC24A5 |  |
|  | SLC24A1 | 15: 65.61m | ENSG00000074621 | ENST00000261892.10 | Hsa.SLC24A1 |  |
|  | SLC24A2 | 9: 19.51m | ENSG00000155886 | ENST00000341998.6 | Hsa.SLC24A2 |  |
|  |  |  |  |  |  |  |
| **Species** | **HGNC,ZFIN or FlyBase name** | **Chromosome/scaffold locations** | **Ensembl ID or NCBI accession number** | **Transcript ID** | **Assigned sequence name** | **Comments/Annotation notes** |
| Human | SPTB | 14: 64.75m | ENSG00000070182 | ENST00000389722.7 | Hsa.14 |  |
|  | SPTBN4 | 19: 40.47m | ENSG00000160460 | ENST00000598249.5 | Hsa.19 |  |
|  | SPTBN2 | 11: 66.69m | ENSG00000173898 | ENST00000309996.6 | Hsa.11 |  |
|  | SPTBN1 | 2: 54.46m | ENSG00000115306 | ENST00000356805.8 | Hsa.2 |  |
| Chicken |  | 3: 2.70m | ENSGALG00000029512 | ENSGALT00000057896.1 | Gga.3 |  |
|  |  | Scaffold AADN04004592.1: 1,366 | ENSGALG00000041560 | ENSGALT00000076960.1 | Gga.AADN04004592.1 | manually edited |
|  |  | 5: 311.49k | ENSGALG00000036805 | ENSGALT00000056882.1 | Gga.5 |  |
|  |  | Scaffold NT_464134.1:0.07m | XP_015128474.1 |  | Gga.NT_464134 | jan-16 |
| Anolis lizard |  | 1: 263.40m | ENSACAG00000011972 | ENSACAT00000012304.3 | Aca.1.2 |  |
|  |  | GL343961.1: 63.38k | ENSACAG00000007494 | ENSACAT00000007763.3 | Aca.GL343961 |  |
|  |  | LGf: 2.00m | ENSACAG00000003147 | ENSACAT00000003648.3 | Aca.LGf |  |
|  |  | 1: 93.84m | ENSACAG00000009068 | ENSACAT00000009339.3 | Aca.1.1 |  |
| Coelacanth | | JH126660.1: 1911.64k | ENSLACG00000017110 | ENSLACT00000019589.1 | Lch.JH126660 |  |
|  |  | JH126663.1: 2.19m | ENSLACG00000017554 | ENSLACT00000020107.1 | Lch.JH126663 |  |
|  |  | JH126593.1: 3.17m | ENSLACG00000018388 | ENSLACT00000021069.1 | Lch.JH126593 |  |
|  |  | JH127271.1: 900.75k | ENSLACG00000013725 | ENSLACT00000015698.1 | Lch.JH127271 |  |
| Spotted gar | | LG16: 2.28m | ENSLOCG00000015528 | ENSLOCT00000019156.1 | Loc.LG16 |  |
|  |  | LG28: 2.29m | ENSLOCG00000002333 | ENSLOCT00000002734.1 | Loc.LG28 | manually edited |
|  |  | LG28: 2.30m | ENSLOCG00000002344 | ENSLOCT00000002752.1 |  |  |
|  |  | LG2: 64.33m | ENSLOCG00000014564 | ENSLOCT00000017962.1 | Loc.LG2 |  |
|  |  | LG7: 19.70m | ENSLOCG00000013491 | ENSLOCT00000016682.1 | Loc.LG7 |  |
| Zebrafish | sptb | 17: 38.62m | ENSDARG00000030490 | ENSDART00000040627.5 | Dre.17 |  |
|  | si:ch73-262h23.4 | 18: 46.03m | ENSDARG00000092624 | ENSDART00000078561.5 | Dre.18 | Assigned gene name in this study: sptbn4b |
|  | si:dkey-27p18.5 | 5: 21.96m | ENSDARG00000068624 | ENSDART00000133174.1 | Dre.5.1 | Assigned gene name in this study: sptbn4a |
|  | sptbn2 | 5: 37.05m | ENSDARG00000053956 | ENSDART00000162929.1 | Dre.5.2 |  |
|  | sptbn1 | 11: 42.84m | ENSDARG00000102883 | ENSDART00000164700.1 | Dre.11 |  |
| Ciona intestinalis |  | 3: 5.13m | ENSCING00000008947 | ENSCINT00000018184.3 | Cin.3 |  |
| Ciona savignyi |  | reftig_1: 4.53m | ENSCSAVG00000011411 | ENSCSAVT00000019668.1 | Csa.ref1 |  |
| Fruitfly | beta-Spec | X: 17.66m | FBgn0250788 | FBtr0334404 | Dme.X |  |
| Caenorhabditis elegans | | V: 6.88m | WBGene00006803 | K11C4.3c | Cel.V |  |
|  |  |  |  |  |  |  |
| **Species** | **HGNC,ZFIN or FlyBase name** | **Chromosome/scaffold locations** | **Ensembl ID or NCBI accession number** | **Transcript ID** | **Assigned sequence name** | **Comments/Annotation notes** |
| Human | TGFB1 | 19: 41.30m | ENSG00000105329 | ENST00000221930.5 | Hsa.19 |  |
|  | TGFB2 | 1: 218.35m | ENSG00000092969 | ENST00000366930.8 | Hsa.1 |  |
|  | TGFB3 | 14: 75.96m | ENSG00000119699 | ENST00000238682.7 | Hsa.14 |  |
| Chicken |  | 3: 19.37m | ENSGALG00000009612 | ENSGALT00000031945.4 | Gga.3 |  |
|  |  | 5: 38.50m | ENSGALG00000010346 | ENSGALT00000037707.4 | Gga.5 |  |
|  |  | 32: 24.39k | NP_001305385.1 |  | Gga.32 | aug-17 |
| Anole lizard |  | Chromosome LGf: 3.76m | ENSACAG00000023287 | ENSACAT00000027756.2 | Aca.LGf |  |
|  |  | Chromosome 1: 242.49m | ENSACAG00000001183 | ENSACAT00000001108.3 | Aca.1.2 |  |
|  |  | Chromsome 1: 21.99m | ENSACAG00000017020 | ENSACAT00000017101.3 | Aca.1.1 |  |
| Coelacanth | | Scaffold JH126578.1: 2.81m | ENSLACG00000018146 | ENSLACT00000020792.1 | Lch.JH126578 |  |
|  |  | Scaffold JH127048.1: 240.06k | ENSLACG00000006847 | ENSLACT00000007797.1 | Lch. JH127048 |  |
|  |  | Scaffold JH126565.1: 1664.07k | ENSLACG00000016642 | ENSLACT00000019045.1 | Lch.JH126565 |  |
|  |  | Scaffold JH126593.1: 1968.39k | ENSLACG00000017213 | ENSLACT00000019712.1 | Lch.JH126593 | Assigned family member name in tree: TGFB5 |
| Spotted gar | | LG2: 61.85m | ENSLOCG00000014160 | ENSLOCT00000017474.1 | Loc.LG2 |  |
|  |  | LG28: 402.53k | ENSLOCG00000001175 | ENSLOCT00000001332.1 | Loc.LG28 | manually edited. Assigned gene name in this study: TGFB5 |
|  |  | LG1: 18.27m | ENSLOCG00000015921 | ENSLOCT00000019635.1 | Loc.LG1 |  |
|  |  | LG7: 8.44m | ENSLOCG00000010588 | ENSLOCT00000013005.1 | Loc.LG7 |  |
| Zebrafish | tgfb1a | 15: 2.81m | ENSDARG00000041502 | ENSDART00000060839.2 | Dre.15 |  |
|  | tgfb1b | 21: 21.58m | ENSDARG00000034895 | ENSDART00000134907.1 | Dre.21 |  |
|  | tgfb2l | 14: 8.46m | ENSDARG00000071879 | ENSDART00000106671.4 | Dre.14 | Assigned gene name in this study: tgfb5 |
|  | tgfb3 | 17: 51.99m | ENSDARG00000019367 | ENSDART00000019766.8 | Dre.17 |  |
|  | tgfb2 | 16: 2.75m | NP_919366.1 |  | Dre.16 | dec-18 |
| Ciona intestinalis |  | 3: 6.26m | ENSCING00000008565 | ENSCINT00000017469.3 | Cin.3 |  |
| Ciona savignyi |  | reftig_107: 917.85k | ENSCSAVG00000002907 | ENSCSAVT00000004952.1 | Csa.ref107 |  |
| Fruitfly | daw | 2L: 2.81m | FBgn0031461 | FBtr0335151 | Dme.2L |  |
