# Supplementary material for: Evolution of the Muscarinic Acetylcholine Receptors in Vertebrates
Source: eNeuro. 2018 Nov 8;5(5):ENEURO.0340-18.2018. doi: 10.1523/ENEURO.0340-18.2018 (PMC6298421; doi:10.1523/ENEURO.0340-18.2018)
Supplement: Figure 4-5 — Information about the teleost mAChR amino acid sequences included in the analysis of teleost-specific intron gains. First, the genome assembly versions used are stated, followed by information about the teleost mAChR sequences included in the analysis in the following order: species, HGNC/ZFIN/Flybase symbol name, chromosome or genomic scaffold position, Ensembl ID or NCBI accession number, assigned sequence name, and additional comments regarding sequence update date on NCBI or whether there have been manual edits of the original Ensembl or NCBI sequence. Download Fig. 4-5, DOCX file. [file sup_enu-eN-NWR-0340-18-s15.docx]

| **Ens87** | **Common name** | **Scientific name** | **Assembly Ensembl** | **Assembly NCBI** |  |  |  |
| --- | --- | --- | --- | --- | --- | --- | --- |
|  | European Eel | Anguilla Anguilla |  | Anguilla_anguilla_v1_09_nov_10 |  |  |  |
|  | Zebrafish | Danio rerio | GRCz10 | GRCz11 |  |  |  |
|  | Stickleback | Gasterosteus oculeatus | BROAD S1 |  |  |  |  |
|  | Fugu | Takifugu rubripes |  | FUGU5 |  |  |  |
|  | Medaka | Oryzias latipes | HdrR | ASM223467v1 |  |  |  |
|  | Amazon molly | *Poecilia formosa* | Poecilia_formosa-5.1.2 |  |  |  |  |
|  |  |  |  |  |  |  |  |
|  | **Species** | **HGNC,ZFIN or FlyBase name** | **Chromosome/scaffold locations** | **Ensembl ID or NCBI accession number** | **Transcript ID** | **Assigned sequence name** | **Comments/Annotation notes** |
| **CHRM2b** | Zebrafish | CHRM2b | 25:21.05m | XP_021326218.1 |  | Zebrafish.25.2 | jun-17 |
|  | Stickleback |  | groupXIX: 15.19m | ENSGACG00000011914 | ENSGACT00000015788.1 | Stickleback.XIX.2 | *manually edited* |
|  | Fugu |  | 9: 5.82m | XP_011605476.1 |  | Fugu.9.2 | mar-15 |
|  | Medaka |  | 6: 21.31m | XP_004069752.1 |  | Medaka.6.1 | feb-18 |
|  | Amazon molly |  | KI519623:1.90m | ENSPFOG00000016820 | ENSPFOT00000028635.1 | Amazon molly.KI519623 |  |
| **CHRM3b** | European eel |  | contig_61072 | AZBK01804396.1 |  | European eel.61072 | may-14, *manually edited* |
|  | Zebrafish | CHRM3b | 12: 47.43m | ENSDARG00000071298 | ENSDART00000105331.4 | Zebrafish.12 |  |
|  | Stickleback |  | scaffold_48: 1058.56k | ENSGACG00000015318 | ENSGACT00000020240.1 | Stickleback.scaffold 48 | *manually edited* |
|  | Fugu |  | NW_004071948.1:0.98m | XP_003977030.1 |  | Fugu.NW_004071948 | nov-17 |
|  | Medaka |  | 19: 17.01m | XP_023805573.1 |  | Medaka.19 | feb-17, *manually edited* |
|  | Amazon molly |  | KI520188: 0.16m | ENSPFOG00000022806 | ENSPFOT00000028825.1 | Amazon molly.KI520188 | *manually edited* |
| **CHRM4a** | European eel |  | contig_15756 | AZBK01849712.1 |  | European eel.15756 | may-14, *manually edited* |
|  | Zebrafish | CHRM4a | 7: 38.91m | ENSDARG00000069254 | ENSDART00000100639.4 | Zebrafish.7 | *manually edited* |
|  | Stickleback |  | groupI: 27.13m | ENSGACG00000015349 | ENSGACT00000020282.1 | Stickleback.I | *manually edited* |
|  | Fugu |  | 8:0.61m | XP_003966423.1 |  | Fugu.8 | mar-15 |
|  | Medaka |  | 2: 1.07m | XP_023817375.1 |  | Medaka.2 | feb-18 |
|  | Amazon molly |  | Scaffold KI520734.1: 4.76k | ENSPFOG00000005743 | ENSPFOT00000005654.2 | Amazon molly.KI520734 |  |
| **CHRM4b** | European eel |  | contig_172290 | AZBK01693178.1 |  | European eel.172290 | may-14, *manually edited* |
|  | Zebrafish | CHRM4b | 25: 7.76m | ENSDARG00000017722 | ENSDART00000157276.1 | Zebrafish.25.1 |  |
|  | Stickleback |  | groupXIX: 8.95m | ENSGACG00000007854 | ENSGACT00000010430.1 | Stickleback.XIX.1 | *manually edited* |
|  | Fugu |  | 9:5.54m | XP_003967384.1 |  | Fugu.9.1 | mar-15 |
|  | Medaka |  | 6: 26.80m | XP_011474704.2 |  | Medaka.6.2 | feb-18, *manually edited* |
|  | Amazon molly |  | Scaffold KI519819.1: 187.31k | ENSPFOG00000012741 | ENSPFOT00000012743.2 | Amazon molly.KI519819 |  |
